# Metal-Free Direct C–H Functionalization of Quinoxalin-2(1*H*)-Ones to Produce 3-Vinylated Quinoxalin-2(1*H*)-Ones in the Presence of Alkenes

**DOI:** 10.3389/fchem.2021.672051

**Published:** 2021-04-30

**Authors:** Rongcai Ding, Yingxue Li, Yaoyao Chang, Yue Liu, Jie Yu, Yanna Lv, Jinxing Hu

**Affiliations:** School of Pharmacy, Weifang Medical University, Weifang, China

**Keywords:** vinylation, cross-dehydrocoupling, ammonium persulfate, alkenes, C–H functionalization

## Abstract

A novel and efficient *C*_3_-*H* vinylation reaction with quinoxalin-2(1*H*)-one as the substrate, in the presence of alkenes, under metal-free conditions, is reported herein. The reaction leads to the formation of new carbon–carbon bonds that exhibit moderate to good reactivities. The vinylation of quinoxalin-2(1*H*)-ones, in the presence of alkenes, is an attractive process that can be potentially utilized to produce biologically active 3-vinylated quinoxalin-2(1*H*)-ones.

## Introduction

For Recent years have seen, the emergence of cross-dehydrocoupling (CDC) reaction, between two different molecules, as a prominent research topic (Girard et al., 2014; Huang et al., 2019; Mane et al., 2019; Liu et al., 2020; Xu et al., 2020). These reactions exploit the C-H bonds of various substrates, during the dehydrogenation coupling reactions under oxidizing reaction conditions, to form C-C bonds (Niu et al., 2015; Cheng et al., 2017; Yuan et al., 2018; Xie et al., 2020). The cross-dehydrocoupling reactions provide shorter synthetic routes and new research ideas for the direct and efficient synthesis of complex organic materials from simple raw materials. High atom efficiency can also be achieved (Scheuermann, 2010; Moon et al., 2012; Jiang et al., 2014; Parvatkar et al., 2019).

Quinoxalin-2(1*H*)-ones are important nitrogen-containing fused heterocycles, that form the core structure of numerous biologically active compounds. The biological activity of quinoxalin-2(1H)-one is significantly affected by the substituents present in the core structure of the molecule. The 3-functional quinoxalin-2(1*H*)-ones have been widely studied because they exhibit excellent biological activities (they possess. anti-angiogenic, anti-tumor, and anti-inflammatory properties, among others) (Willardsen et al., 2004; Khattab et al., 2015).

**Scheme 1:**
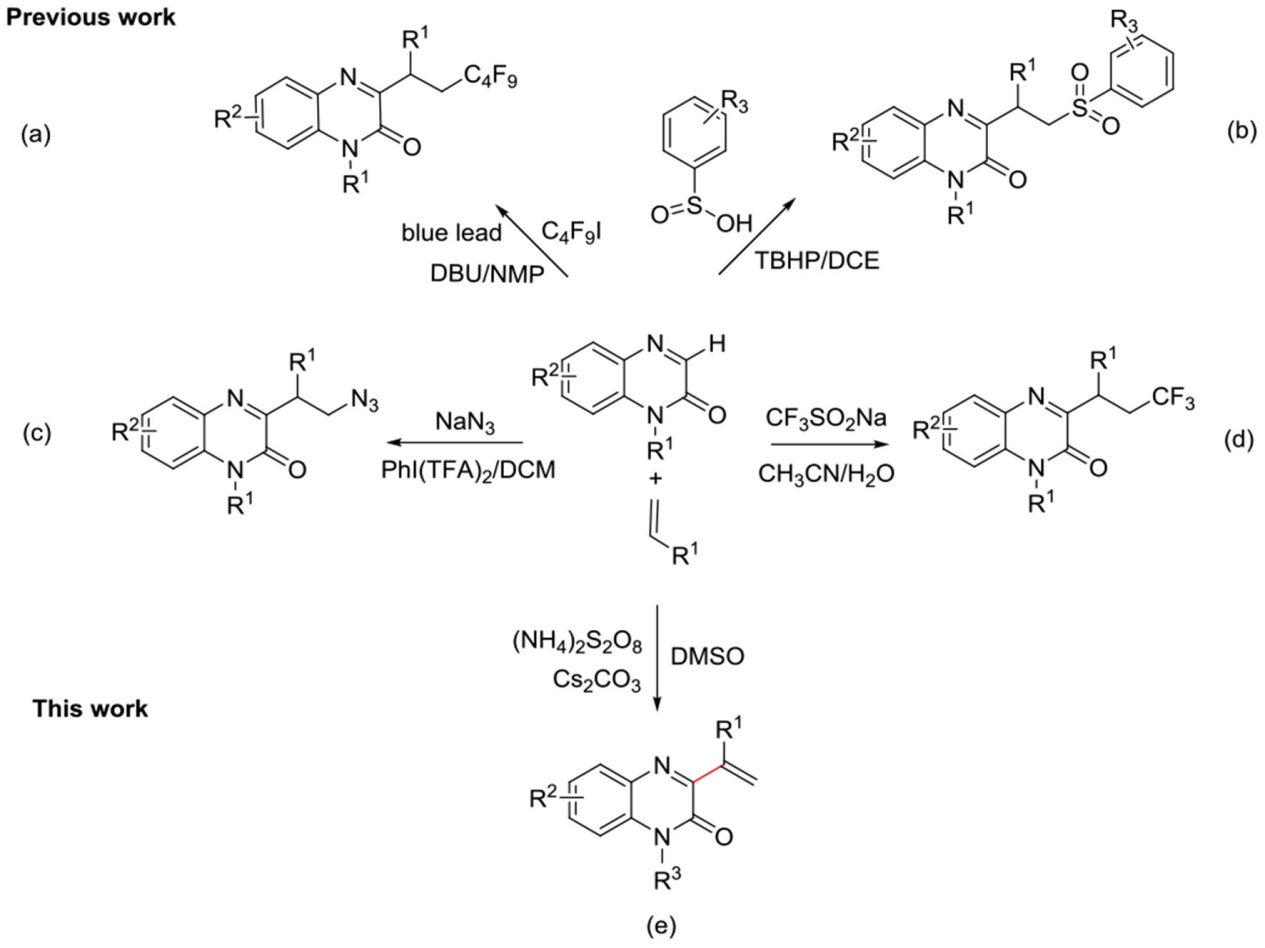
Synthesis of C3 alkyl/vinyl substituted quinoxalin-2(1*H*)-ones.

The Armido Studer' group through a visible-light-initiated to synthesize α-perfluoroalkyl-β-heteroarylation of various alkenes with perfluoroalkyl iodides and quinoxalin-2(1*H*)-ones (Scheme 1A) (Zheng and Studer, 2019). The Dipankar Koley' group explored a strategy to synthesize α-sulfono-β-heteroaryl scaffolds using alkenes with aryl sulfinic acids and quinoxalin-2(1*H*)-ones (Scheme 1B) (Sekhar Dutta et al., 2019). Afterwards, Pengfei Zhang' group reported a hypervalent Iodine(III)-promoted rapid cascade reaction of quinoxalinones with unactivated alkenes and TMSN_3_ (Scheme 1C) (Shen et al., 2019). Recently, Wei Wei' group synthesize 3-trifluoroalkylated quinoxalin-2(1*H*)-ones via K_2_S_2_O_8_-mediated unactivated alkenes with quinoxalin-2(1*H*)-ones and CF_3_SO_2_Na (Scheme 1D) (Meng et al., 2020). To the best of our knowledge, the CDC reaction has not been utilized yet to synthesize quinoxalin-2(1*H*)-one derivatives bearing alkene substituents at the C3 position.

Herein, we report the C-H functionalization of quinoxalin-2(1*H*)-one, in the presence of alkenes, for the direct synthesis of 3-vinylated quinoxalin-2(1*H*)-ones. In the absence of metal/ligand, the reaction was oxidized with ammonium persulfate [(NH_4_)_2_S_2_O_8_] to obtain the target products (Scheme 1E).

## Results and Discussion

When the reaction was carried out with 1-methylquinoxalin-2(1*H*)-one **1a** and styrene **2a** as the-substrates, in the presence of PIFA (oxidant), in DMSO, the desired product was not obtained. Different oxidants, such as PhI(OAc)_2_, TBHP, TBDP, (NH_4_)_2_S_2_O_8_ and K_2_S_2_O_8_ were screened for the reaction. (NH_4_)_2_S_2_O_8_ proved to be the best oxidizing agent, and the final compound was obtained in 45% yield when (NH_4_)_2_S_2_O_8_ was used for oxidation (Table 1, entries 2–6). The target compound was not obtained when the reaction was carried out in the absence of the oxidant (Table 1, entry 7). The reaction was carried out in different solvents such as toluene, EtOAc, acetone, H_2_O, DMF, and CH_3_CN to determine the optimal reaction solvent (Table 1, entries 8–13). The reactions did not proceed smoothly when these solvents were used as the reaction solvents, and the desired products were obtained in significantly low yields. Following this, the effects of different additives, such as CuBr and CuSO_4_, on the product yields were investigated. It was observed that, in the presence of these additives, the products were produced in significantly low yields (Table 1, entries 14, 15). The reaction condition was also optimized with respect to bases to obtain better yields of the products (Table 1, entries 16–20). The experiments revealed that Cs_2_CO_3_ was the most effective in promoting the reactions. Significantly low product yields were obtained when other bases (such as TEA, K_2_CO_3_, NaOH, and NaH) were used to drive the reactions. Following this, the effect of temperature on the product yields was also investigated. When the reaction was carried out at higher or lower temperatures, a decrease in the yield of **3a** was observed (Table 1, entries 21–23). Thus, the reaction conditions were optimized and the maximum yield of the product was obtained when the reaction was carried out in DMSO (0.1 M) with **1a** (0.25 mmol) and **2a** (0.75 mmol) as the substrates, in the presence of (NH_4_)_2_S_2_O_8_ as the oxidant (1 mmol) and Cs_2_CO_3_ (0.75 mmol) as the base, under atmospheric conditions at 80°C for 10 h.

**Table 1:** Screening of reaction conditions^a^.

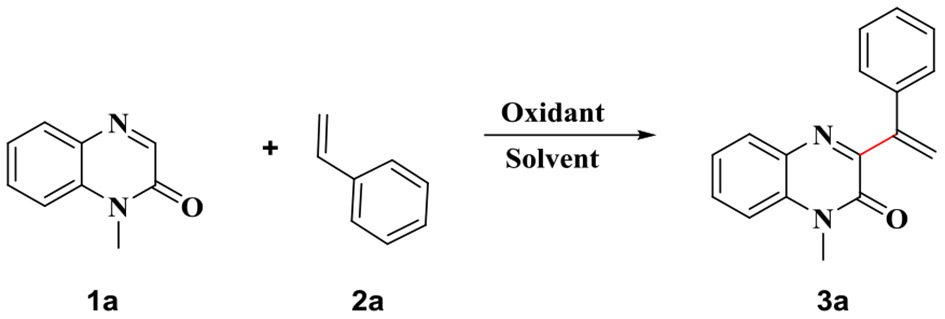
**Entry**	**Oxidant(equiv.)**	**Additives**	**Solvent**	**Yield(%)^[b]^**
1	PIFA		DMSO	0
2	PhI(OAc)_2_		DMSO	0
3	TBHP		DMSO	0
4	TBDP		DMSO	0
5	(NH_4_)_2_S_2_O_8_		DMSO	45
6	K_2_S_2_O_8_		DMSO	42
7	–		DMSO	0
8	(NH_4_)_2_S_2_O_8_		Toluene	0
9	(NH_4_)_2_S_2_O_8_		EtOAc	Trace
10	(NH_4_)_2_S_2_O_8_		Acetone	Trace
11	(NH_4_)_2_S_2_O_8_		H2O	n.d.
12	(NH_4_)_2_S_2_O_8_		DMF	10
13	(NH_4_)_2_S_2_O_8_		CH_3_CN	0
14	(NH_4_)_2_S_2_O_8_	CuBr	DMSO	33
15	(NH_4_)_2_S_2_O_8_	CuSO_4_	DMSO	35
16	(NH_4_)_2_S_2_O_8_	TEA	DMSO	30
17	(NH_4_)_2_S_2_O_8_	K_2_CO_3_	DMSO	56
18	(NH_4_)_2_S_2_O_8_	Cs_2_CO_3_	DMSO	65
19	(NH_4_)_2_S_2_O_8_	NaOH	DMSO	11
20	(NH_4_)_2_S_2_O_8_	NaH	DMSO	Trace
21^c^	(NH_4_)_2_S_2_O_8_	Cs_2_CO_3_	DMSO	42
22^d^	(NH_4_)_2_S_2_O_8_	Cs_2_CO_3_	DMSO	50
23^e^	(NH_4_)_2_S_2_O_8_	Cs_2_CO_3_	DMSO	0

a *Reaction conditions: **1a** (0.25 mmol), **2a** (0.75 mmol), Oxidant (1 mmol), base (0.75 mmol), and solvent at 80°C for 10 h under air*.

b *Isolated yield*.

c *100°C*.

d *60°C*.

e *25°C*.

**Scheme 2:**
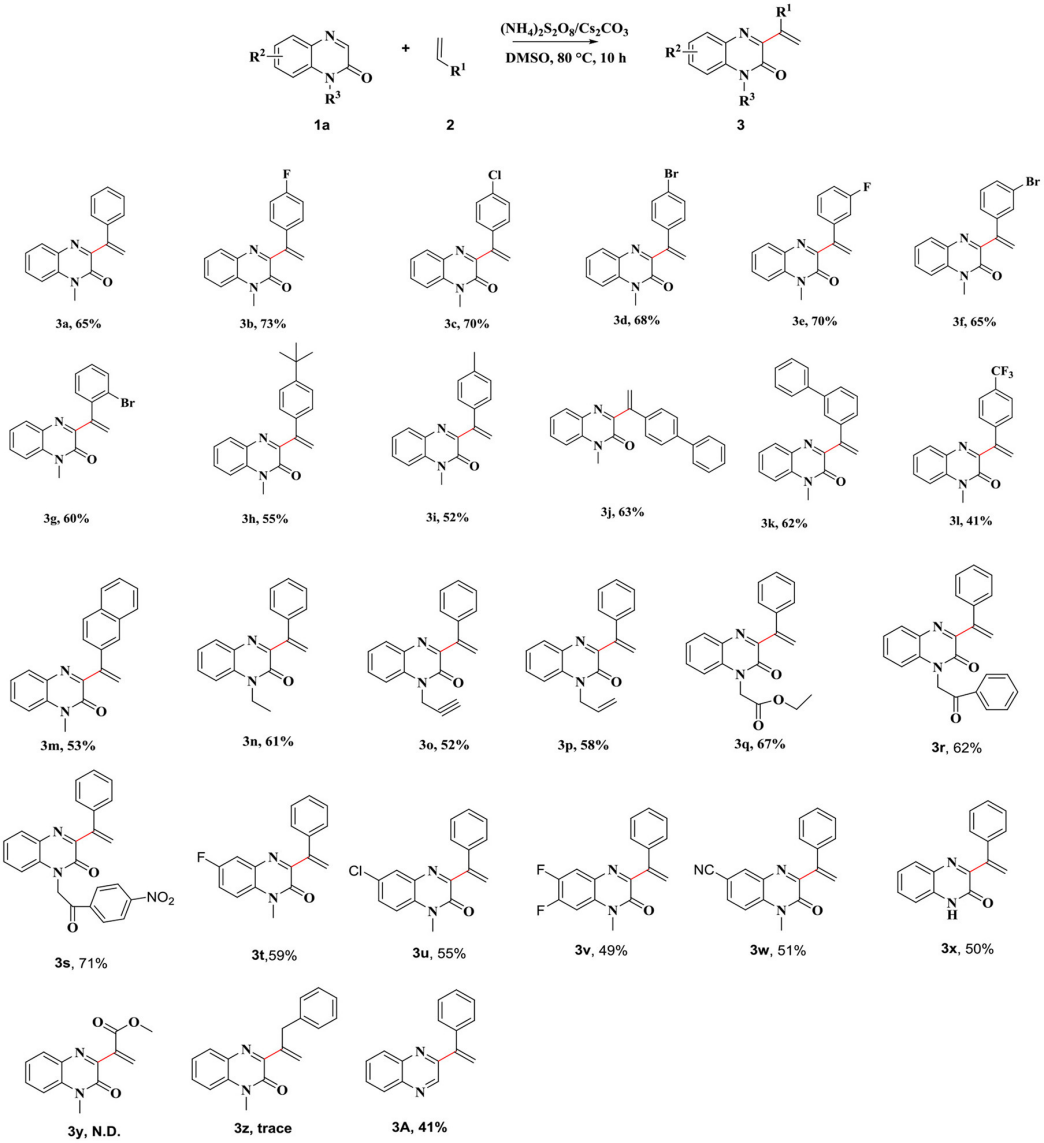
Coupling Reaction of Quinoxalin-2(1*H*)-ones and Alkenes. Reaction conditions: **1a** (0.25 mmol), **2a** (0.75 mmol), Oxidant (1 mmol), base (0.75 mmol), and solvent at 80°C for 10 h under air.

After determining the optimal reaction conditions, we focused on expanding the scope of the reaction. We used various substituted aryl olefins as the substrates to carry out the reactions under the optimal reaction conditions (Scheme 2). The results showed that good functional group tolerance could be achieved under the optimized reaction conditions. A series of olefins bearing electron-withdrawing (4-F, 4-Cl, 4-Br, 3-F, 3-Br, and 2-Br) and electron-donating [4-C(CH_3_)_3_, 4- Me, and 4-Ph] substituents, with groups attached to the phenyl ring, proved to be good substrates for this reaction. The corresponding 3-vinylated quinoxalin-2(1*H*)-ones were produced in moderate yields (**3b**–**3p**). The product yields decreased when substrates bearing electron donating groups were used for carrying out the reactions. The product yield was dictated by the strength of the electron donating groups. We also observed that the reaction proceeded smoothly when a strong electron-withdrawing group (4-CF_3_) was present on the phenyl ring. The corresponding product (**3l)** was obtained in 41% yield. We replaced different heterocyclic rings and investigated the effect of such a change on the yields of the products. The target product **3m** was obtained when the ring of choice was naphthalene. Subsequently, we also investigated the influence of quinoxalin-2(1*H*)-ones, bearing different substituents, on the applicability of the reaction (Scheme 2). The results revealed that the applicability of the method was extensive. The reactions were carried out with derivatives of quinoxalin-2(1*H*)-one derivatives with different *N*-substituted groups, such as *N*-ethyl, *N*-pentyl, *N*-vinyl, *N*-ethynyl, *N*-esteryl, *N*-(2-oxo-2-phenylethyl), and *N*-[2-oxo-2-(4-nitrophenyl)], could generate target compounds **3m**–**3s** in moderate to good yields. The reactions progressed smoothly when the reactions were carried out with the quinoxalin-2(1*H*)-benzene ring, bearing halogen atoms at different positions, and the desired products in moderate yields (**3t**–**3v**). It is worth mentioning that the corresponding target compound **3w** was obtained in 51% yield, even when a relatively strong electron-withdrawing group was present on the benzene ring. The target compound **3x** was obtained in 50% yield, when the unsubstituted quinoxalin-2(1*H*)-one was used as the substrate. Regrettably, when replacing styrene with methyl acrylate and allylbenzene, the corresponding product **3y** and 3z were not obtained. Surprisingly, by replacing the substrate with quinoxaline, the corresponding product **3A** can be obtained in 41% yield.

**Scheme 3:**
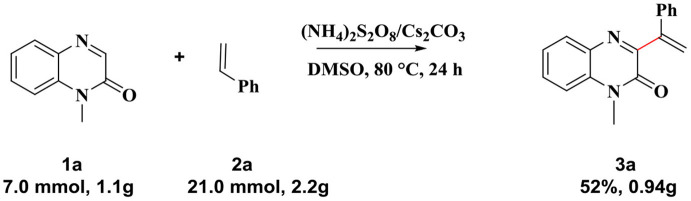
Large scale experiment: **1a** (7.0 mmol), **2a** (21.0 mmol), (NH_4_)_2_S_2_O_8_(19.6 mmol), Cs_2_CO_3_ (10.3 mmol) in 20 mL of DMSO, 80°C, 24 h. Product **3a** was isolated in 52% yield.

Encouraged by this reaction and sustainable synthesis, we conducted scale-up experiments to investigate the synthetic utility of the reaction. When 7.0 mmol of 1-methylquinoxalin-2(1*H*)-one **1a** was treated with 21.0 mmol styrene (**2a**), the corresponding product **3a** was obtained with a yield of 52%, although an extended reaction time was required (Scheme 3).

**Scheme 4:**
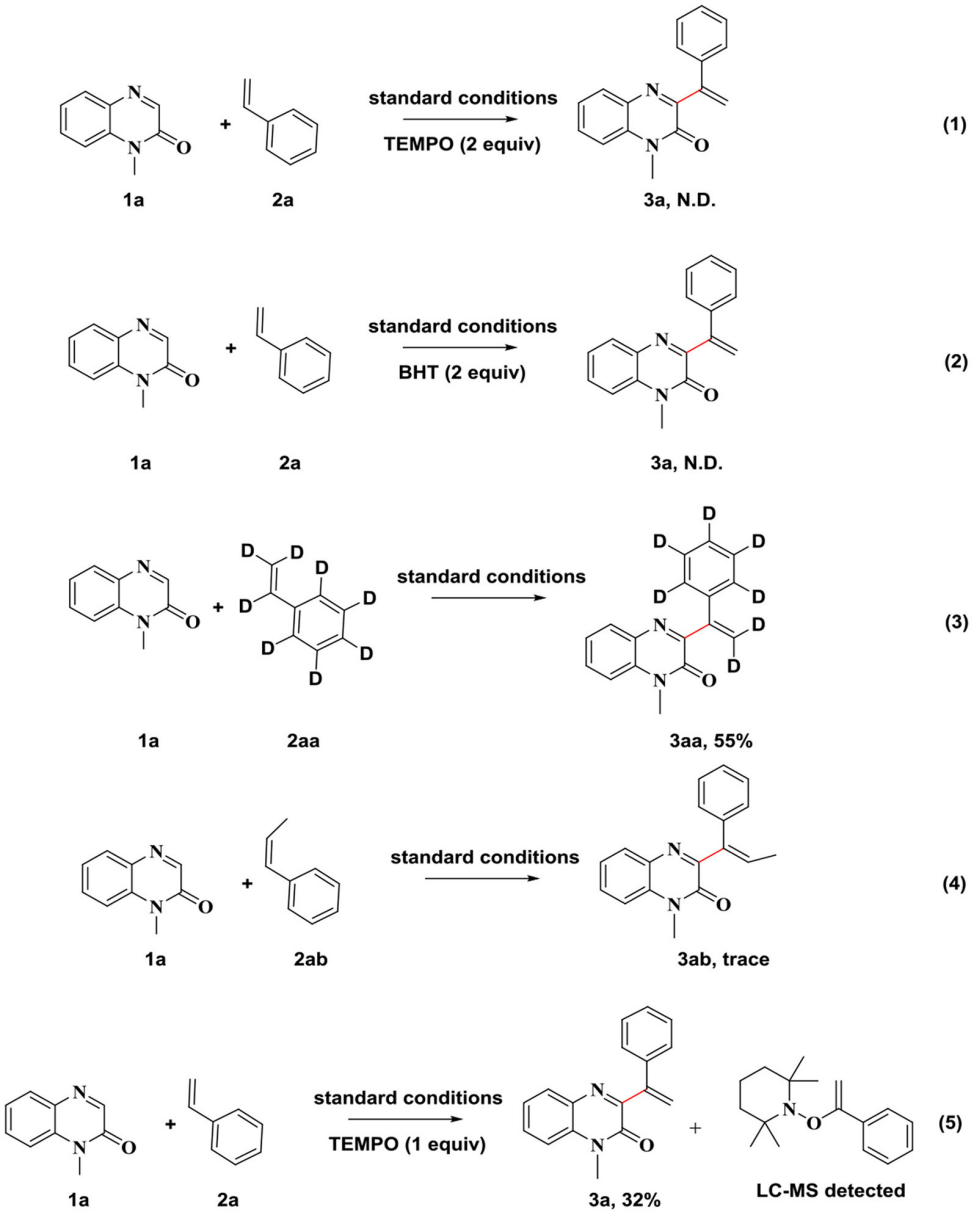
Controlled experiments.

Control reactions were carried out to investigate the reaction mechanism. The introduction of a radical inhibitor (TEMPO or BHT) into the model reaction mixture, significantly inhibited the progress of the reaction. The corresponding 3-vinylated quinoxalin-2(1*H*)-ones was not obtained (Scheme 4, Equations 1, 2). This, indicated that the reaction proceeded through a radical mechanism. The reaction proceeded smoothly in the presence of deuterated styrene, producing the corresponding target compound (Scheme 4, Equation 3), which indicated that the hydrogen of the terminal double bond in the substrate was not involved in the reaction process. If styrene was replaced with β-methylstyrene, the target product couldn't be obtained under standard conditions, and it was probably due to the spatial site resistance that the reaction did not proceed (Scheme 4, Equation 4). When the amount of TMPEO was reduced, the captured intermediate structure was detected by liquid-phase mass spectrometry, indicating that the reaction mechanism may have gone through this process (Scheme 4, Equation 5).

**Scheme 5:**
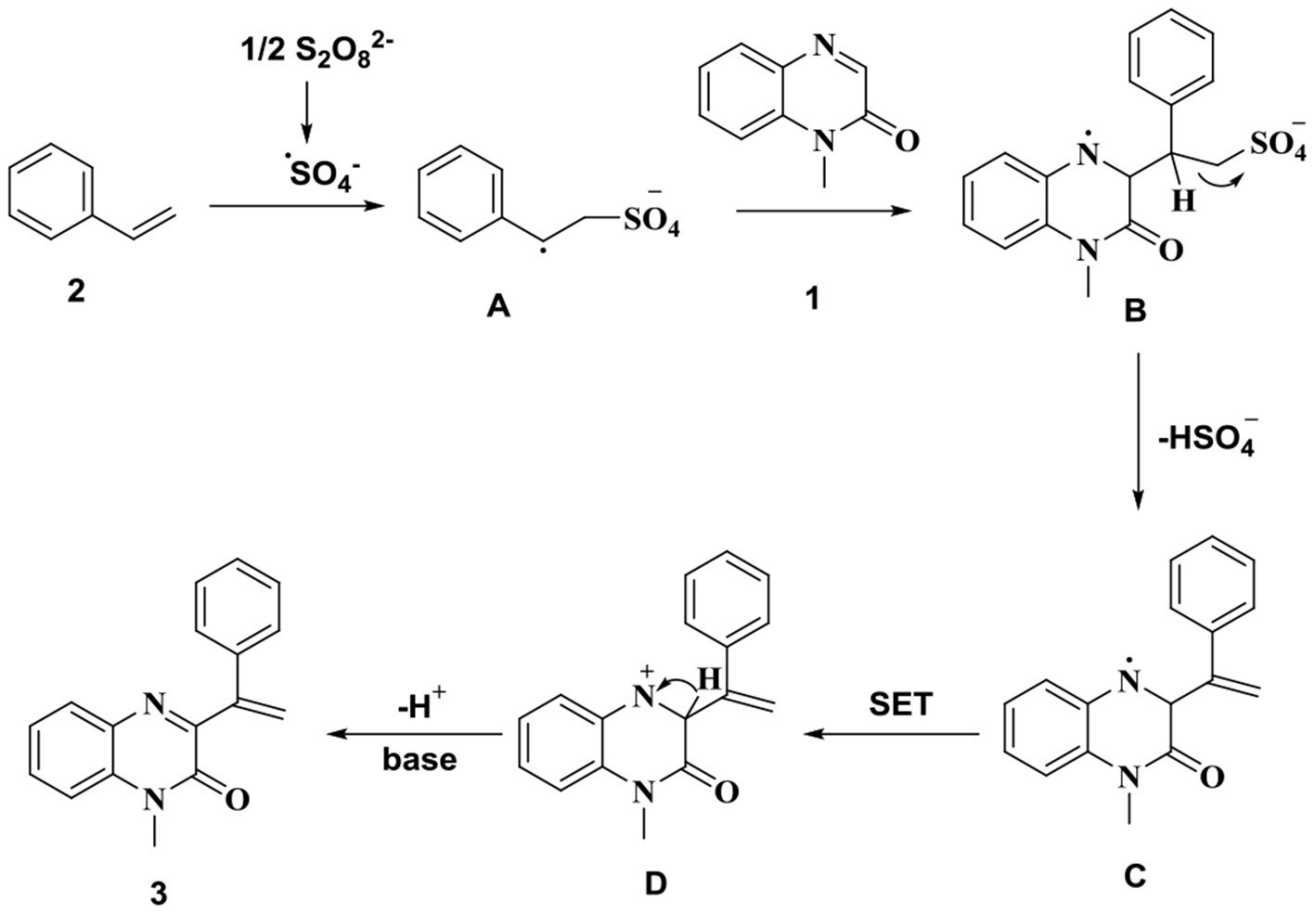
Proposed mechanism.

We proposed the possible reaction mechanism based on these observations and the results presented in literature reports (Bag and Maiti, 2016; Gupta et al., 2017; Fu et al., 2018; Toonchue et al., 2018; Wei et al., 2018; Jin et al., 2019; Sekhar Dutta et al., 2019; Shen et al., 2019; Xie et al., 2019a,b; Zheng and Studer, 2019; Meng et al., 2020; Shi and Wei, 2020; Xie et al., 2020; Ali et al., 2021) (Scheme 5). Initially, alkene **2** reacts with sulfate radical anion (generated *in situ*) to generate the alkyl radical **A**. The addition of alkyl radical **A** to quinoxalin-2(1*H*)-one **1** produces the nitrogen radical **B**. The intermediate radical **B** generates free radical **C** under heating conditions. The bisulfate anion is released during the process. Single-electron transfer (SET), in the presence of S_2_${\text{O}}_{8}^{2-}$, produces the nitrogen cation intermediate **D** from the radical **C**. Finally, the intermediate **D** is deprotonated under base conditions to produce the final product **3**.

## Conclusion

In summary, we have reported a simple and efficient C3-H vinylation reaction with quinoxalin-2(1*H*)-one as the substrate, in the presence of alkenes and absence of metals. A series of 3-vinylated quinoxalin-2(1*H*)-ones with potential biological activities can be obtained when the reactions are carried out in the presence of (NH_4_)_2_S_2_O_8_. Further research to determine the applicability of the synthetic procedure is presently underway in our laboratory.

## Data Availability Statement

The original contributions presented in the study are included in the article/Supplementary Material, further inquiries can be directed to the corresponding author/s.

## Author Contributions

YLv and JH were responsible for designing the experiments. RD, YLi, and YC performed the experimentations. JH, YLv, and JY analyzed the results and wrote the publication. All authors contributed to the article and approved the submitted version.

## Conflict of Interest

The authors declare that the research was conducted in the absence of any commercial or financial relationships that could be construed as a potential conflict of interest.
