# Supplementary material for: Metal-Free Direct C–H Functionalization of Quinoxalin-2(1H)-Ones to Produce 3-Vinylated Quinoxalin-2(1H)-Ones in the Presence of Alkenes
Source: Front Chem. 2021 Apr 30;9:672051. doi: 10.3389/fchem.2021.672051 (PMC8119786; doi:10.3389/fchem.2021.672051)
Supplement: Supplementary file 1 [file Data_Sheet_1.DOCX]

Supplementary Material

[**Metal-free Direct C–H Functionalization of Quinoxalin-2(1*H*)-ones to Produce 3-Vinylated Quinoxalin-2(1*H*)-ones in the Presence of Alkenes**](https://scifinder.cas.org/scifinder/view/reaction/javascript:;)

Rongcai Ding, Yingxue Li, Yaoyao Chang, Yue Liu, Jie Yu, Yanna Lv*^,^*^a^* , Jinxing

Hu**^,^*^a^*

*^a^* School of Pharmacy, Weifang Medical University, Weifang, 261053 P.R. China.

Corresponding Author:

* E-mail: [lynlyna@163.com](mailto:lynlyna@163.com) *,* jinxinghu2013@wfmc.edu.cn

**Table of Contents**

**I.** General Experimental Details……………………………………………............................2

**II.** Spectral Data of Products..................................................................................................3-11

**III**. Copies of ^1^H NMR and ^13^C NMR Spectra.....................................................,,..............12-39

**Ⅳ.** References..........................................................................................................................39

**I.General Experimental Details**

1. ***Synthesis of substrates***

The substrates quinoxalin-2(1*H*)-ones were synthesized according to modified previous reported protocols^[1-4]^.

***(2)*** ***General methods (3a as example).***

1-methylquinoxalin-2(1*H*)-one **1a** (40 mg, 0.25 mmol), styrene (78 mg, 0.75 mmol, 3.0 equiv.), caesium carbonate (175 mg, 0.75 mmol, 3.0 equiv.) and (NH_4_)_2_S_2_O_8_ (142.6 mg, 1 mmol) were added consecutively in DMSO under room temperature (3 mL). The resulting mixture was stirred at 80°C for 10 h until the starting material was completely consumed. After the reaction was finished, water (10 mL) was added and the solution was extracted with DCM. Then evaporation of the solvent, the mixture was purified by silica gel column chromatography (PE/EA=4:1) to afford the desired products **3a**.

**Ⅱ. Spectral Data of Products**

***1-methyl-3-(1-phenylvinyl)quinoxalin-2(1H)-one* (3a)**

Yield: 65%, Yellow solid, m.p.169.6-170.5°C. ^1^H NMR (400 MHz, DMSO-*d*_6_) δ 7.82 (d, *J* = 7.8 Hz, 1H), 7.72 – 7.55 (m, 2H), 7.37 (m, 6H), 5.95 (s, 1H), 5.84 (s, 1H), 3.63 (s, 3H). ^13^C NMR (100 MHz, DMSO) δ 157.21, 154.06, 145.95, 139.34, 134.02, 132.52, 131.24, 130.05, 128.75, 128.14, 126.92, 123.94, 119.94, 115.24, 29.57. HRMS (ESI-Q-TOF, m/z) calcd for C_17_H_14_N_2_O [M+H]^+^: 263.1106, found [M+H]^+^: 263.1163.

***3-(1-(4-fluorophenyl)vinyl)-1-methylquinoxalin-2(1H)-one* (3b)**

Yield: 73%, Yellow solid, m.p.127.8-128.5°C. ^1^H NMR (400 MHz, DMSO-*d*_6_) δ 7.81 (d, *J* = 7.7 Hz, 1H), 7.72 – 7.55 (m, 2H), 7.41 (d, *J* = 7.1 Hz, 3H), 7.16 (t, *J* = 8.1 Hz, 2H), 5.91 (d, *J* = 20.7 Hz, 2H), 3.63 (s, 3H). ^13^C NMR (100 MHz, DMSO) δ 163.45, 161.02, 156.75, 154.05, 144.89, 135.90, 134.03, 132.51, 131.29, 130.07, 129.15, 129.07, 123.95, 120.31, 115.62, 115.40, 115.23, 29.58. ^19^F NMR (377 MHz, DMSO-*d*_6_) δ -114.75..HRMS (ESI-Q-TOF, m/z) calcd for C_17_H_13_FN_2_O [M+H]^+^: 281.1012, found [M+H]^+^: 281.1060.

***3-(1-(4-chlorophenyl)vinyl)-1-methylquinoxalin-2(1H)-one*（3c）**

Yield: 70%, Yellow solid, m.p.141.1-142.1°C. ^1^H NMR (400 MHz, DMSO-*d*_6_) δ 7.81 (d, *J* = 7.9 Hz, 1H), 7.68 (t, *J* = 7.6 Hz, 1H), 7.60 (d, *J* = 8.3 Hz, 1H), 7.41 (d, *J* = 7.6 Hz, 5H), 5.98 (s, 1H), 5.93 (s, 1H), 3.63 (s, 3H). ^13^C NMR (100 MHz, DMSO) δ 156.50, 154.03, 144.84, 138.33, 134.02, 132.74, 132.51, 131.36, 130.07, 128.90, 128.70, 123.98, 121.05, 115.24, 29.59. HRMS (ESI-Q-TOF, m/z) calcd for C_17_H_13_ClN_2_O [M+H]^+^: 297.0716, found [M+H]^+^: 297.0780.

***3-(1-(4-bromophenyl)vinyl)-1-methylquinoxalin-2(1H)-one* (3d)**

Yield: 68%, Yellow solid, m.p.152.7-153.5°C. ^1^H NMR (400 MHz, DMSO-*d*_6_) δ 7.82 (d, *J* = 8.0 Hz, 1H), 7.68 (t, *J* = 7.8 Hz, 1H), 7.60 (d, *J* = 8.4 Hz, 1H), 7.52 (d, *J* = 8.3 Hz, 2H), 7.40 (t, *J* = 7.6 Hz, 1H), 7.32 (d, *J* = 8.2 Hz, 2H), 5.96 (d, *J* = 18.6 Hz, 2H), 3.62 (s, 3H). ^13^C NMR (100 MHz, DMSO) δ 156.46, 154.03, 144.94, 138.72, 134.04, 132.53, 131.62, 131.36, 130.10, 129.23, 123.98, 121.35, 121.09, 115.25, 29.59. HRMS (ESI-Q-TOF, m/z) calcd for C_17_H_13_BrN_2_O [M+H]^+^: 341.0211, 342.0191 found [M+H]^+^: 341.0263, 342.0243.

***3-(1-(3-fluorophenyl)vinyl)-1-methylquinoxalin-2(1H)-one* (3e)**

Yield: 70%, Yellow solid, m.p.136.5-137.4°C. ^1^H NMR (400 MHz, DMSO-*d*_6_) δ 7.82 (d, *J* = 7.9 Hz, 1H), 7.68 (t, *J* = 7.8 Hz, 1H), 7.60 (d, *J* = 8.4 Hz, 1H), 7.38 (dt, *J* = 14.8, 7.8 Hz, 2H), 7.27 – 7.09 (m, 3H), 6.03 (s, 1H), 5.94 (s, 1H), 3.63 (s, 3H). ^13^C NMR (100 MHz, DMSO) δ 163.86, 161.45, 156.49, 154.06, 144.85, 141.92, 141.84, 134.09, 132.54, 131.34, 130.65, 130.57, 130.09, 123.95, 123.22, 121.54, 115.24, 114.96, 114.75, 113.91, 113.69, 29.60. ^19^F NMR (377 MHz, DMSO-*d*_6_) δ -113.64. HRMS (ESI-Q-TOF, m/z) calcd for C_17_H_13_FN_2_O [M+H]^+^ : 281.1012, found [M+H]^+^: 281.1062.

***3-(1-(3-bromophenyl)vinyl)-1-methylquinoxalin-2(1H)-one* (3f)**

Yield: 65%, Yellow solid, m.p.101.4-102.5°C. ^1^H NMR (400 MHz, ) δ 7.81 (d, J = 7.8 Hz, 1H), 7.71 – 7.65 (m, 1H), 7.59 (d, J = 12.5 Hz, 2H), 7.51 (d, J = 7.6 Hz, 1H), 7.40 (t, J = 7.5 Hz, 1H), 7.31 (dd, J = 15.0, 7.4 Hz, 2H), 6.01 (s, 1H), 5.97 (s, 1H), 3.63 (s, 3H). ^13^C NMR (100 MHz, ) δ 156.18, 153.96, 144.56, 141.88, 133.99, 132.45, 131.28, 130.77, 130.00, 129.54, 126.18, 123.89, 122.04, 121.90, 115.17, 29.51.HRMS (ESI-Q-TOF, m/z) calcd for C_17_H_13_BrN_2_O [M+H]^+^: 341.0211, 343.0191 found [M+H]^+^: 341.0275, 343.0232.

***3-(1-(2-bromophenyl)vinyl)-1-methylquinoxalin-2(1H)-one* (3g)**

Yield: 60%, Light yellow solid, m.p.105.0-106.1°C. ^1^H NMR (400 MHz, DMSO-*d*_6_) δ 7.67 – 7.55 (m, 4H), 7.44 (d, *J* = 4.4 Hz, 2H), 7.36 – 7.25 (m, 2H), 6.76 (s, 1H), 5.86 (s, 1H), 3.66 (s, 3H). ^13^C NMR (100 MHz, DMSO) δ 153.91, 153.67, 145.93, 142.54, 133.51, 132.62, 132.35, 131.89, 131.29, 130.06, 129.69, 128.20, 126.91, 124.00, 122.37, 115.18, 29.62. HRMS (ESI-Q-TOF, m/z) calcd for C_17_H_13_BrN_2_O [M+H]^+^: 341.0211,343.0191 found [M+H]^+^: 341.0250, 343.0240.

***3-(1-(4-(tert-butyl)phenyl)vinyl)-1-methylquinoxalin-2(1H)-one* (3h)**

Yield: 55%, Yellow solid, m.p.79.6-80.5°C. ^1^H NMR (400 MHz, DMSO-*d*_6_) δ 7.83 (d, *J* = 7.9 Hz, 1H), 7.67 (t, *J* = 7.7 Hz, 1H), 7.59 (d, *J* = 8.3 Hz, 1H), 7.38 (dd, *J* = 23.9, 8.0 Hz, 3H), 7.27 (d, *J* = 8.3 Hz, 2H), 5.90 (s, 1H), 5.76 (s, 1H), 3.62 (s, 3H), 1.28 (s, 9H). ^13^C NMR (100 MHz, DMSO) δ 157.42, 154.04, 150.54, 145.72, 136.45, 134.01, 132.53, 131.20, 130.04, 126.58, 125.52, 123.93, 119.09, 115.22, 34.73, 31.55, 29.55. HRMS (ESI-Q-TOF, m/z) calcd for C_21_H_22_N_2_O [M+H]^+^: 319.1732, found [M+H]^+^: 319.1786.

***1-methyl-3-(1-(p-tolyl)vinyl)quinoxalin-2(1H)-one* (3i)**

Yield: 52%, Yellow solid, m.p. 104.6-105.5°C. ^1^H NMR (400 MHz, DMSO-*d*_6_) δ 7.82 (d, *J* = 7.0 Hz, 1H), 7.62 (dd, *J* = 29.5, 7.0 Hz, 2H), 7.44 – 7.35 (m, 1H), 7.18 (dd, *J* = 36.3, 5.9 Hz, 4H), 5.82 (d, *J* = 57.4 Hz, 2H), 3.62 (s, 3H), 2.30 (s, 3H). ^13^C NMR (100 MHz, DMSO) δ 157.41, 154.04, 145.80, 137.44, 136.50, 134.01, 132.52, 131.18, 130.02, 129.32, 126.80, 123.91, 118.90, 115.20, 29.54, 21.21.HRMS (ESI-Q-TOF, m/z) calcd for C_18_H_16_N_2_O_,_ [M+H]^+^: 277.1263, found [M+H]^+^: 277.1220.

***3-(1-([1,1'-biphenyl]-4-yl)vinyl)-1-methylquinoxalin-2(1H)-one*（3j）**

Yield: 63%, Yellow solid, m.p.72.4-73.5°C. ^1^H NMR (400 MHz, DMSO-*d*_6_) δ 7.81 (d, *J* = 7.9 Hz, 1H), 7.68 (t, *J* = 10.4 Hz, 2H), 7.62 – 7.24 (m, 10H), 5.99 (d, *J* = 15.6 Hz, 2H), 3.63 (s, 3H).^13^C NMR (100 MHz, DMSO) δ 156.27, 154.05, 144.67, 142.00, 134.09, 132.55, 131.36, 130.86, 130.10, 129.66, 126.29, 123.96, 122.14, 122.01, 115.26, 29.60. HRMS (ESI-Q-TOF, m/z) calcd for C_23_H_18_N_2_O [M+H]^+^: 339.1419, found [M+H]^+^: 339.1482.

***3-(1-([1,1'-biphenyl]-3-yl)vinyl)-1-methylquinoxalin-2(1H)-one* (3k)**

Yield: 62%, Yellow solid, m.p.74.2-75.1 °C. ^1^H NMR (400 MHz, DMSO-*d*_6_) δ 7.85 (d, *J* = 7.9 Hz, 1H), 7.66 (dq, *J* = 19.5, 11.2, 8.7 Hz, 6H), 7.43 (tt, *J* = 17.4, 7.7 Hz, 6H), 6.01 (s, 1H), 5.85 (s, 1H), 3.64 (s, 3H). ^13^C NMR (100 MHz, DMSO) δ 157.20, 154.09, 145.52, 140.17, 139.88, 138.42, 134.07, 132.57, 131.28, 130.08, 129.44, 127.98, 127.51, 127.05, 123.97, 119.92, 115.27, 29.59. HRMS (ESI-Q-TOF, m/z) calcd for C_23_H_18_N_2_O [M+H]^+^: 339.1419, found [M+H]^+^: 339.1466.

***1-methyl-3-(1-(4-(trifluoromethyl)phenyl)vinyl)quinoxalin-2(1H)-one*（3l）**

Yield: 41%, Yellow solid, m.p.98.3-99.2°C. ^1^H NMR (400 MHz, DMSO-*d*_6_) δ 7.82 (d, *J* = 7.9 Hz, 1H), 7.73 – 7.55 (m, 6H), 7.41 (t, *J* = 7.5 Hz, 1H), 6.08 (d, *J* = 4.3 Hz, 2H), 3.63 (s, 3H).^13^C NMR (101 MHz, DMSO) δ 156.12, 154.03, 144.95, 143.66, 134.07, 132.55, 131.44, 130.14, 127.92, 125.63, 125.59, 124.02, 122.89, 115.28, 29.60. ^19^F NMR (377 MHz, DMSO-*d*_6_) δ -60.90. HRMS (ESI-Q-TOF, m/z) calcd for C_18_H_13_F_3_N_2_O [M+H]^+^: 331.0980, found [M+H]^+^: 331.0926.

***1-methyl-3-(1-(naphthalen-2-yl)vinyl)quinoxalin-2(1H)-one*（3m）**

Yield: 53%, Yellow solid, m.p.92.2-93.1°C. ^1^H NMR (400 MHz, DMSO-*d*_6_) δ 7.93 – 7.79 (m, 5H), 7.72 – 7.66 (m, 1H), 7.64 – 7.59 (m, 2H), 7.49 (dq, *J* = 10.7, 6.9, 6.0 Hz, 2H), 7.41 (t, *J* = 7.5 Hz, 1H), 6.09 (s, 1H), 5.93 (s, 1H), 3.64 (s, 3H). ^13^C NMR (100 MHz, DMSO) δ 157.27, 154.13, 146.05, 136.87, 134.13, 133.39, 132.91, 132.64, 131.28, 130.11, 128.58, 128.18, 127.88, 126.68, 126.57, 125.67, 125.35, 123.96, 120.50, 115.27, 29.59.HRMS (ESI-Q-TOF, m/z) calcd for C_21_H_16_N_2_O [M+H]^+^: 313.1263, found [M+H]^+^: 313.1222.

***1-ethyl-3-(1-phenylvinyl)quinoxalin-2(1H)-one*（3n）**

Yield: 61%, Yellow solid, m.p.87.6-88.5°C. ^1^H NMR (400 MHz, DMSO-*d*_6_) δ 7.83 (d, *J* = 7.9 Hz, 1H), 7.67 (d, *J* = 5.9 Hz, 2H), 7.42 – 7.37 (m, 1H), 7.33 (d, *J* = 8.7 Hz, 5H), 5.95 (s, 1H), 5.88 (s, 1H), 4.26 (q, *J* = 7.0 Hz, 2H), 1.24 (t, *J* = 7.0 Hz, 3H). ^13^C NMR (100 MHz, DMSO) δ156.53, 152.98, 145.34, 138.85, 132.23, 130.78, 129.82, 128.18, 127.55, 126.37, 123.29, 119.60, 114.35, 36.83, 12.23.HRMS (ESI-Q-TOF, m/z) calcd for C_18_H_16_N_2_O [M+H]^+^: 277.1263, found [M+H]^+^: 277.1220.

***3-(1-phenylvinyl)-1-(prop-2-yn-1-yl)quinoxalin-2(1H)-one* (3o)**

Yield: 52%, Yellow solid, m.p.146.6-147.5°C. ^1^H NMR (400 MHz, DMSO-*d*_6_) δ 7.85 (d, *J* = 7.6 Hz, 1H), 7.68 (dd, *J* = 24.8, 7.3 Hz, 2H), 7.39 (d, *J* = 36.8 Hz, 6H), 5.93 (d, *J* = 28.2 Hz, 2H), 5.08 (s, 2H), 3.24 (d, *J* = 14.7 Hz, 1H). ^13^C NMR (100 MHz, DMSO) δ 157.04, 153.15, 145.66, 139.25, 132.70, 132.36, 131.38, 130.32, 128.80, 128.22, 126.99, 120.57, 115.45, 78.49, 75.77, 31.83. HRMS (ESI-Q-TOF, m/z) calcd for C_19_H_14_N_2_O [M+H]^+^: 287.1106, found [M+H]^+^: 287.1146.

***1-allyl-3-(1-phenylvinyl)quinoxalin-2(1H)-one* (3p)**

Yield: 58%, Yellow liquid. m.p.97.8-98.9°C. ^1^H NMR (400 MHz, DMSO-*d*_6_) δ 7.83 (d, *J* = 7.9 Hz, 1H), 7.64 (t, *J* = 7.7 Hz, 1H), 7.52 (d, *J* = 8.5 Hz, 1H), 7.43 – 7.29 (m, 6H), 6.00 – 5.86 (m, 3H), 5.19 (d, *J* = 10.4 Hz, 1H), 5.09 (d, *J* = 17.3 Hz, 1H), 4.88 (d, *J* = 4.4 Hz, 2H). ^13^C NMR (100 MHz, DMSO) δ 157.17, 153.66, 145.86, 139.36, 133.06, 132.71, 132.00, 131.19, 130.24, 128.77, 128.16, 126.93, 124.01, 120.23, 117.60, 115.51, 44.36. HRMS (ESI-Q-TOF, m/z) calcd for C_19_H_16_N_2_O [M+H]^+^: 289.1263, found [M+H]^+^: 289.1263.

***ethyl 2-(2-oxo-3-(1-phenylvinyl)quinoxalin-1(2H)-yl)acetate (3q)***

Yield: 67%, Yellow liquid. ^1^H NMR (400 MHz, DMSO-*d*_6_) δ 7.86 (d, *J* = 6.9 Hz, 1H), 7.68 – 7.53 (m, 2H), 7.38 (m, 6H), 5.93 (d, *J* = 28.9 Hz, 2H), 5.10 (s, 2H), 4.16 (q, *J* = 7.1 Hz, 2H), 1.20 (t, *J* = 7.1 Hz, 3H).^13^C NMR (100 MHz, DMSO) δ 167.91, 156.73, 153.86, 145.58, 139.20, 133.28, 132.55, 131.48, 130.35, 128.79, 128.24, 126.94, 124.35, 120.61, 115.07, 61.83, 44.27, 14.46. HRMS (ESI-Q-TOF, m/z) calcd for C_20_H_18_N_2_O_3_ [M+H]^+^: 335.1317, found [M+H]^+^: 335.1362.

***1-(2-oxo-2-phenylethyl)-3-(1-phenylvinyl)quinoxalin-2(1H)-one (3r)***

Yield: 62%, Yellow solid, m.p.81.6-82.5°C. ^1^H NMR (400 MHz, DMSO-d6) δ 8.13 (d, J = 7.7 Hz, 2H), 7.88 (d, J = 7.9 Hz, 1H), 7.75 (t, J = 7.3 Hz, 1H), 7.64 – 7.51 (m, 5H), 7.41 (t, J = 7.5 Hz, 1H), 7.36 – 7.31 (m, 4H), 5.96 (s, 1H), 5.91 (d, J = 5.8 Hz, 3H). ^13^C NMR (101 MHz, DMSO) δ 192.20, 156.15, 153.29, 145.17, 138.69, 134.30, 134.13, 133.15, 132.03, 130.81, 129.70, 128.84, 128.21, 127.63, 126.34, 123.60, 119.93, 114.79, 49.03. HRMS (ESI-Q-TOF, m/z) calcd for C_24_H_18_N_2_O_2_ [M+H]^+^: 367.1368, found [M+H]^+^: 367.1316.

***1-(2-(4-nitrophenyl)-2-oxoethyl)-3-(1-phenylvinyl)quinoxalin-2(1H)-one*（3s）**

Yield: 71%, Yellow solid, m.p.145.0-146.1°C. ^1^H NMR (400 MHz, DMSO-*d*_6_) δ 8.19 (d, *J* = 8.6 Hz, 2H), 7.86 (d, *J* = 7.9 Hz, 1H), 7.55 (d, *J* = 8.3 Hz, 2H), 7.38 (m, 8H), 5.98 (d, *J* = 8.4 Hz, 2H), 5.64 (s, 2H). ^13^C NMR (100 MHz, DMSO) δ156.63, 153.64, 146.70, 145.17, 143.66, 138.81, 132.44, 132.34, 130.80, 129.88, 128.20, 127.94, 127.62, 126.51, 123.75, 123.72, 120.11, 114.74, 44.67. HRMS (ESI-Q-TOF, m/z) calcd for C_24_H_17_N_3_O_4_ [M+H]^+^: 412.1219, found [M+H]^+^: 412.1273.

***6-fluoro-1-methyl-3-(1-phenylvinyl)quinoxalin-2(1H)-one (3t)***

Yield: 59%, Yellow solid, m.p.115.2-116.1°C. ^1^H NMR (400 MHz, DMSO-*d*_6_) δ 7.86 (dd, *J* = 8.8, 6.1 Hz, 1H), 7.50 (dd, *J* = 10.9, 2.5 Hz, 1H), 7.35 – 7.22 (m, 6H), 5.94 (s, 1H), 5.83 (s, 1H), 3.59 (s, 3H).^13^C NMR (100 MHz, DMSO) δ 164.07, 161.62, 155.59, 153.44, 145.17, 138.71, 135.24, 135.11, 131.76, 131.66, 129.00, 128.98, 128.18, 127.59, 126.38, 119.49, 111.19, 110.96, 101.74, 101.46, 29.42. ^19^F NMR (377 MHz, DMSO-*d*_6_) δ -103.87. HRMS (ESI-Q-TOF, m/z) calcd for C_17_H_13_FN_2_O [M+H]^+^: 281.1012, found [M+H]^+^: 281.1040.

***6-chloro-1-methyl-3-(1-phenylvinyl)quinoxalin-2(1H)-one*（3u）**

Yield: 55%, Yellow solid, m.p.153.4-154.3°C. ^1^H NMR (400 MHz, DMSO-*d*_6_) δ 7.81 (d, *J* = 8.5 Hz, 1H), 7.70 (d, *J* = 1.9 Hz, 1H), 7.42 (dd, *J* = 8.6, 1.9 Hz, 1H), 7.33 (d, *J* = 8.0 Hz, 5H), 5.95 (s, 1H), 5.85 (s, 1H), 3.60 (s, 3H).^13^C NMR (100 MHz, DMSO) δ 156.77, 153.33, 145.14, 138.64, 135.08, 134.60, 130.96, 130.74, 128.17, 127.61, 126.40, 123.42, 119.77, 114.50, 29.25.HRMS (ESI-Q-TOF, m/z) calcd for C_17_H_13_ClN_2_O [M+H]^+^: 297.0716, found [M+H]^+^: 297.0772.

***6,7-difluoro-1-methyl-3-(1-phenylvinyl)quinoxalin-2(1H)-one*（3v）**

Yield: 49%, Yellow solid, m.p.127.5-128.3°C. ^1^H NMR (400 MHz, DMSO-*d*_6_) δ 7.91 (t, *J* = 9.4 Hz, 1H), 7.77 (dd, *J* = 11.8, 7.6 Hz, 1H), 7.34 (s, 5H), 5.96 (s, 1H), 5.86 (s, 1H), 3.59 (s, 3H).^13^C NMR (100 MHz, DMSO) δ 157.63, 153.78, 152.50, 152.36, 150.02, 149.87, 147.38, 147.23, 145.56, 144.96, 144.81, 139.18, 131.87, 131.77, 129.05, 129.03, 128.95, 128.93, 128.73, 128.18, 126.98, 120.51, 117.58, 117.40, 104.39, 104.16, 30.33. ^19^F NMR (377 MHz, DMSO ) δ -132.37, -143.87. HRMS (ESI-Q-TOF, m/z) calcd for C_17_H_12_F_2_N_2_O [M+H]^+^: 299.0918, found [M+H]^+^: 299.0968.

***1-methyl-2-oxo-3-(1-phenylvinyl)-1,2-dihydroquinoxaline-6-carbonitrile* (3w)**

Yield: 51%, Yellow solid, m.p.84.3-85.5°C. ^1^H NMR (400 MHz, DMSO-*d*_6_) δ 8.17 (s, 1H), 7.95 (d, *J* = 8.2 Hz, 1H), 7.78 (d, *J* = 8.2 Hz, 1H), 7.40 – 7.29 (m, 5H), 5.96 (d, *J* = 39.2 Hz, 2H), 3.63 (s, 3H). ^13^C NMR (100 MHz, DMSO) δ160.21, 153.87, 145.55, 139.00, 134.89, 134.55, 130.98, 128.78, 128.29, 127.05, 126.75, 121.14, 120.00, 118.94, 112.74, 29.95. HRMS (ESI-Q-TOF, m/z) calcd for C_18_H_13_N_3_O [M+H]^+^: 288.1059, found [M+H]^+^: 288.1023.

***3-(1-phenylvinyl)quinoxalin-2(1H)-one*(3x)**

Yield: 50%, Yellow solid, m.p.183.5-184.5°C. ^1^H NMR (400 MHz, DMSO-*d*_6_) δ 7.75 (d, *J* = 7.4 Hz, 1H), 7.60 – 7.51 (m, 1H), 7.35 (s, 7H), 5.94 (s, 2H). ^13^C NMR (101 MHz, ) δ 157.72, 154.05, 144.99, 138.95, 132.20, 131.62, 130.35, 128.94, 128.65, 128.13, 127.53, 126.53, 123.13, 119.92, 115.12. HRMS (ESI-Q-TOF, m/z) calcd for C_16_H_12_N_2_O [M+H]^+^: 249.0950, found [M+H]^+^: 249.0992.

***1-methyl-3-(1-(phenyl-d5)vinyl-2,2-d2)quinoxalin-2(1H)-one* (3aa)**

Yield: 55%, Yellow solid, m.p.182.8-183.7°C. ^1^H NMR (400 MHz, DMSO-*d*_6_) δ 7.82 (d, *J* = 7.8 Hz, 1H), 7.67 (t, *J* = 7.7 Hz, 1H), 7.59 (d, *J* = 8.2 Hz, 1H), 7.40 (t, *J* = 7.5 Hz, 1H), 3.63 (s, 3H).^13^C NMR (100 MHz, DMSO) δ 156.61, 153.49, 145.15, 138.56, 133.45, 131.95, 130.66, 129.47, 123.36, 114.66, 29.00. HRMS (ESI-Q-TOF, m/z) calcd for C_17_H_7_D_7_N_2_O [M+H]^+^: 270.1546, found [M+H]^+^: 270.1599.

***2-(1-phenylvinyl)quinoxaline*(3A)**

Yield: 41%, Yellow liquid, ^1^H NMR (400 MHz, ) δ 9.05 (s, 1H), 8.25 (dd, J = 14.8, 8.2 Hz, 1H), 8.16 – 8.09 (m, 1H), 8.04 (dt, J = 7.8, 4.1 Hz, 2H), 7.43 (s, 5H), 6.18 (s, 1H), 5.99 (s, 1H). ^13^C NMR (100 MHz, DMSO) δ 145.53, 141.92, 141.54, 141.48, 139.39, 133.49, 132.25, 131.06, 130.64, 130.35, 129.70, 129.67, 129.29, 128.92, 128.69, 128.58, 121.67. HRMS (ESI-Q-TOF, m/z) calcd for C_16_H_12_N_2_ [M+H]^+^: 233.1000, found [M+H]^+^: 233.1024.

**Ⅲ. Copies of ^1^H NMR and ^13^C NMR Spectra**


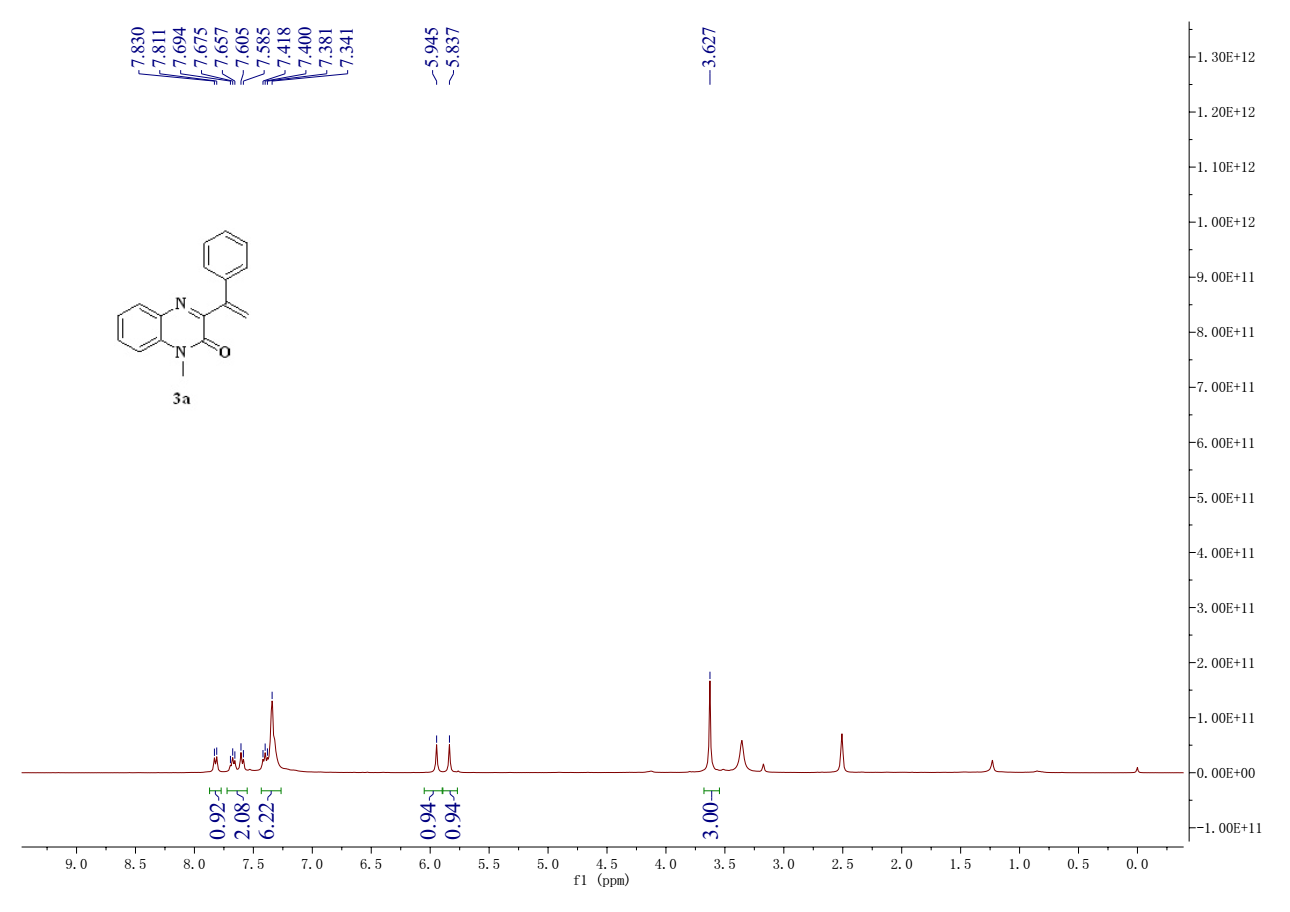


^1^H NMR spectrum of compound **3a** (400 MHz, DMSO-*d*_6_)


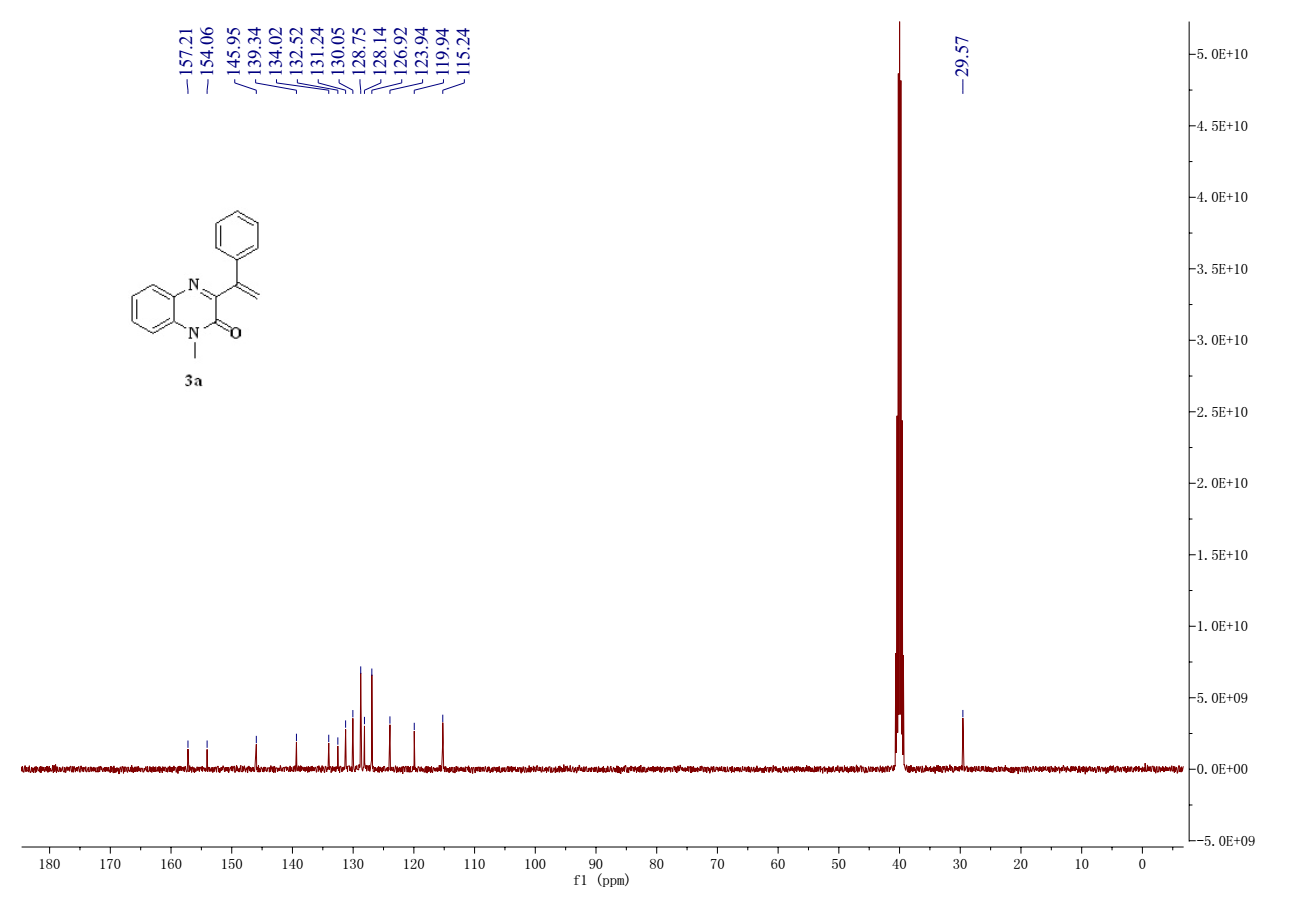


^13^C NMR spectrum of compound **3a** (100 MHz, DMSO-*d*_6_


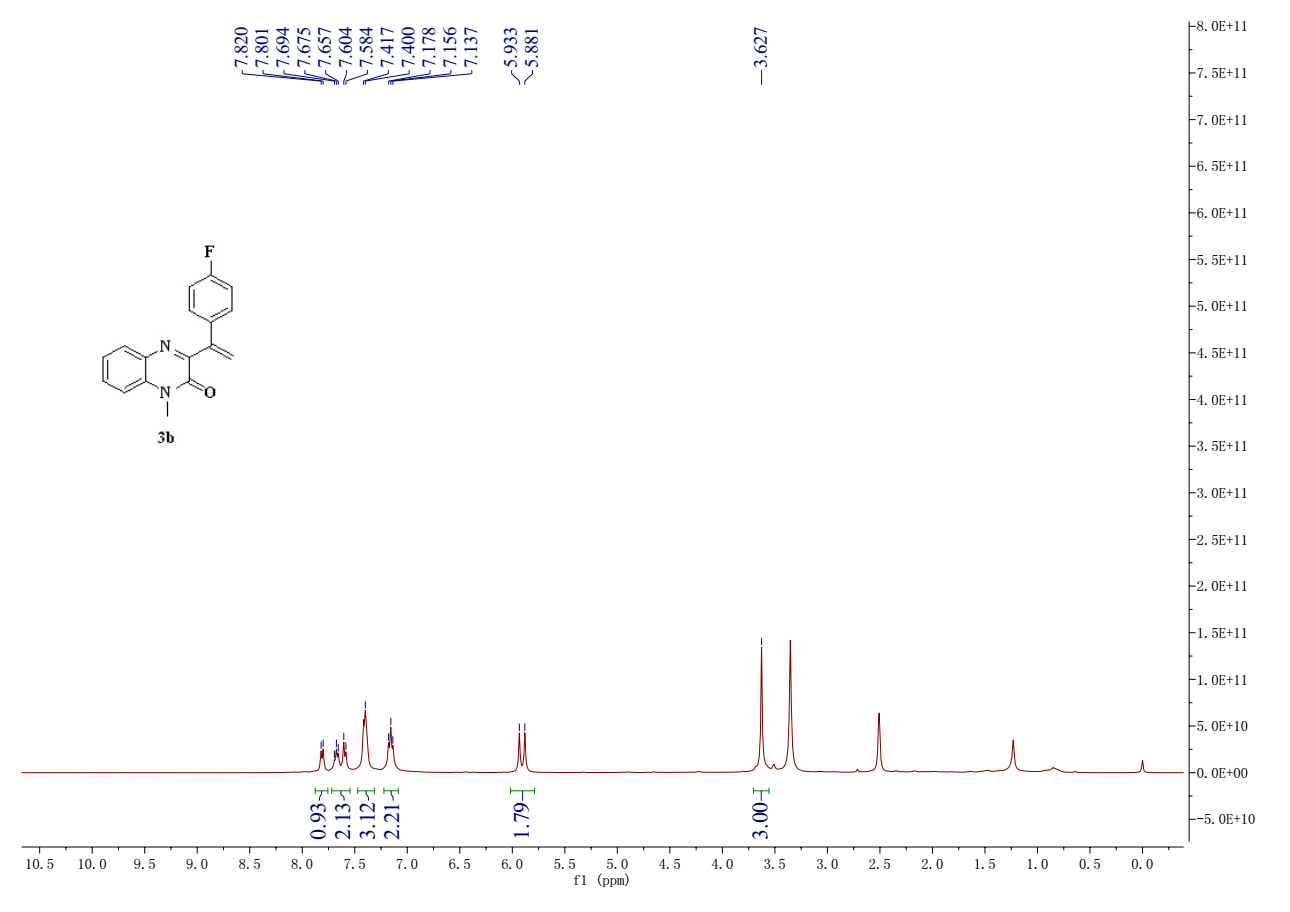


^1^H NMR spectrum of compound **3b** (400 MHz, DMSO-*d*_6_)


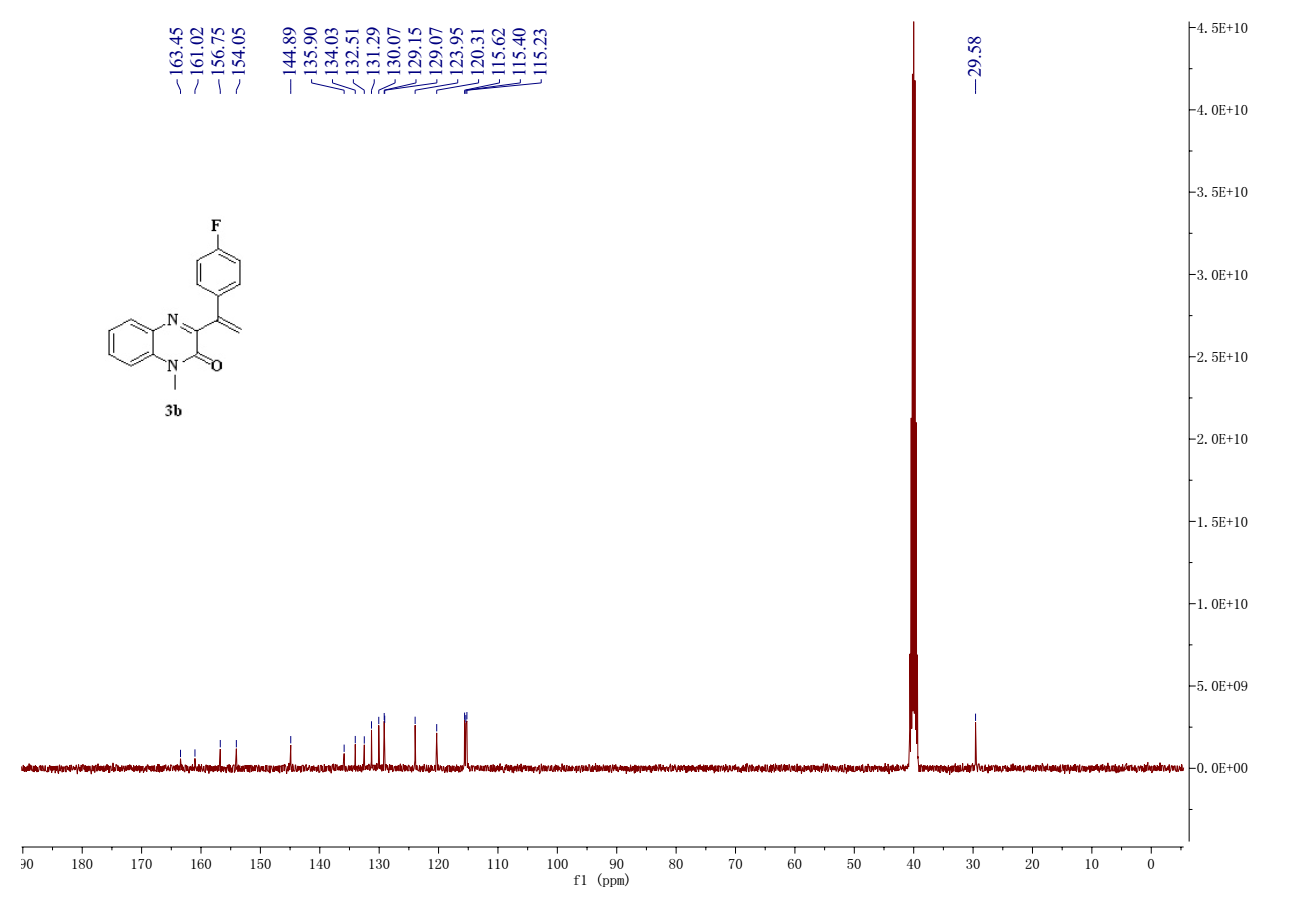


^13^C NMR spectrum of compound **3b** (100 MHz, DMSO-*d*_6_)


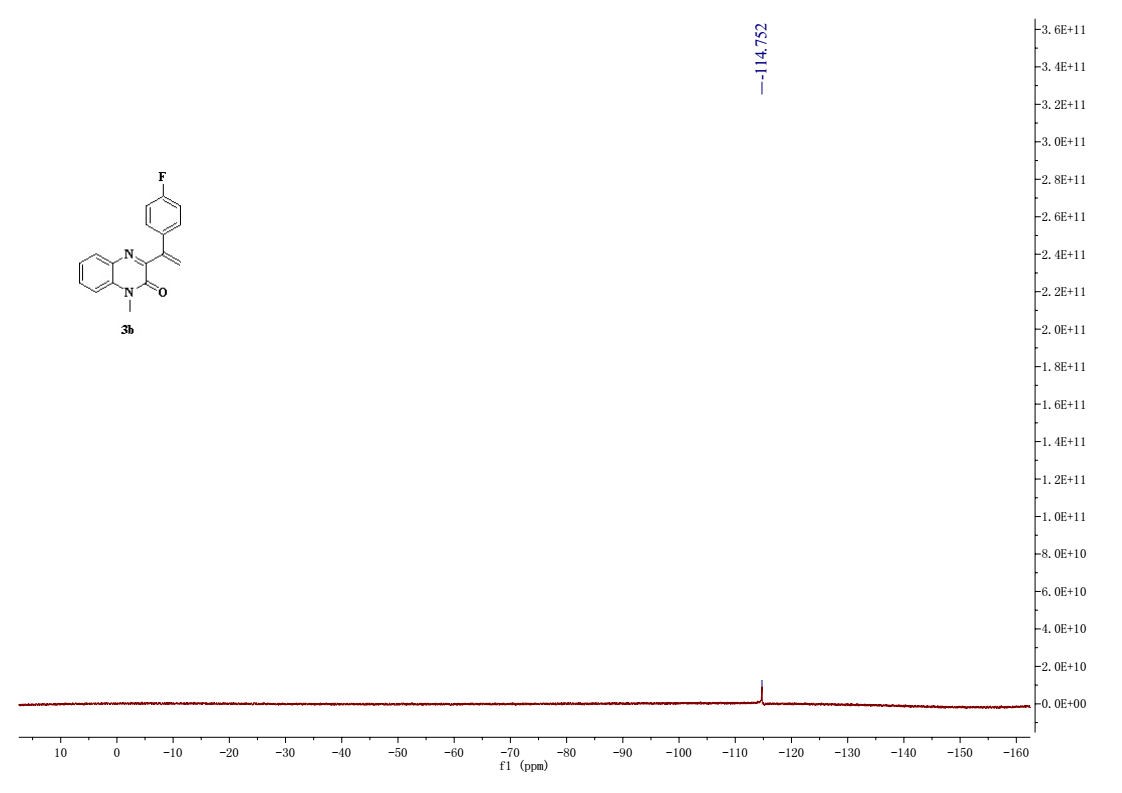


^19^F NMR spectrum of compound **3b** (377 MHz, DMSO-*d*_6_)


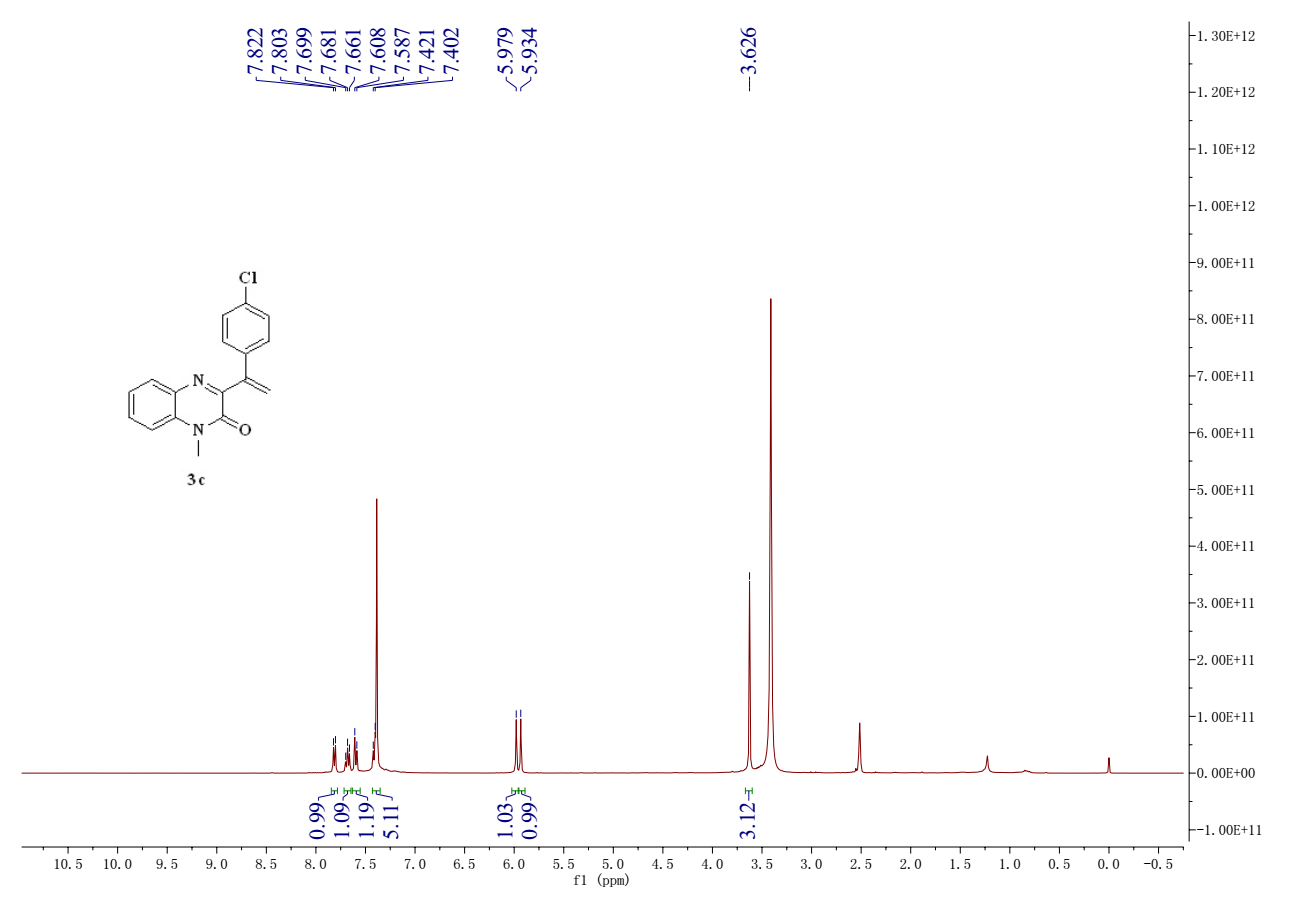


^1^H NMR spectrum of compound **3c** (400 MHz, DMSO-*d*_6_)


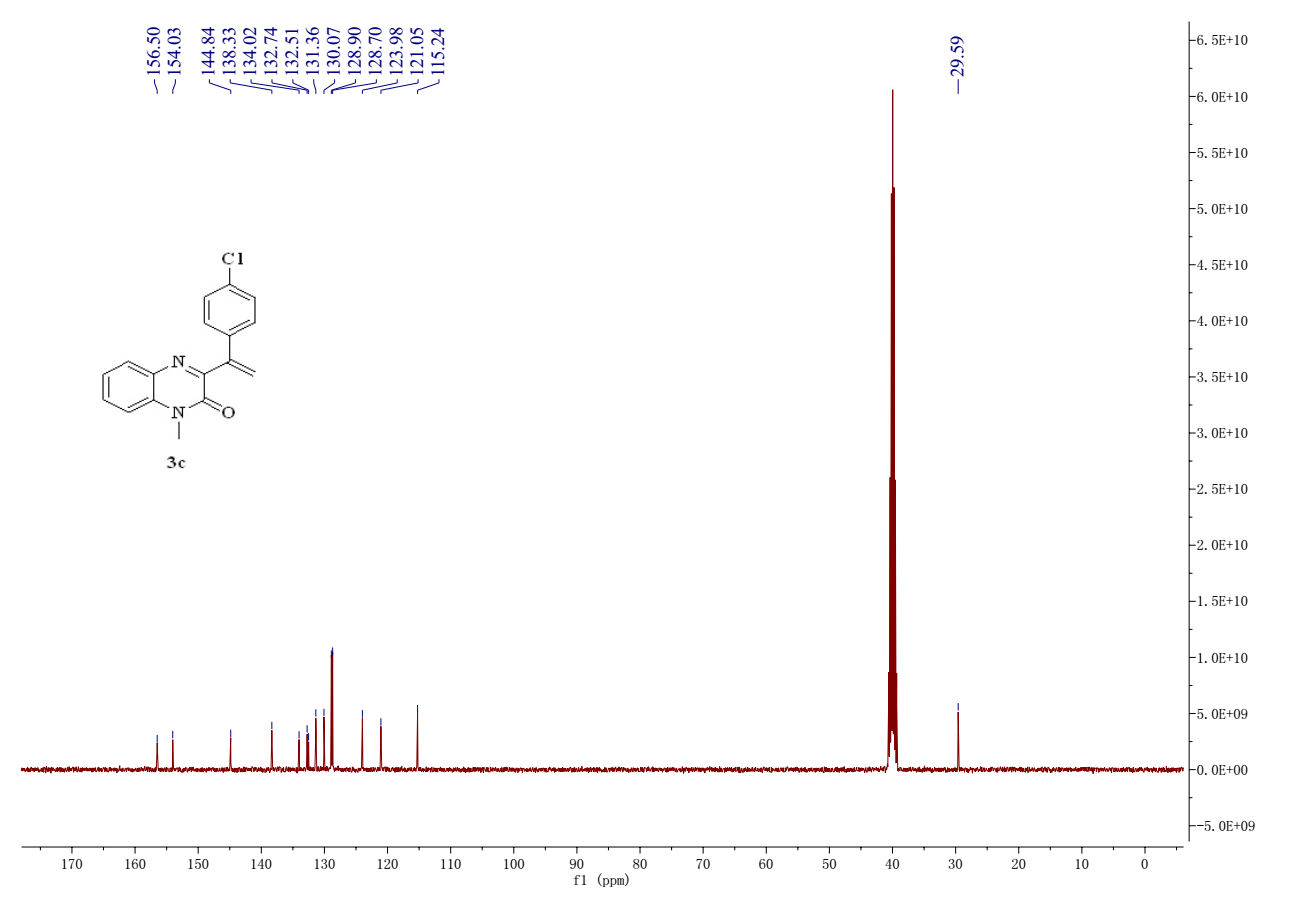


^13^C NMR spectrum of compound **3c** (100 MHz, DMSO-*d*_6_)


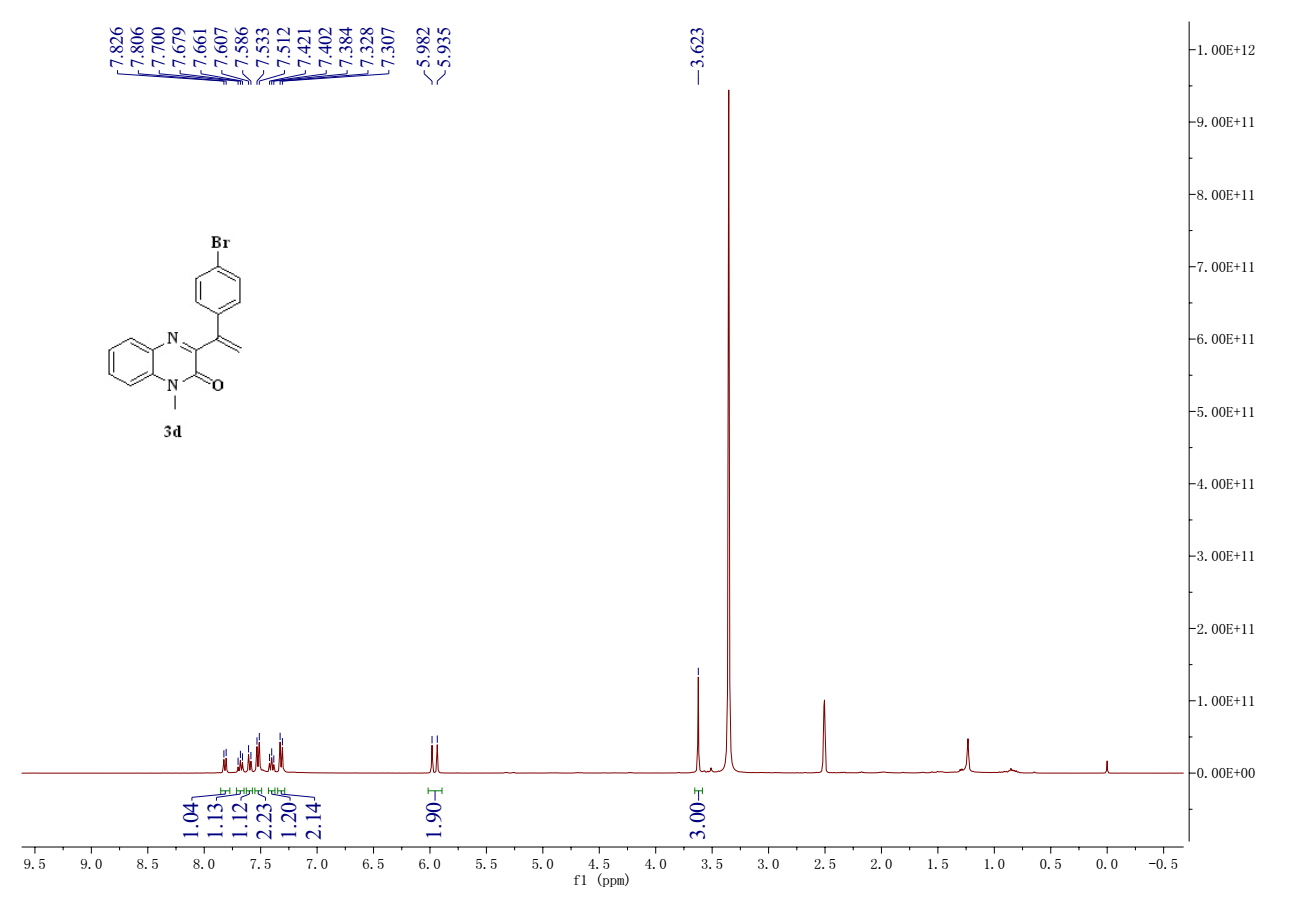


^1^H NMR spectrum of compound **3d** (400 MHz, DMSO-*d*_6_)


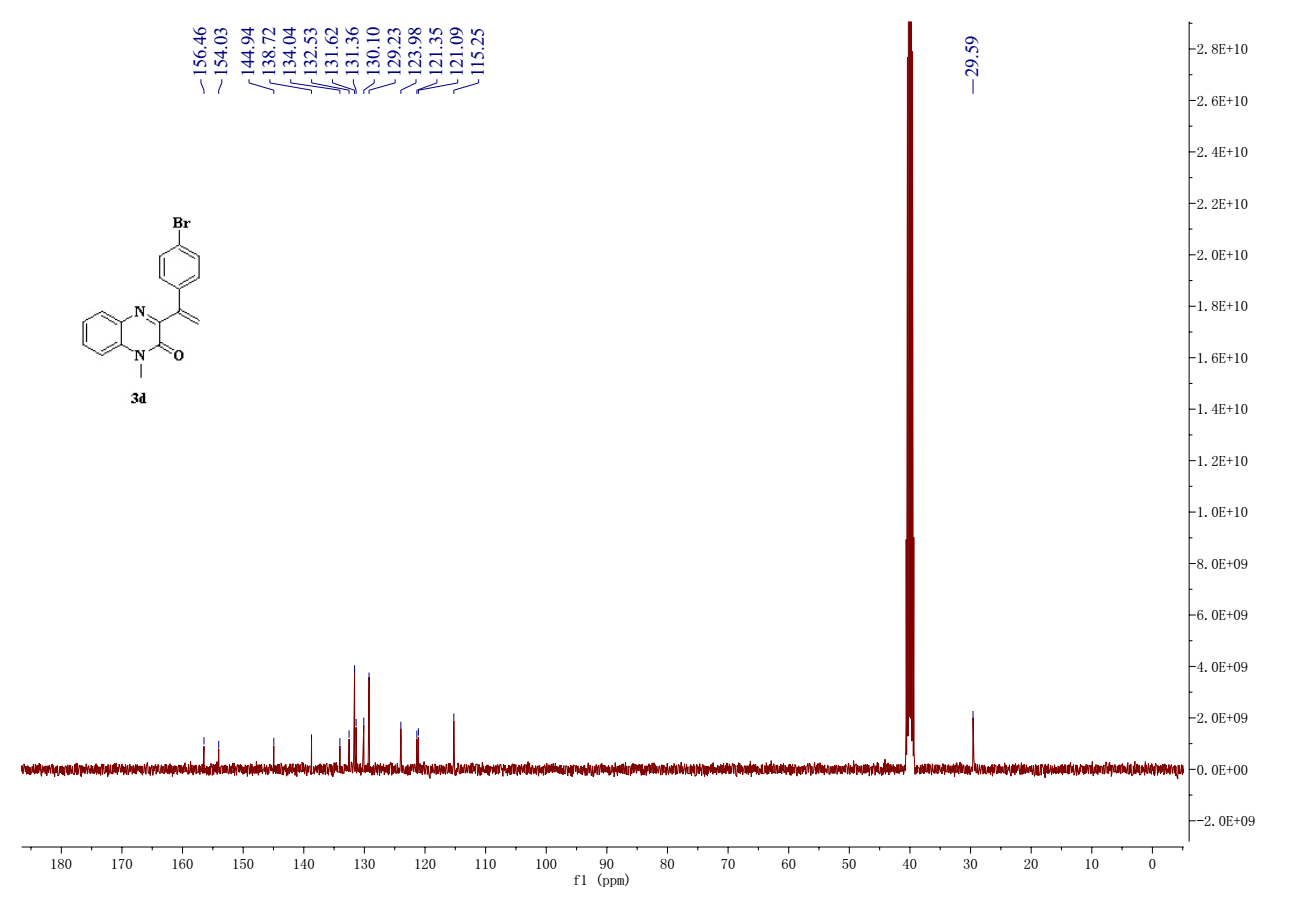


^13^C NMR spectrum of compound **3d** (100 MHz, DMSO-*d*_6_)


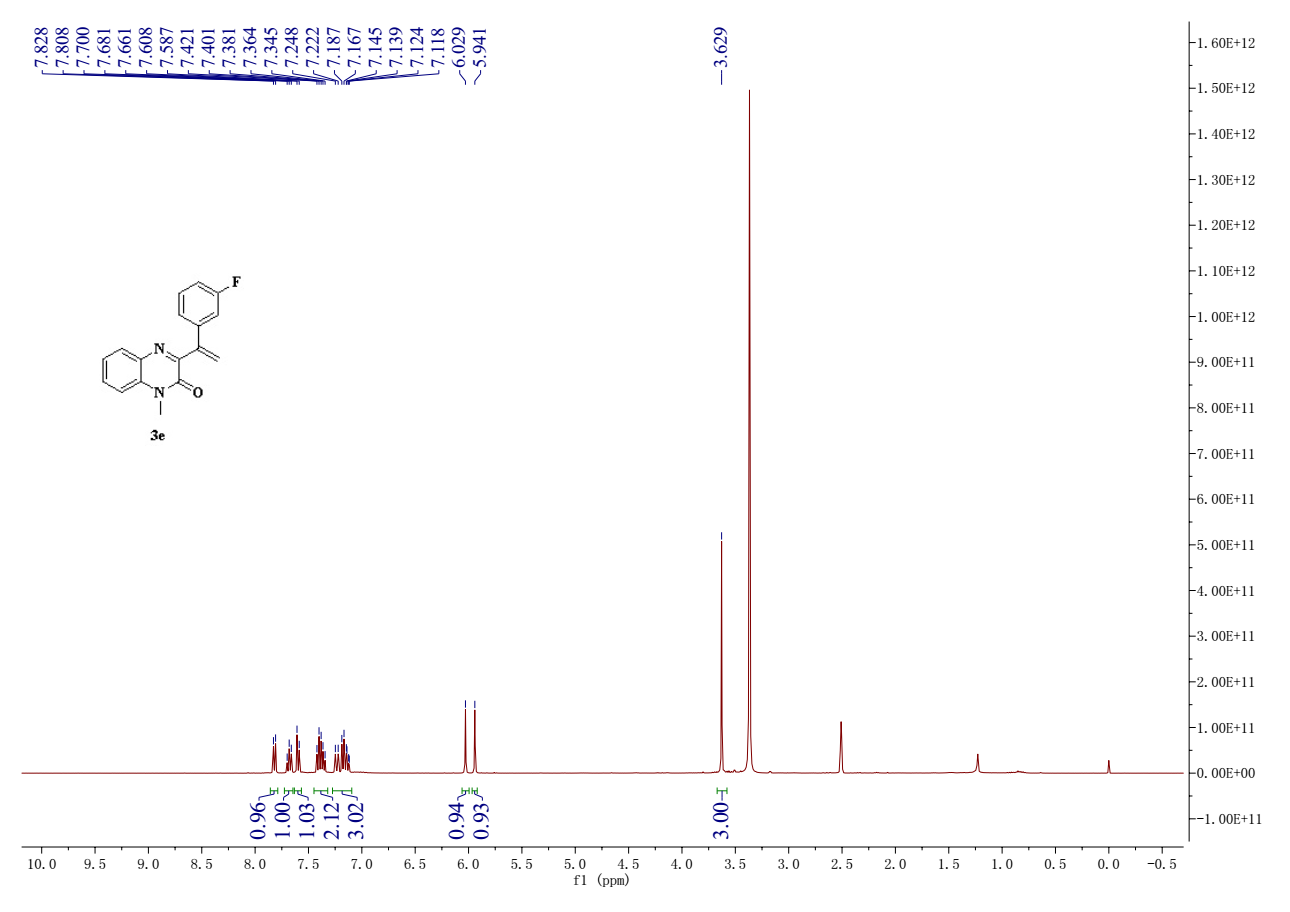


^1^H NMR spectrum of compound **3e** (400 MHz, DMSO-*d*_6_)


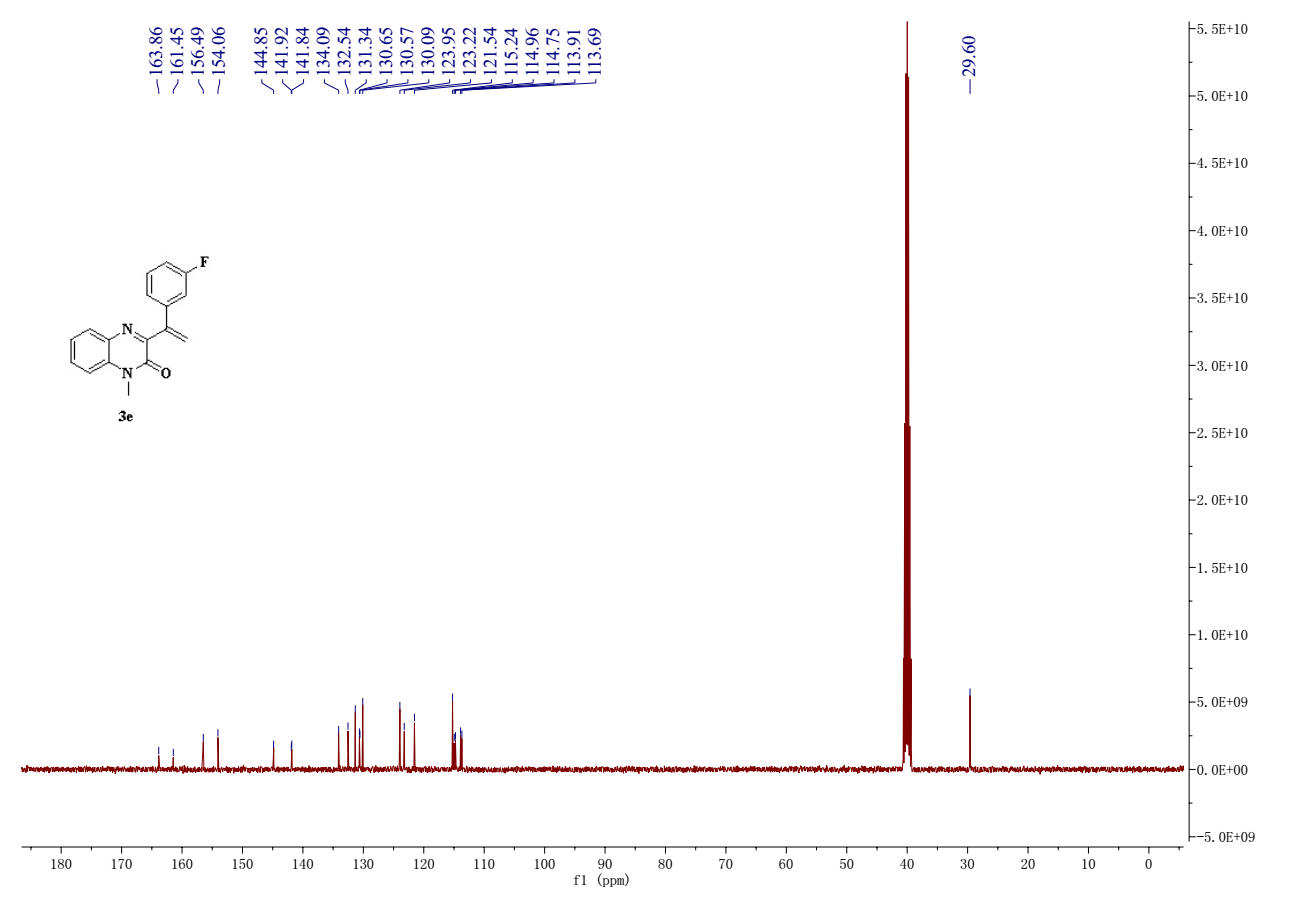


^13^C NMR spectrum of compound **3e** (100 MHz, DMSO-*d*_6_)


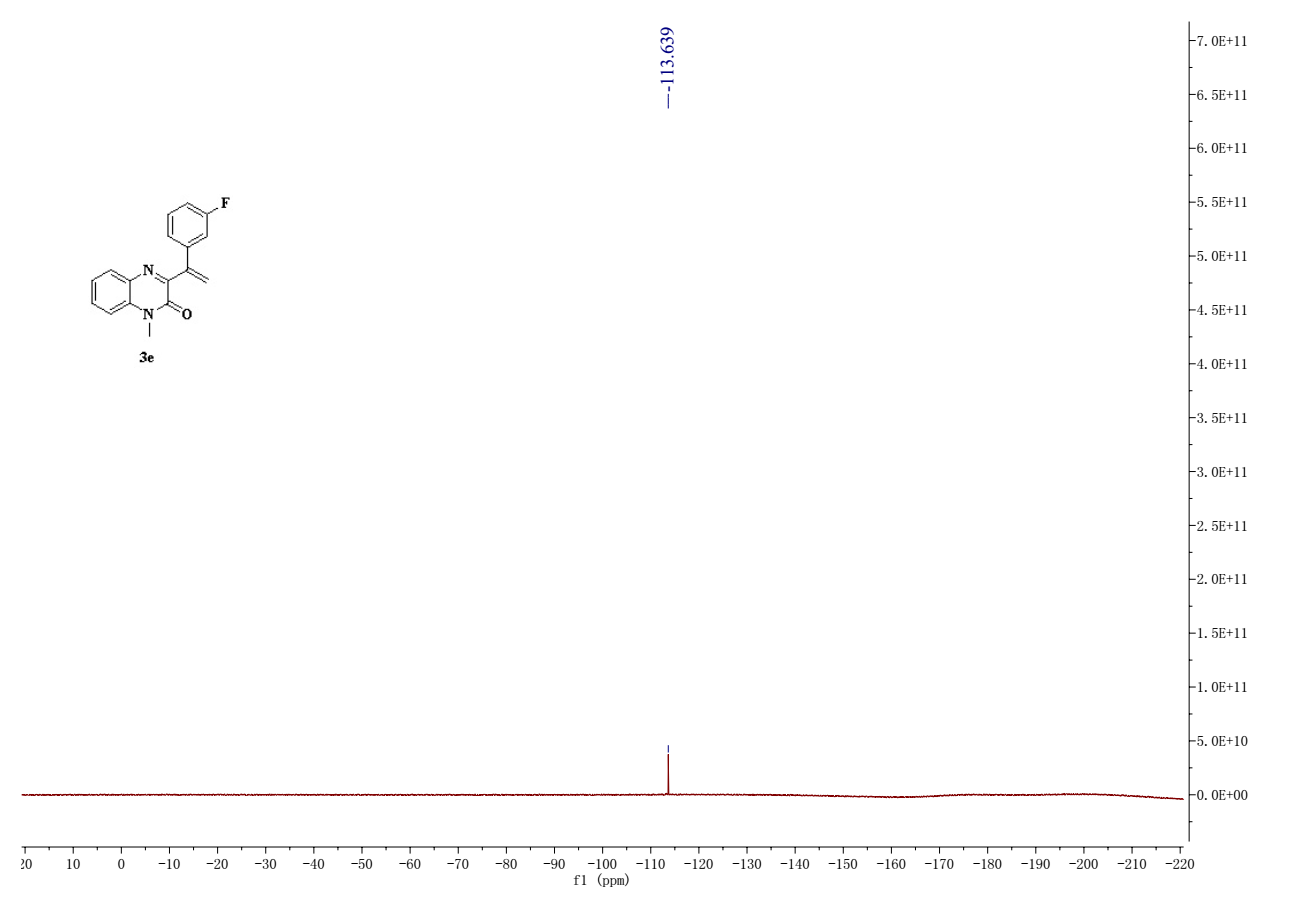


^19^F NMR spectrum of compound **3e** (377 MHz, DMSO-*d*_6_)


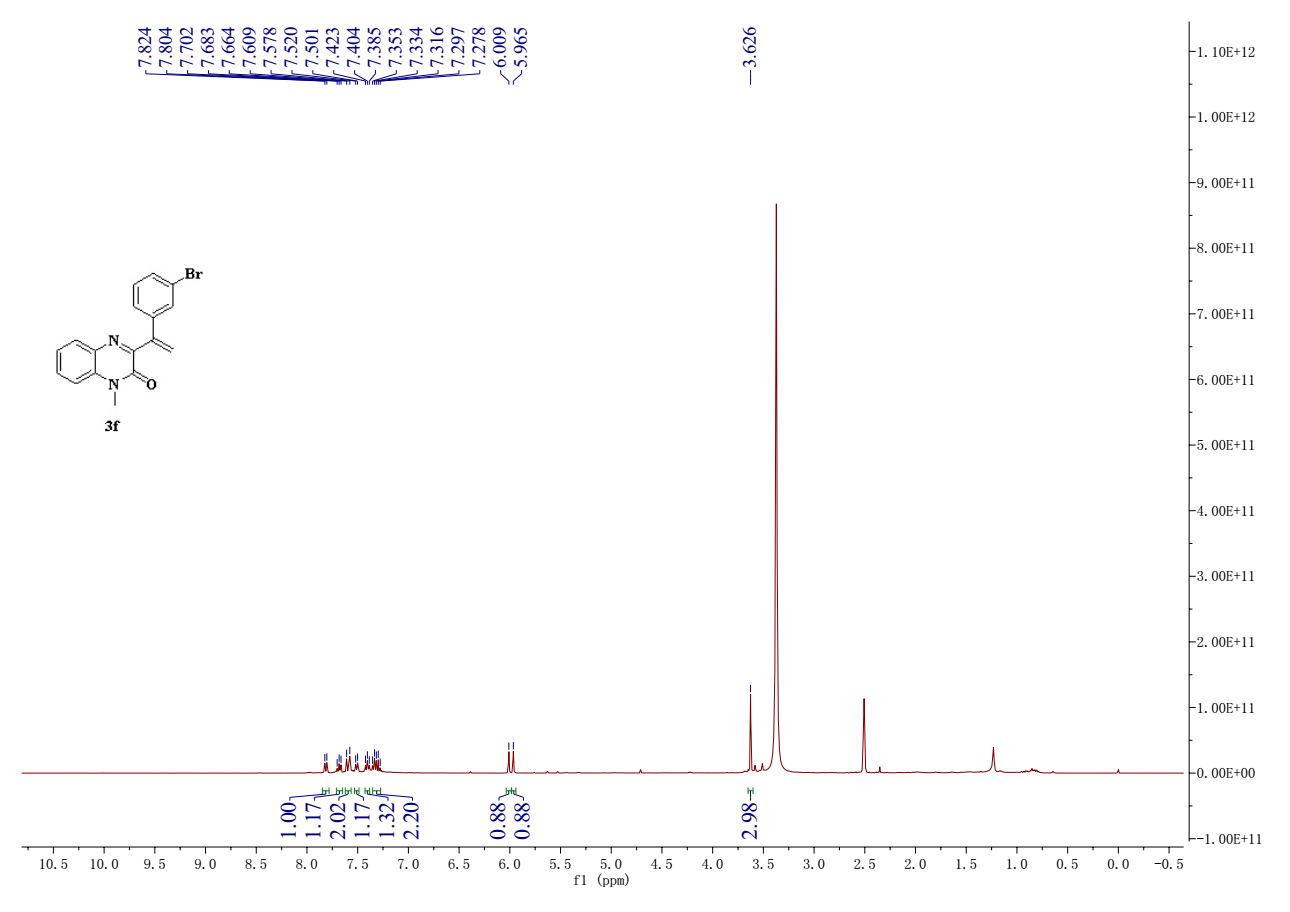


^1^H NMR spectrum of compound **3f** (400 MHz, DMSO-*d*_6_)


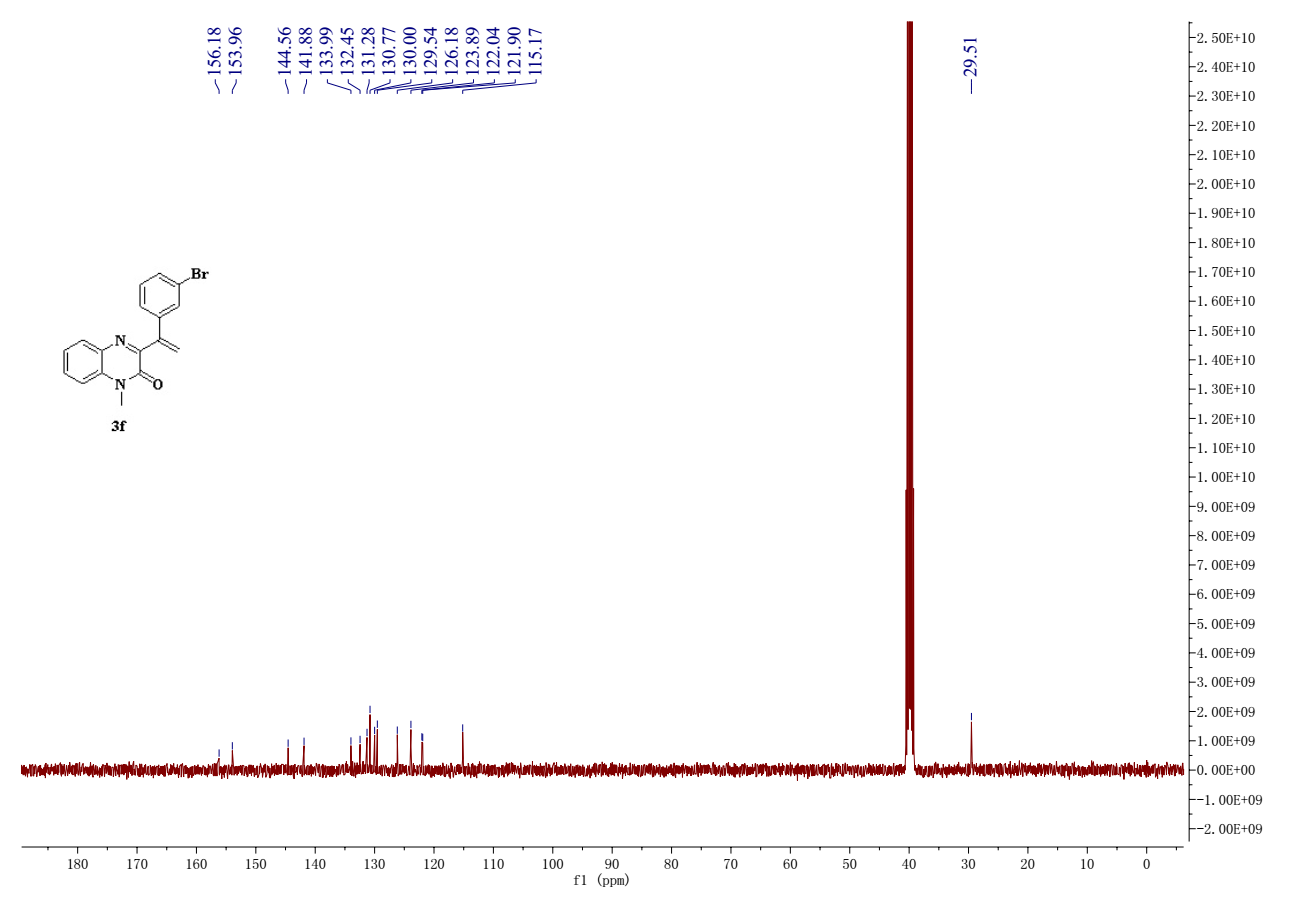


^13^C NMR spectrum of compound **3f** (100 MHz, DMSO-*d*_6_)


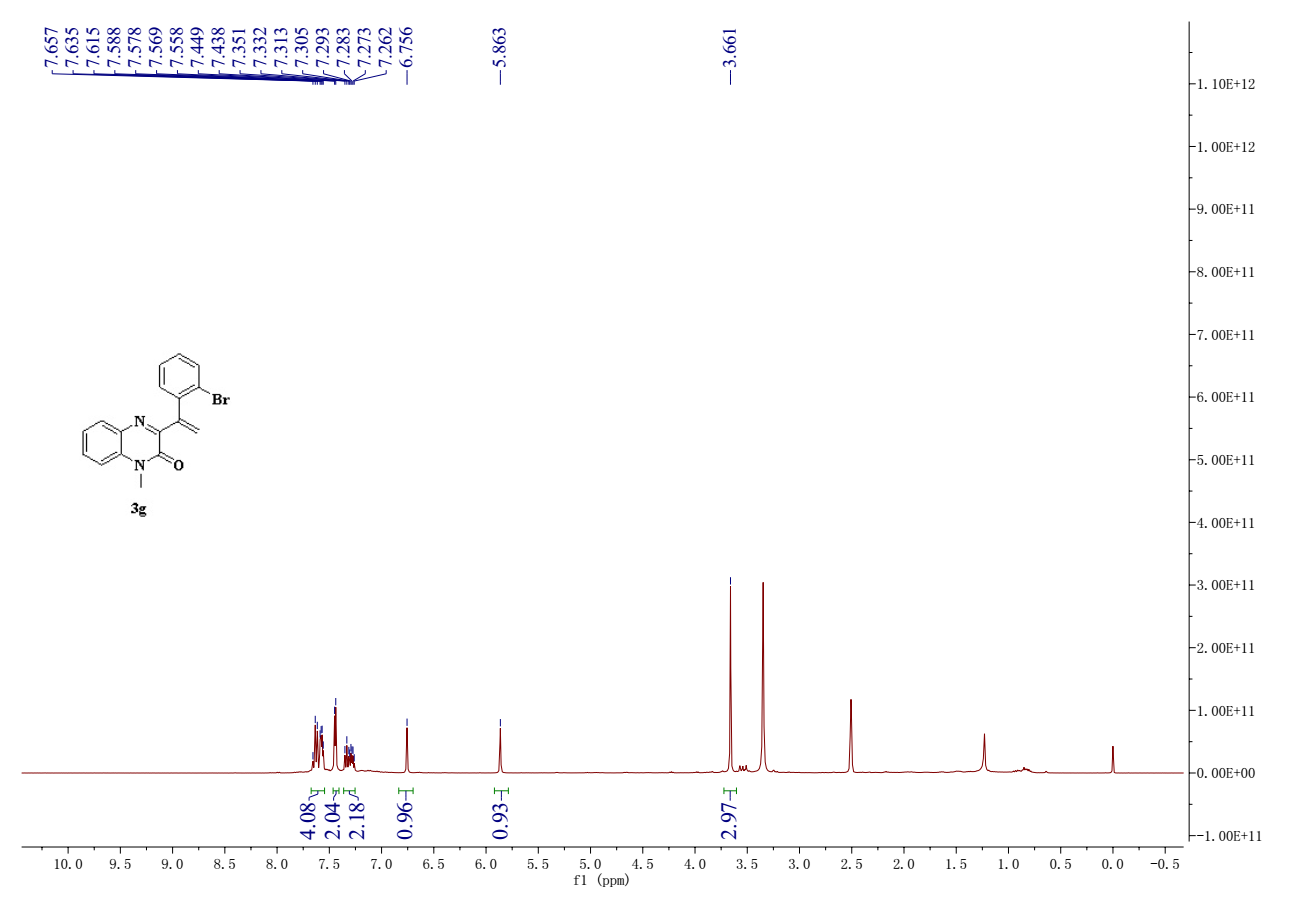


^1^H NMR spectrum of compound **3g** (400 MHz, DMSO-*d*_6_)


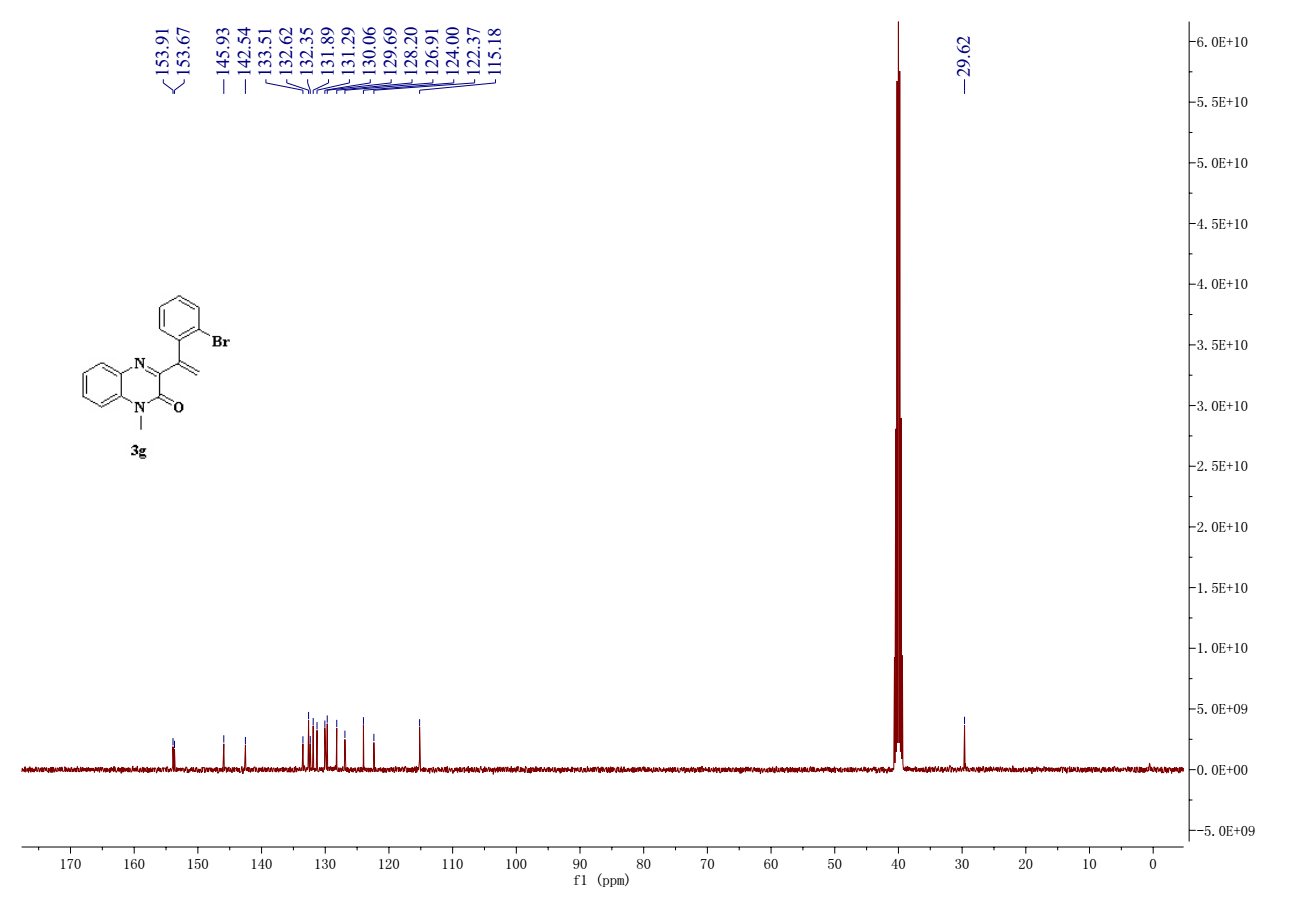


^13^C NMR spectrum of compound **3g** (100 MHz, DMSO-*d*_6_)


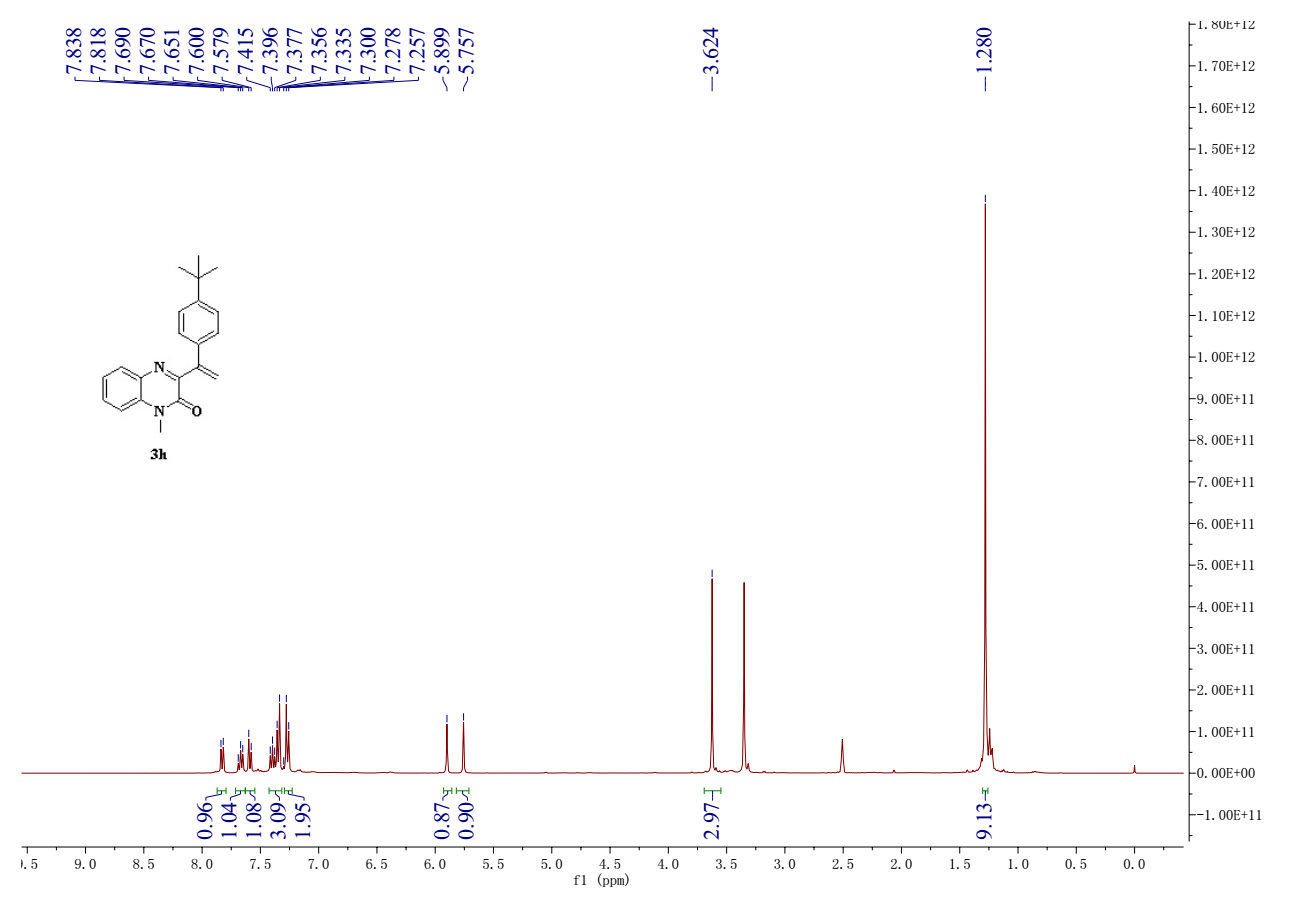


^1^H NMR spectrum of compound **3h** (400 MHz, DMSO-*d*_6_)


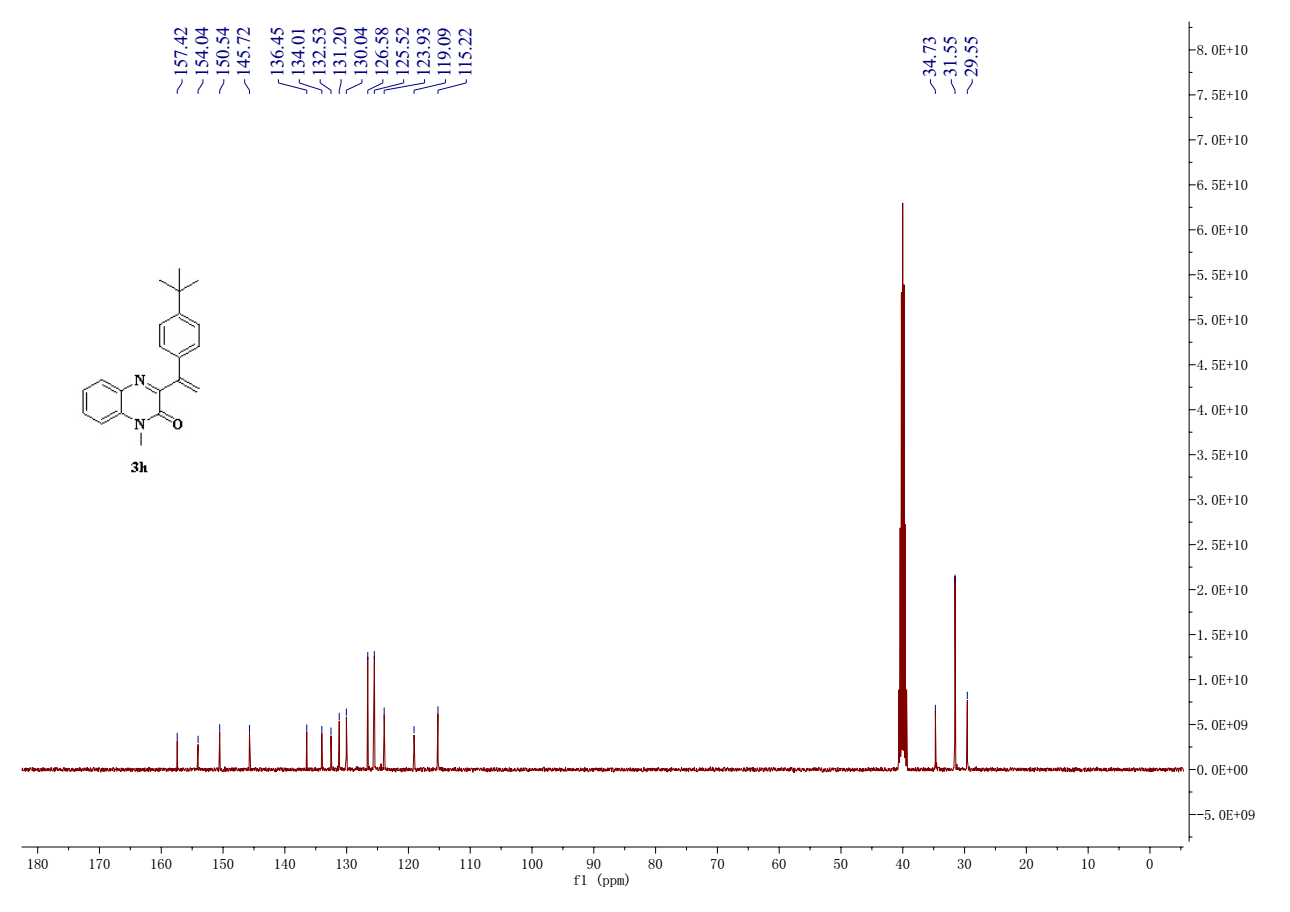


^13^C NMR spectrum of compound **3h** (100 MHz, DMSO-*d*_6_)


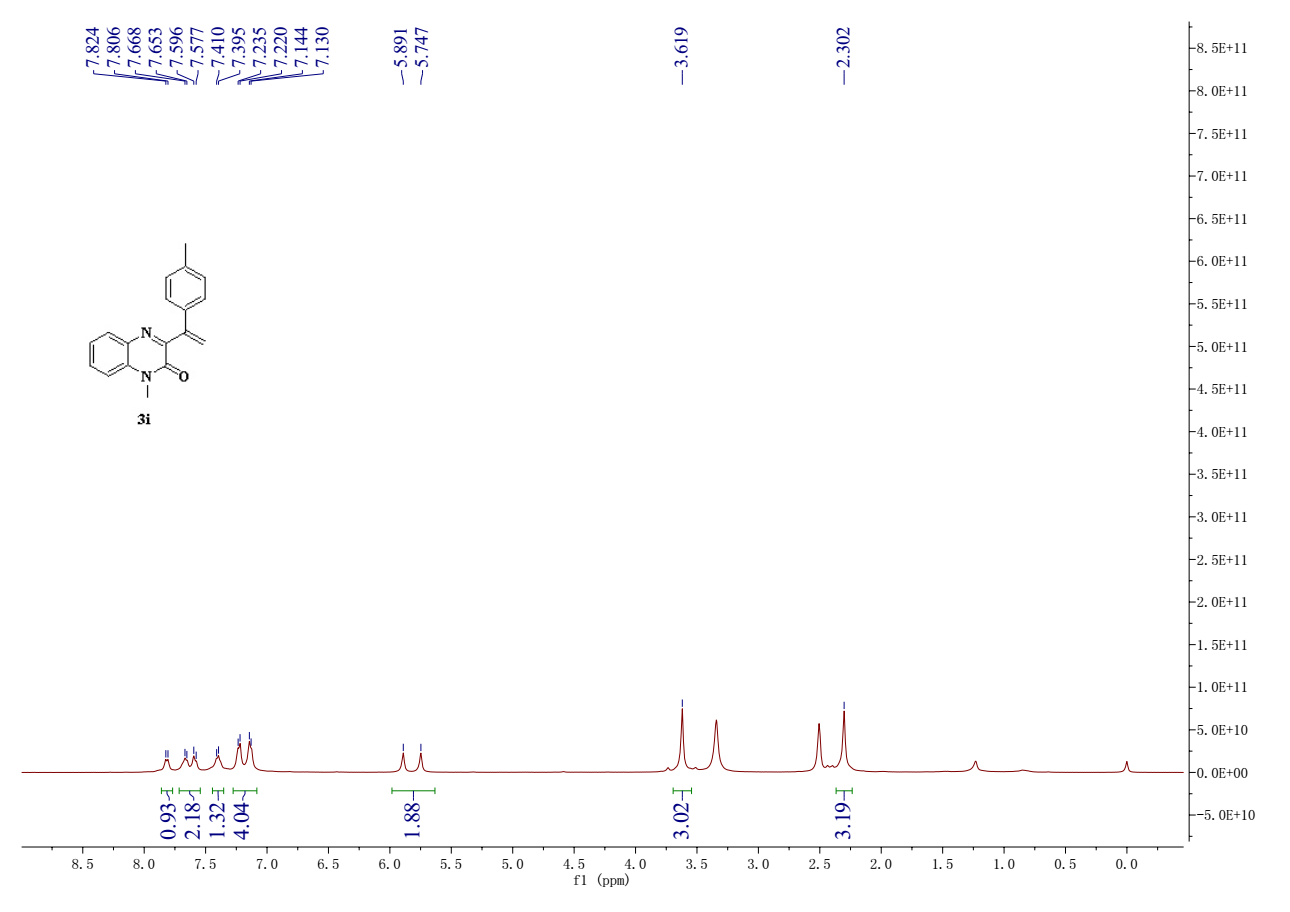


^1^H NMR spectrum of compound **3i** (400 MHz, DMSO-*d*_6_)


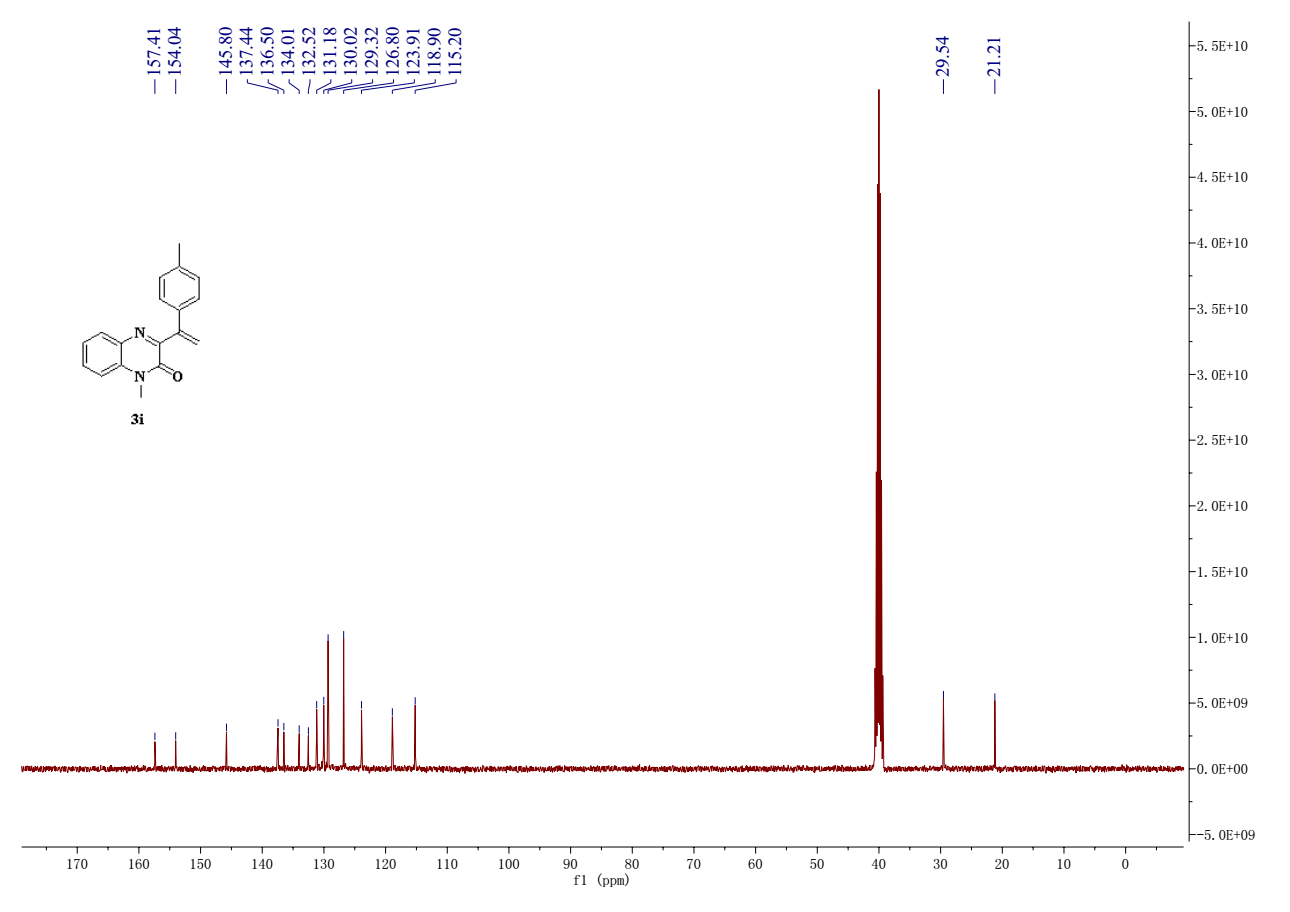


^13^C NMR spectrum of compound **3i** (100 MHz, DMSO-*d*_6_)


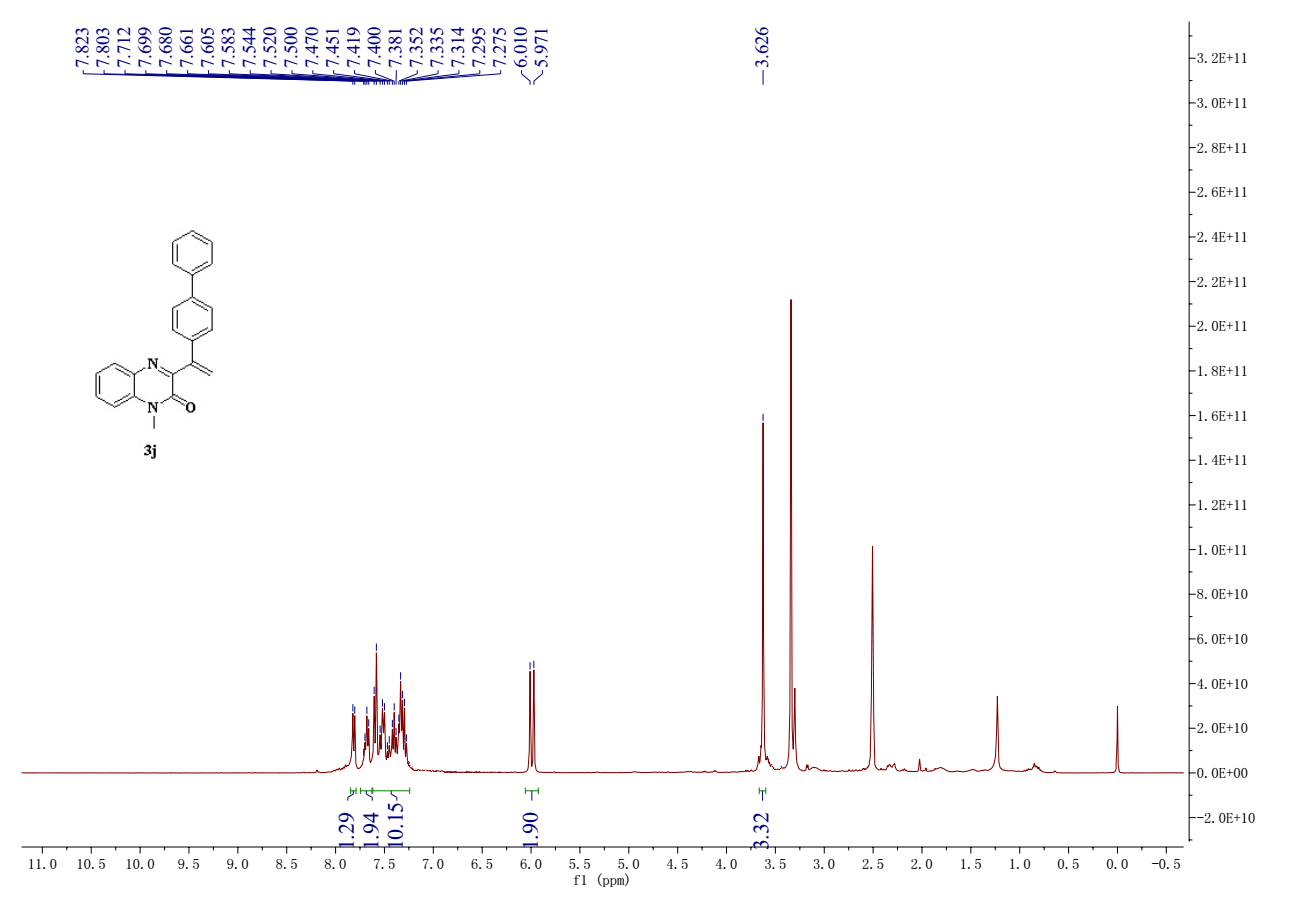


^1^H NMR spectrum of compound **3j** (400 MHz, DMSO-*d*_6_)


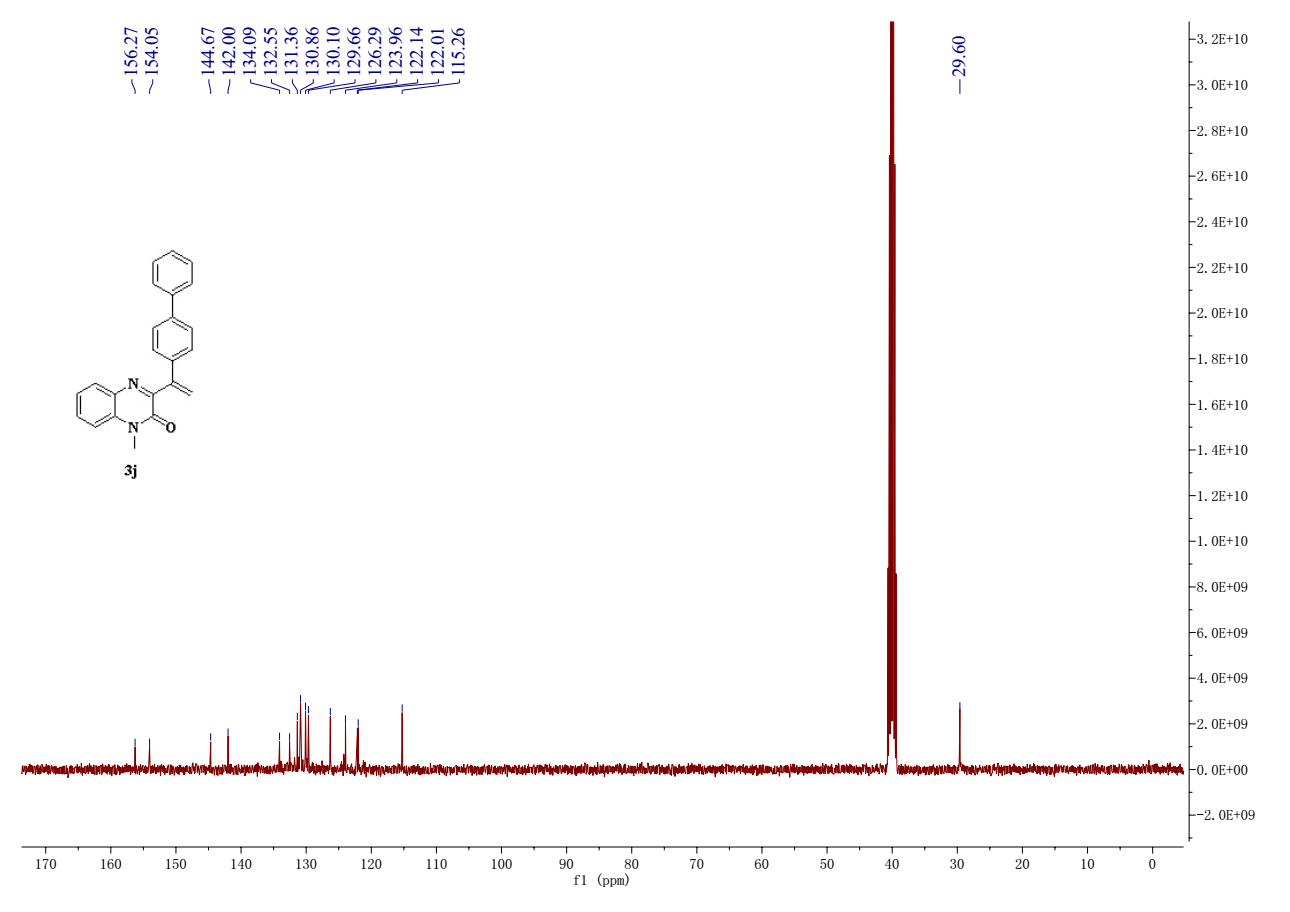


^13^C NMR spectrum of compound **3j** (100 MHz, DMSO-*d*_6_)


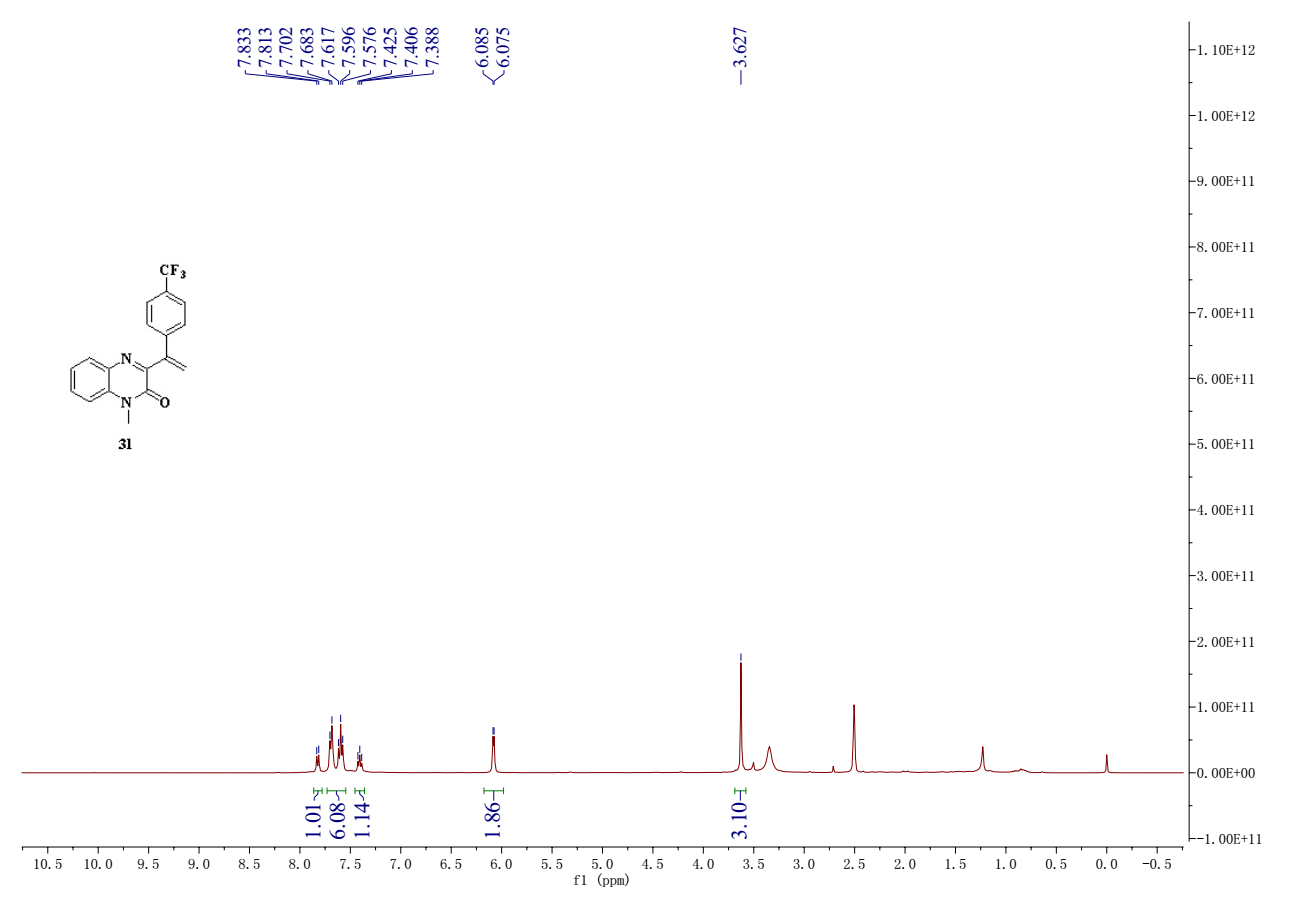


^1^H NMR spectrum of compound **3l** (400 MHz, DMSO-*d*_6_)


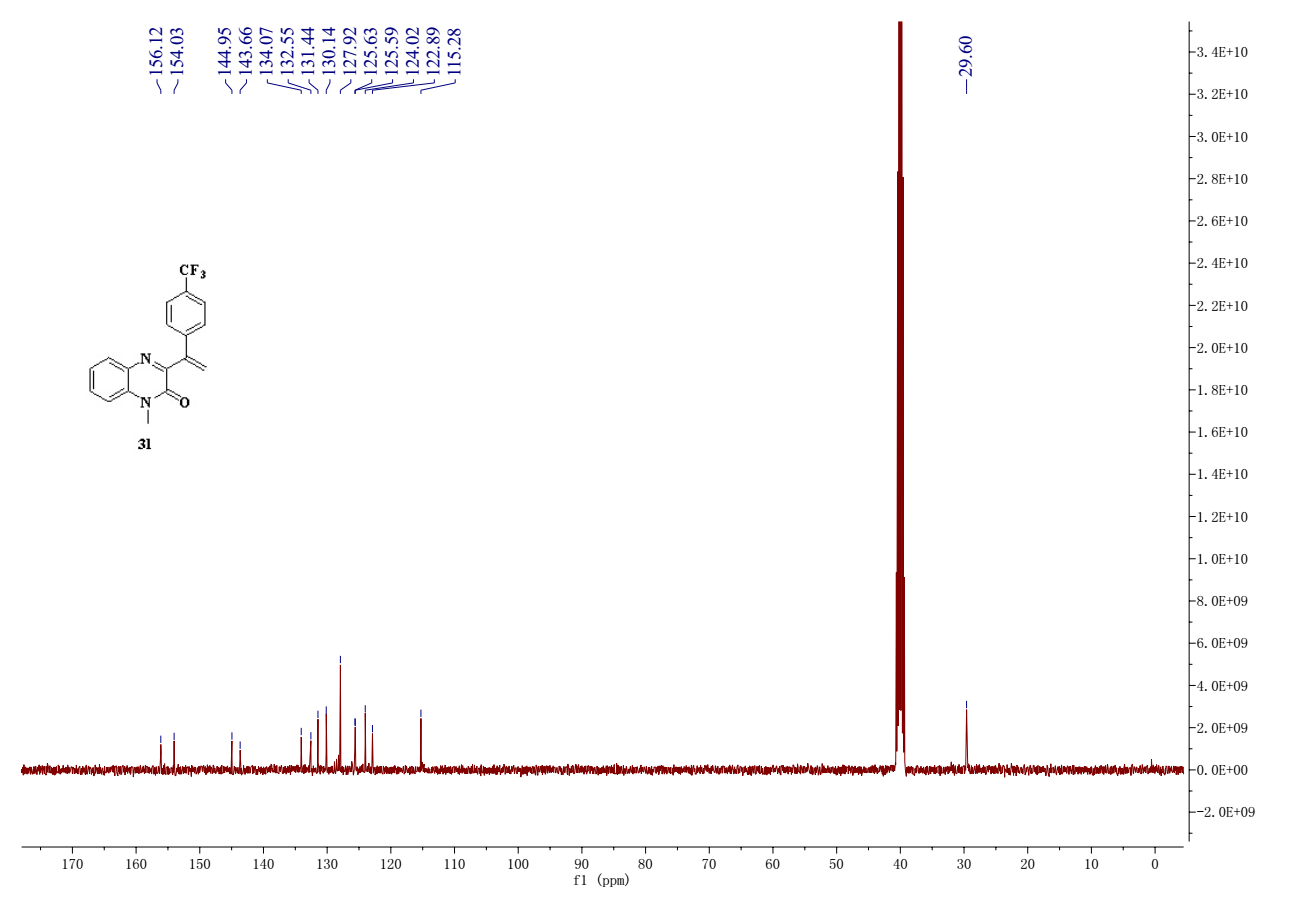


^13^C NMR spectrum of compound **3l** (100 MHz, DMSO-*d*_6_)


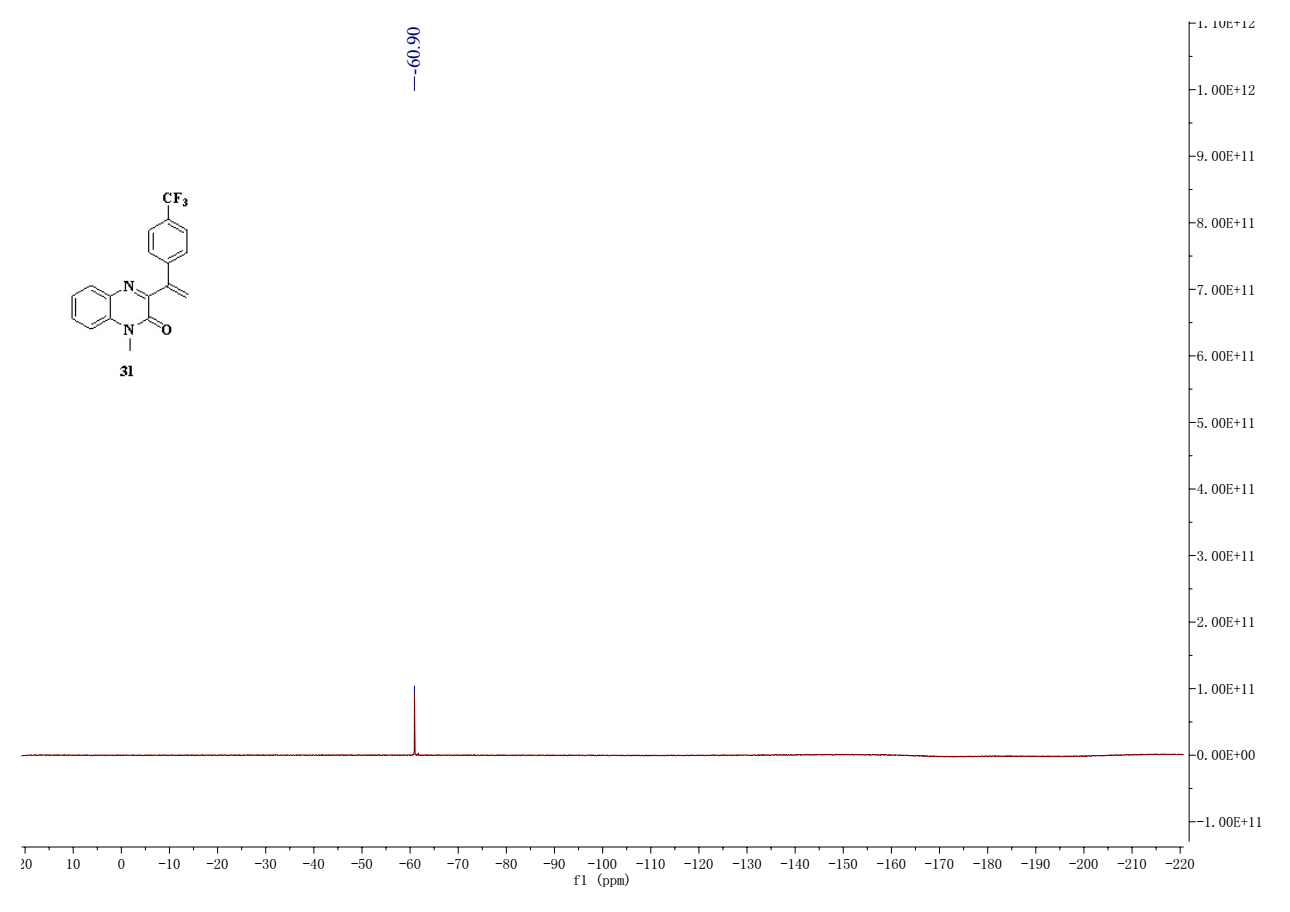


^19^F NMR spectrum of compound **3l** (377 MHz, DMSO-*d*_6_)


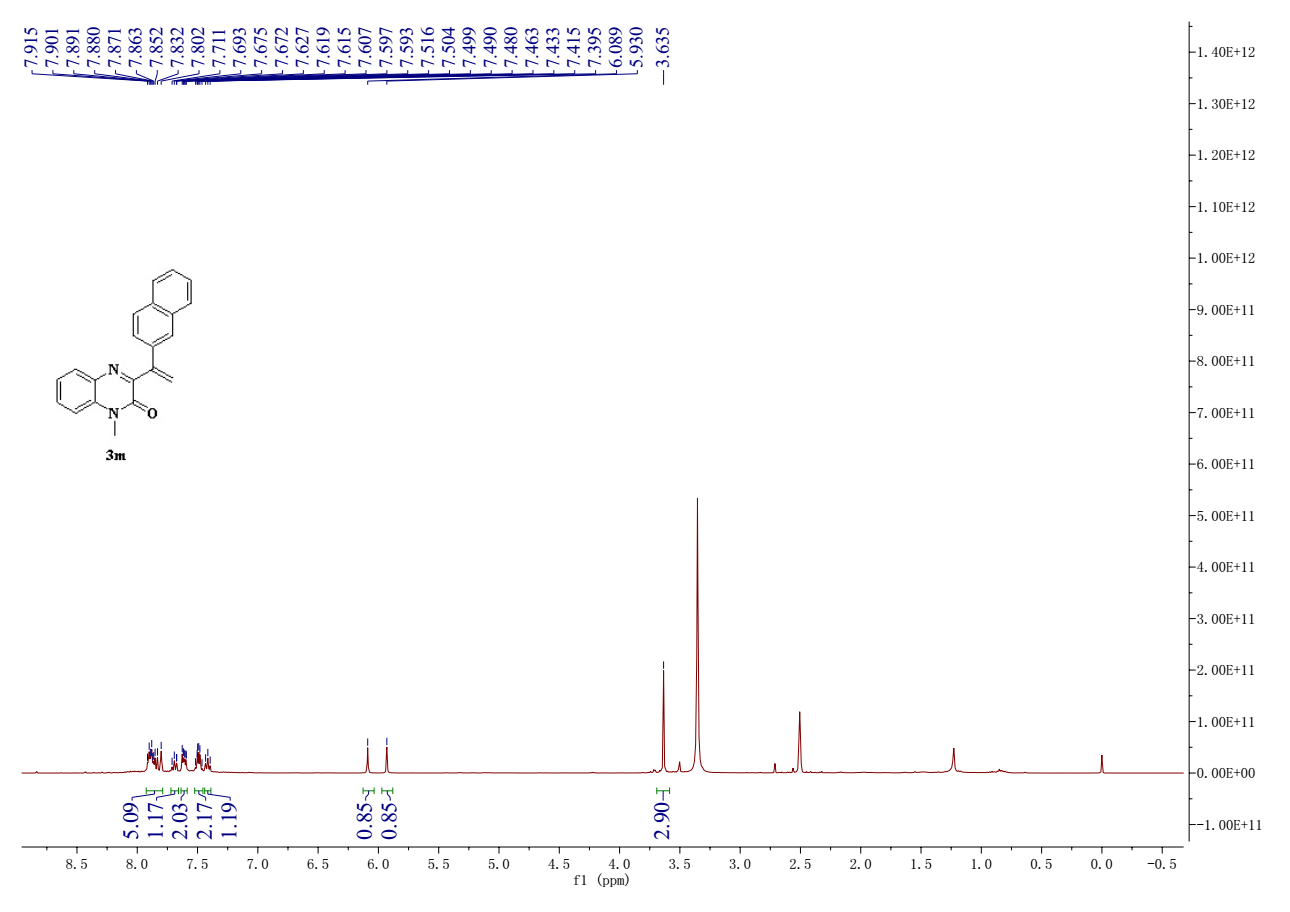


^1^H NMR spectrum of compound **3m** (400 MHz, DMSO-*d*_6_)


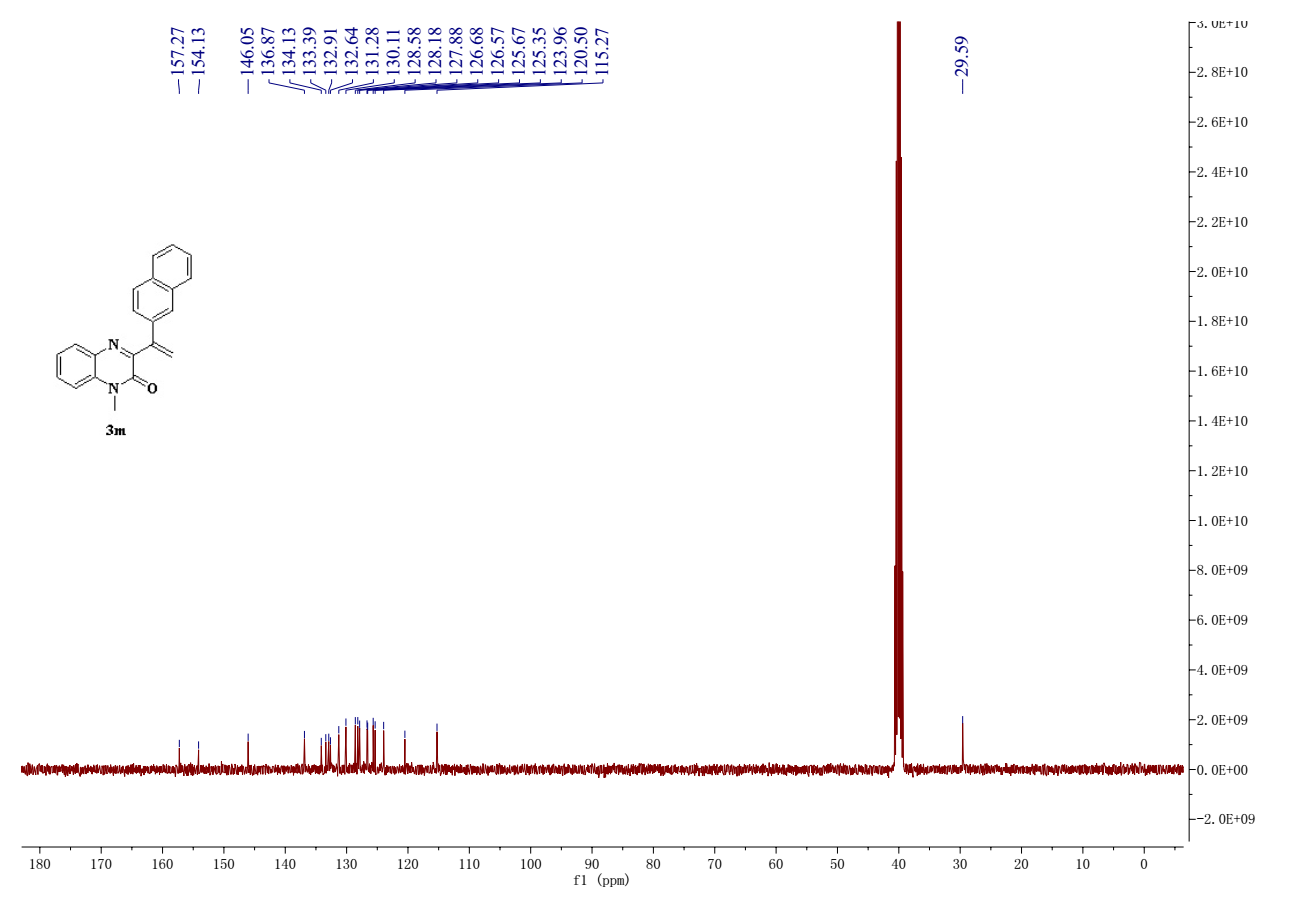


^13^C NMR spectrum of compound **3m** (100 MHz, DMSO-*d*_6_)


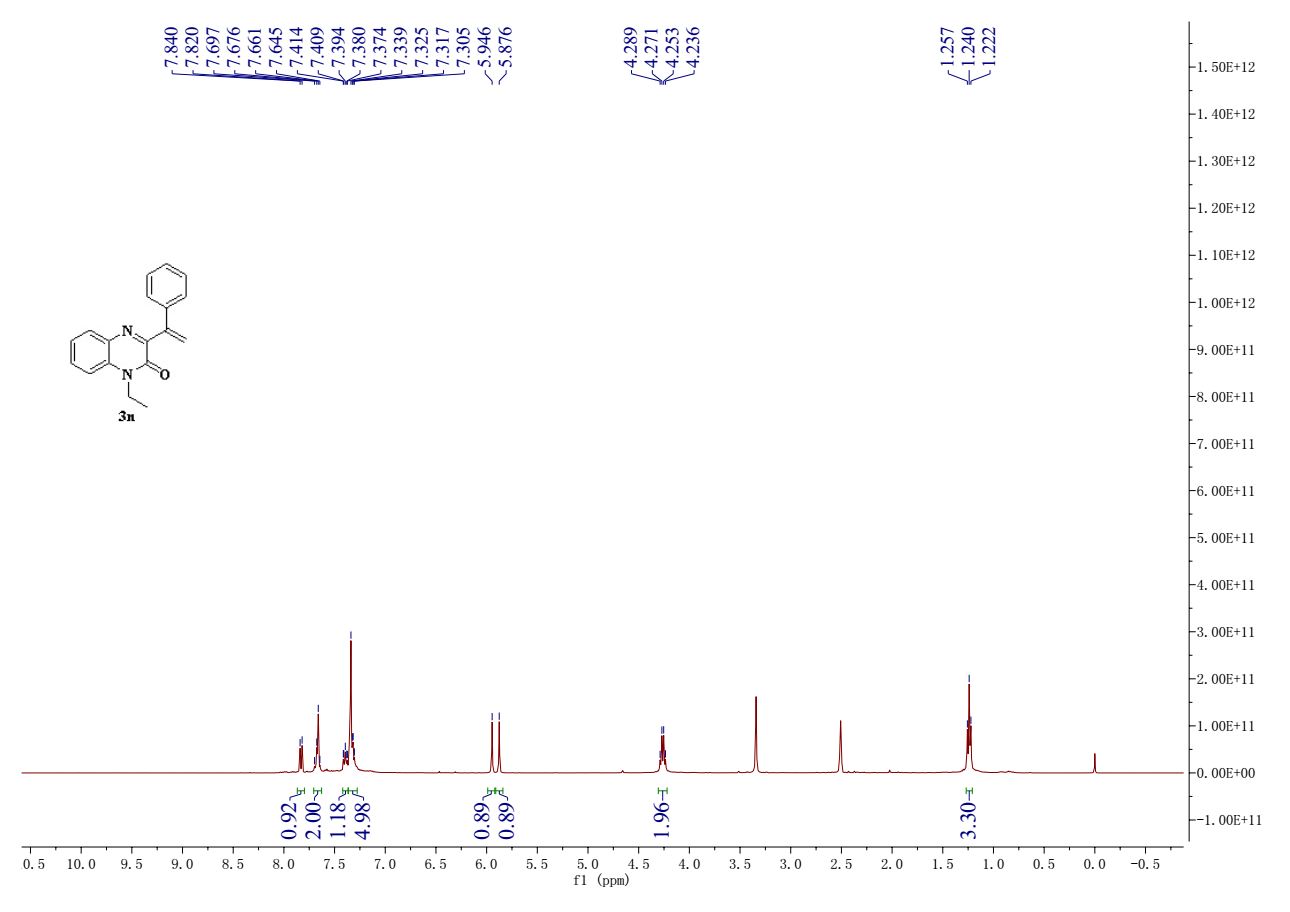


^1^H NMR spectrum of compound **3n** (400 MHz, DMSO-*d*_6_)


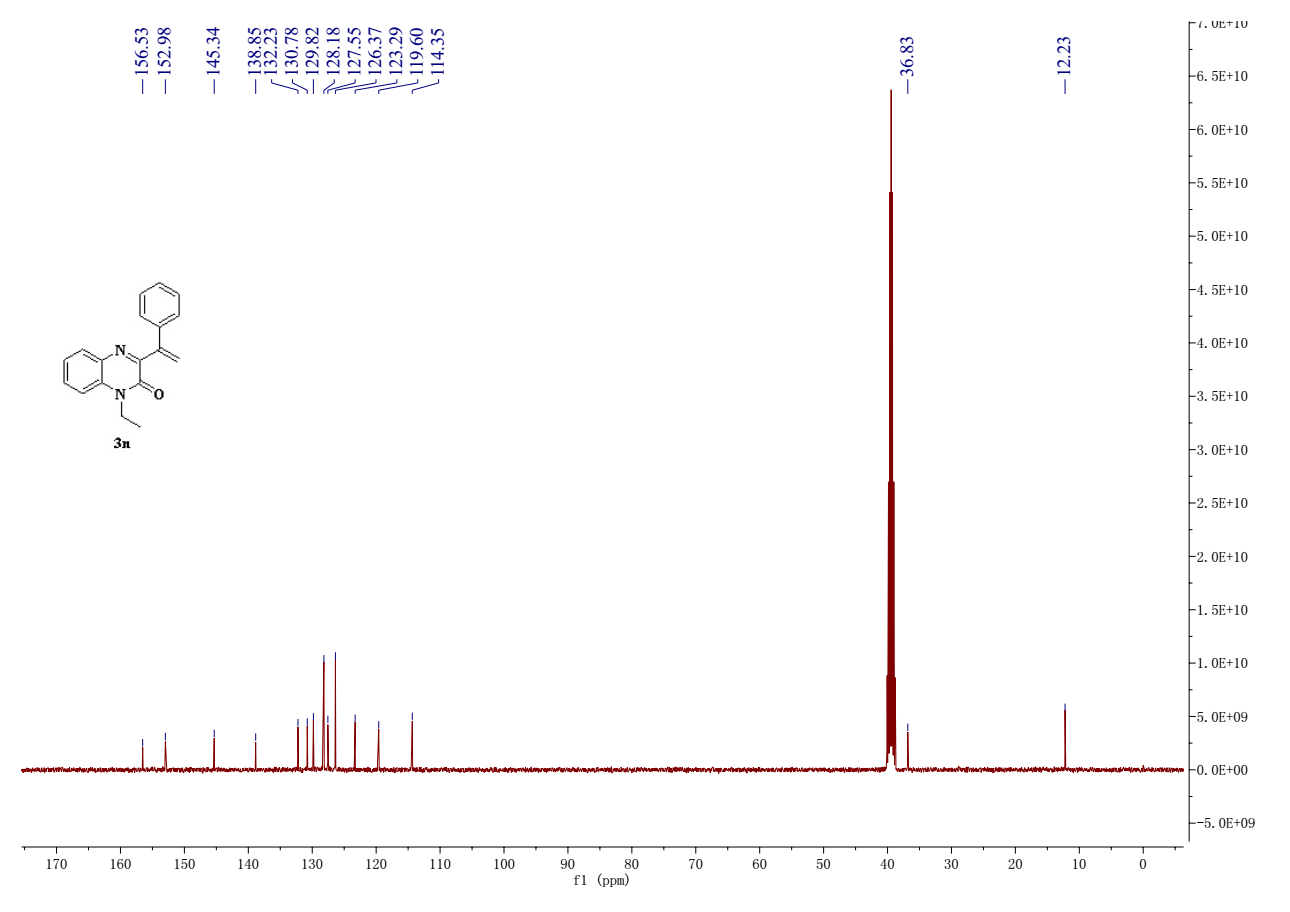


^13^C NMR spectrum of compound **3n** (100 MHz, DMSO-*d*_6_)


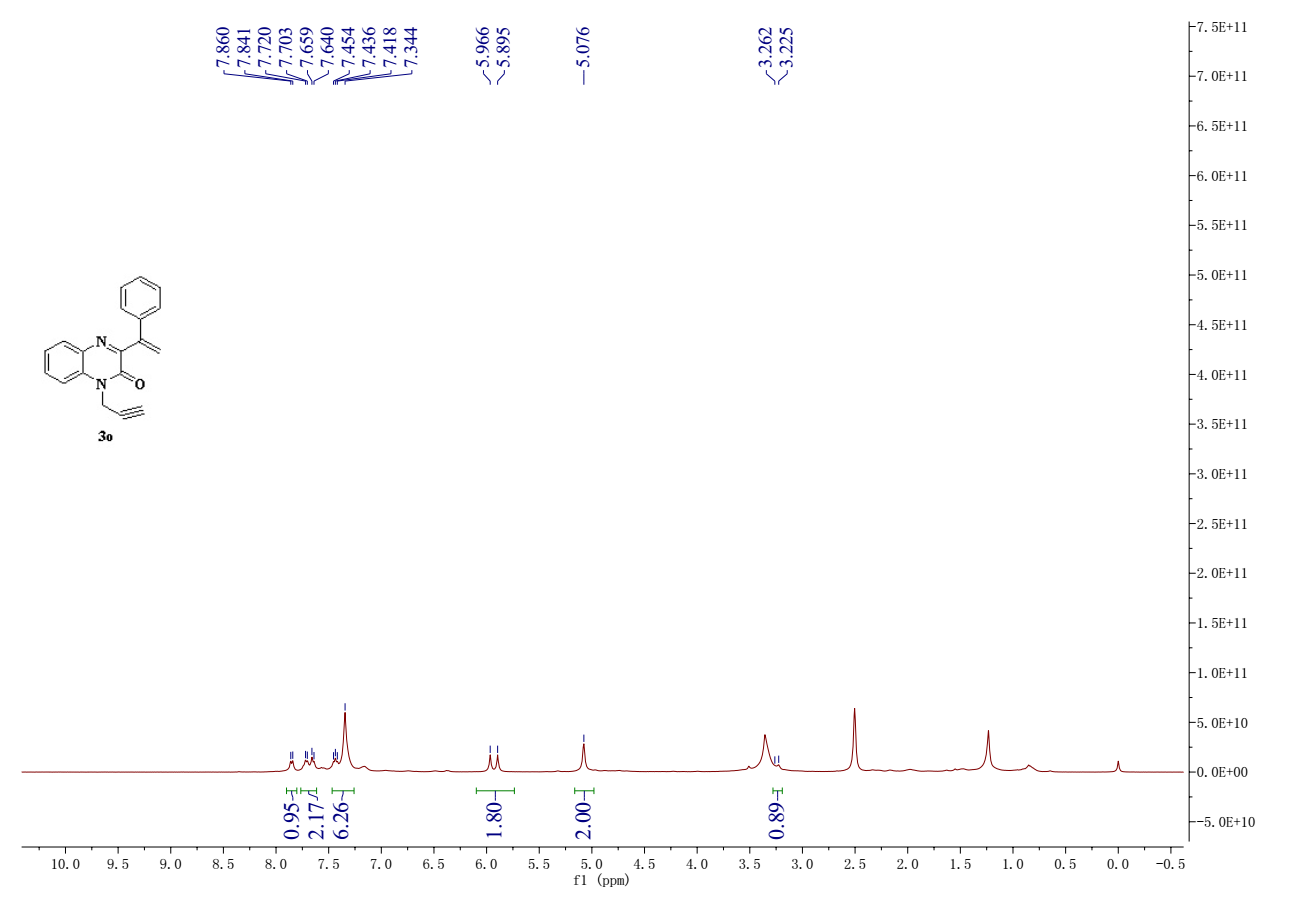


^1^H NMR spectrum of compound **3o** (400 MHz, DMSO-*d*_6_)


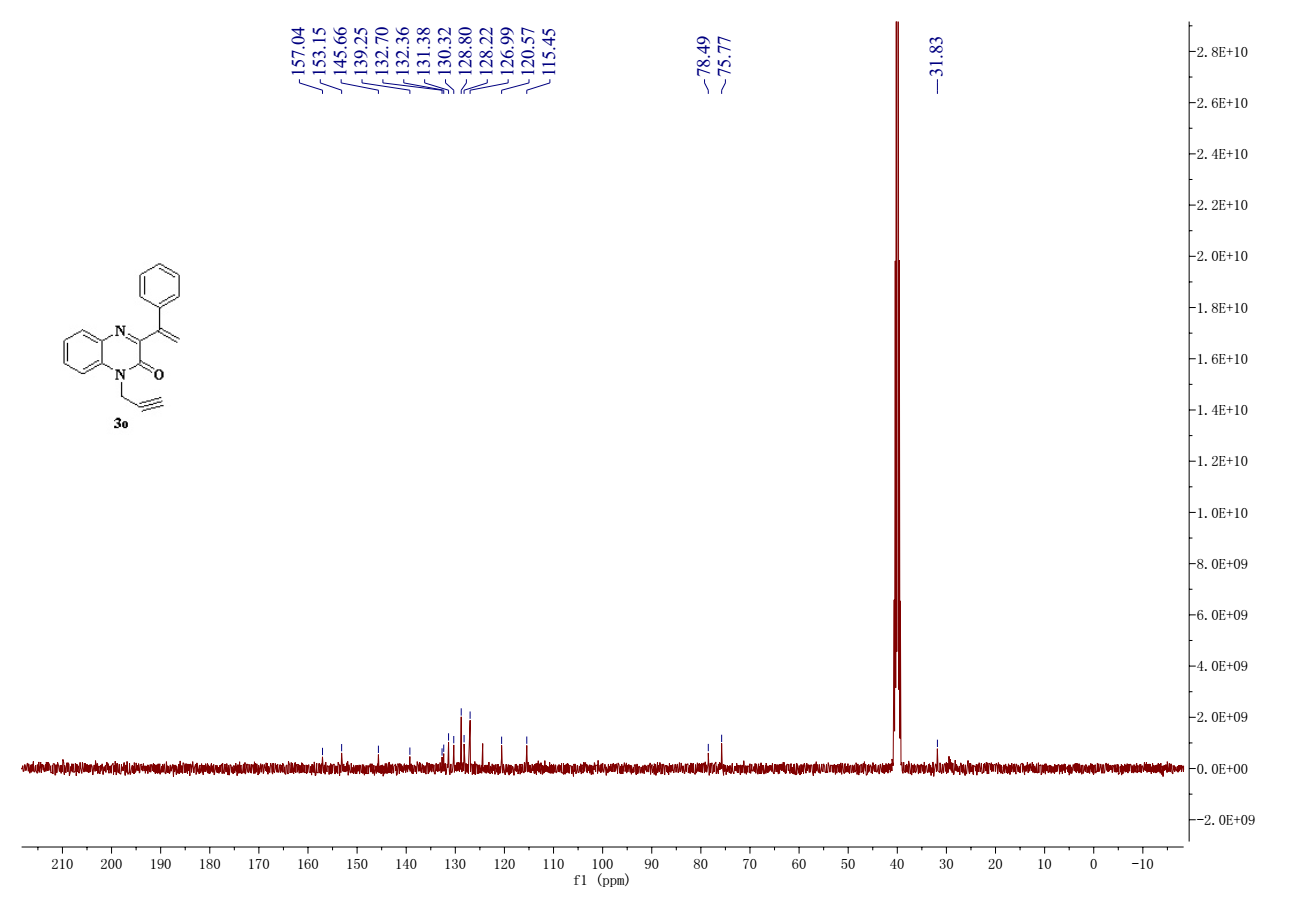


^13^C NMR spectrum of compound **3o** (100 MHz, DMSO-*d*_6_)


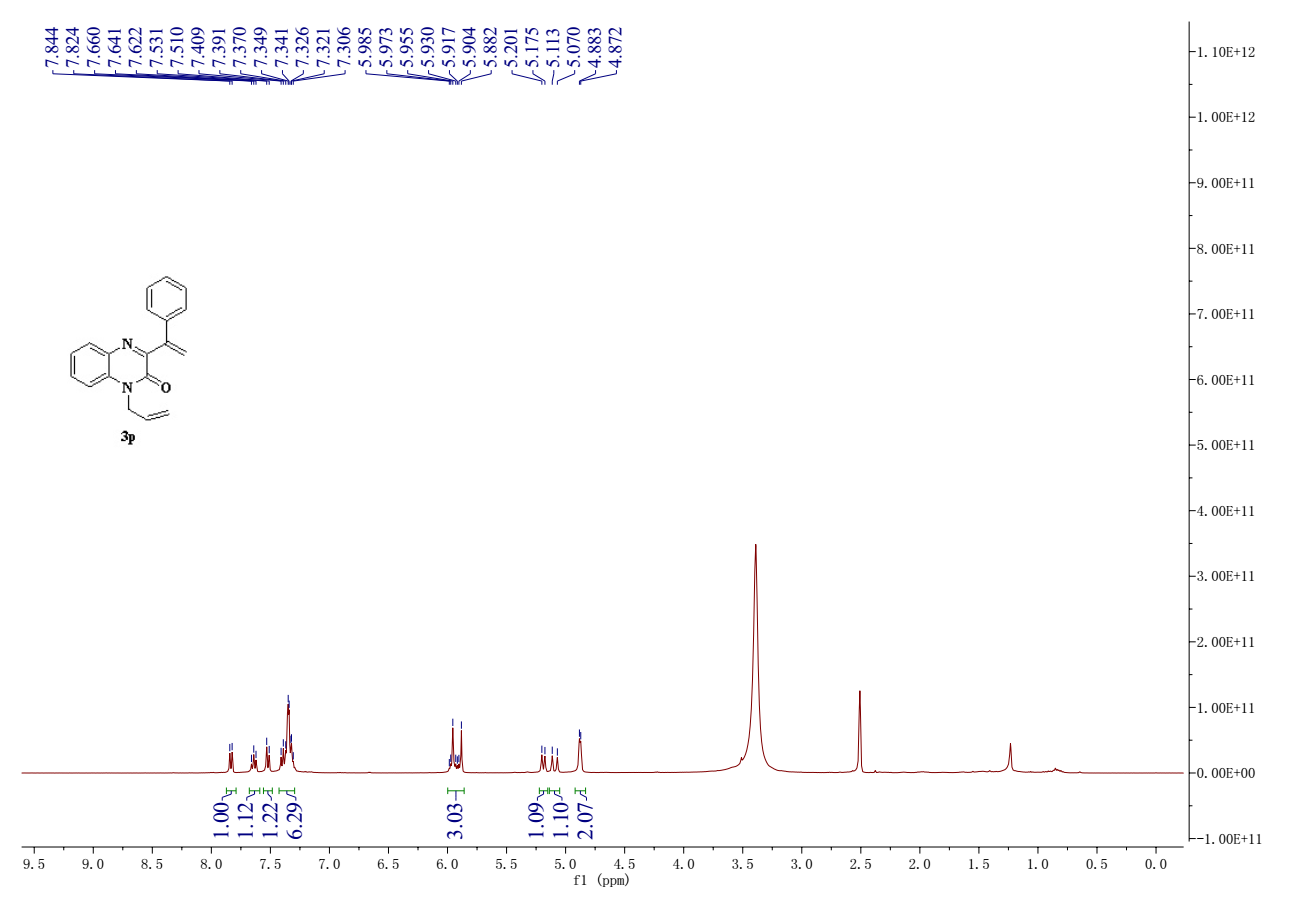


^1^H NMR spectrum of compound **3p** (400 MHz, DMSO-*d*_6_)


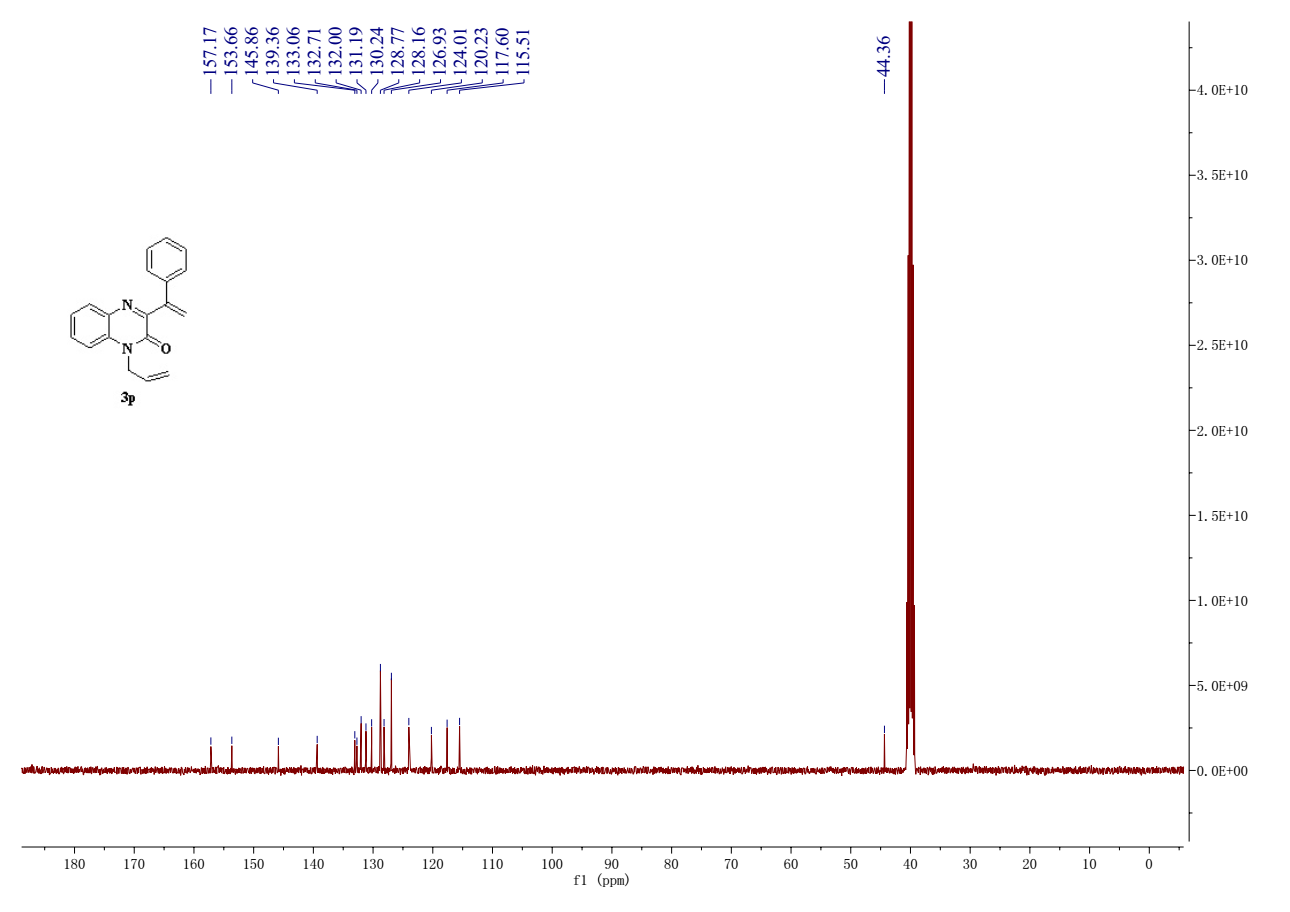


^13^C NMR spectrum of compound **3p** (100 MHz, DMSO-*d*_6_)


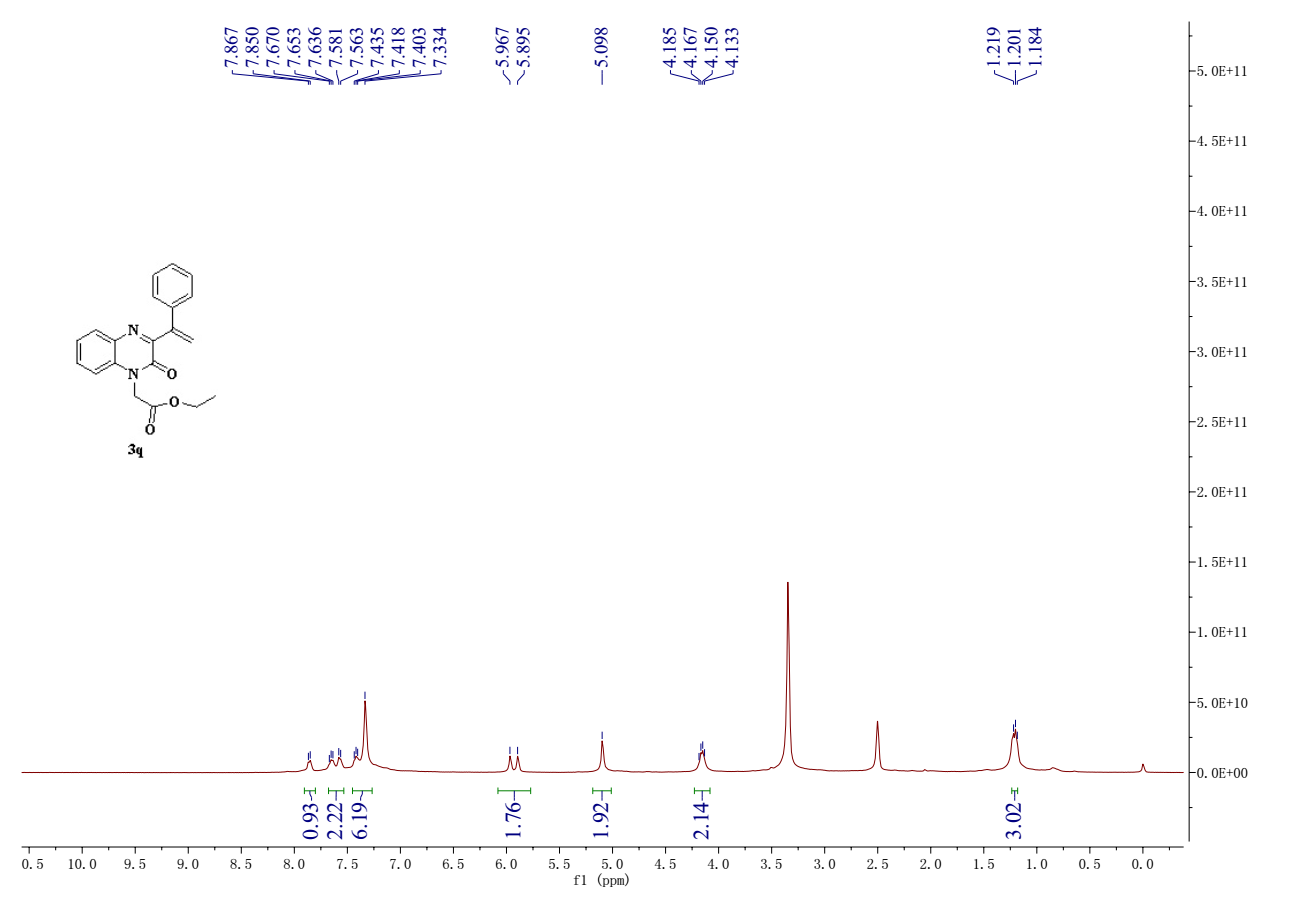


^1^H NMR spectrum of compound **3q** (400 MHz, DMSO-*d*_6_)


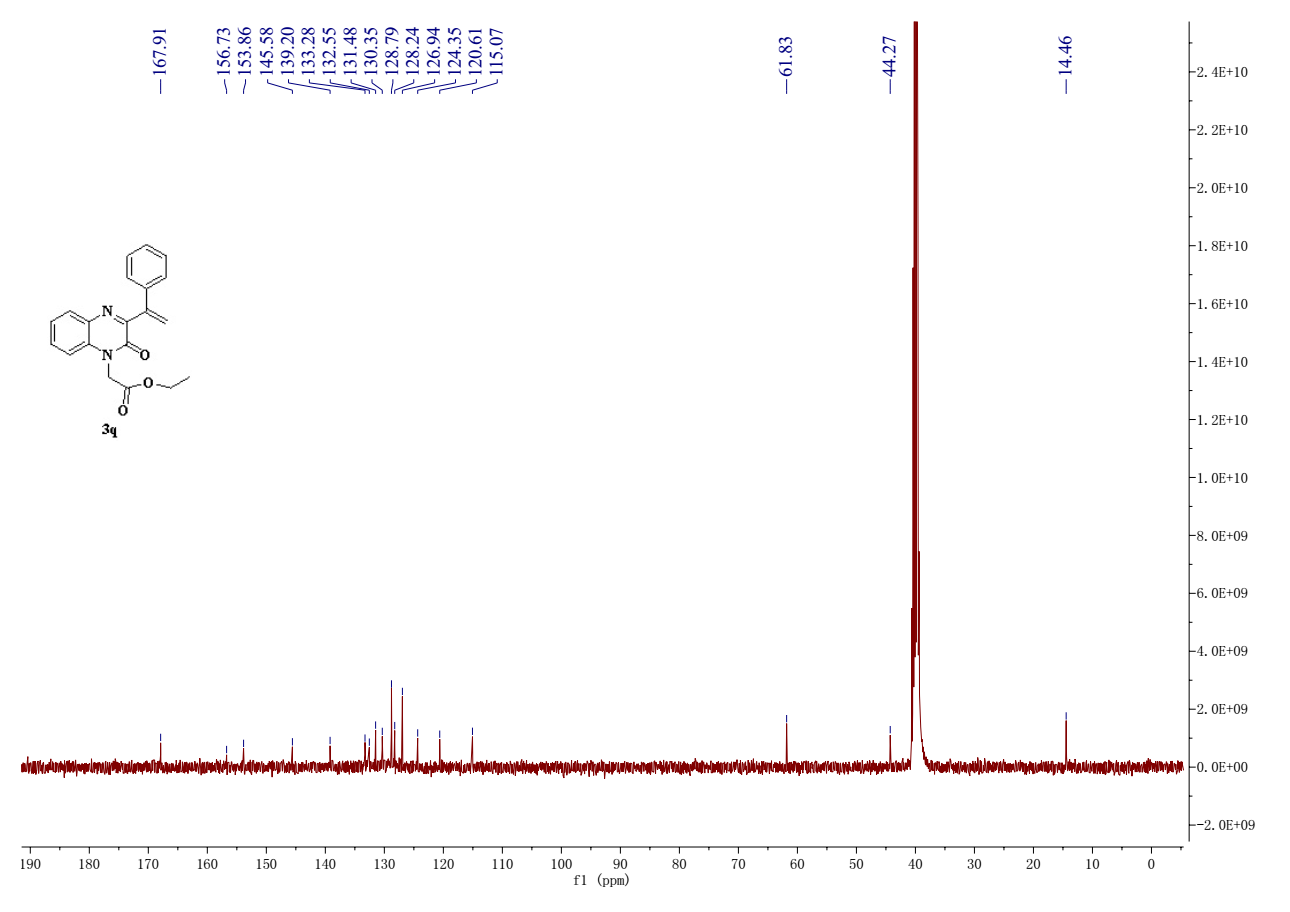


^13^C NMR spectrum of compound **3q**(100 MHz, DMSO-*d*_6_)


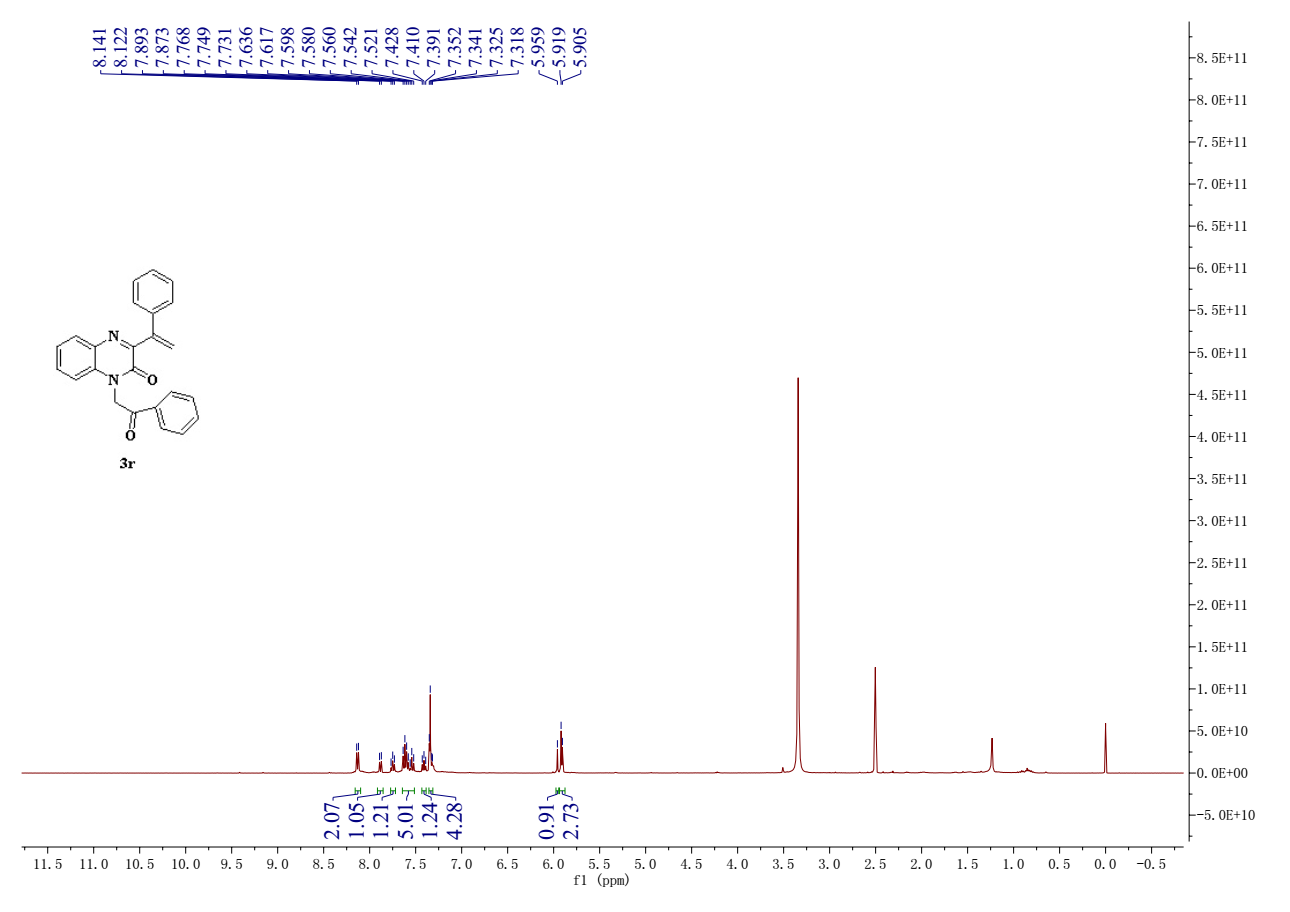


^1^H NMR spectrum of compound **3r** (400 MHz, DMSO-*d*_6_)


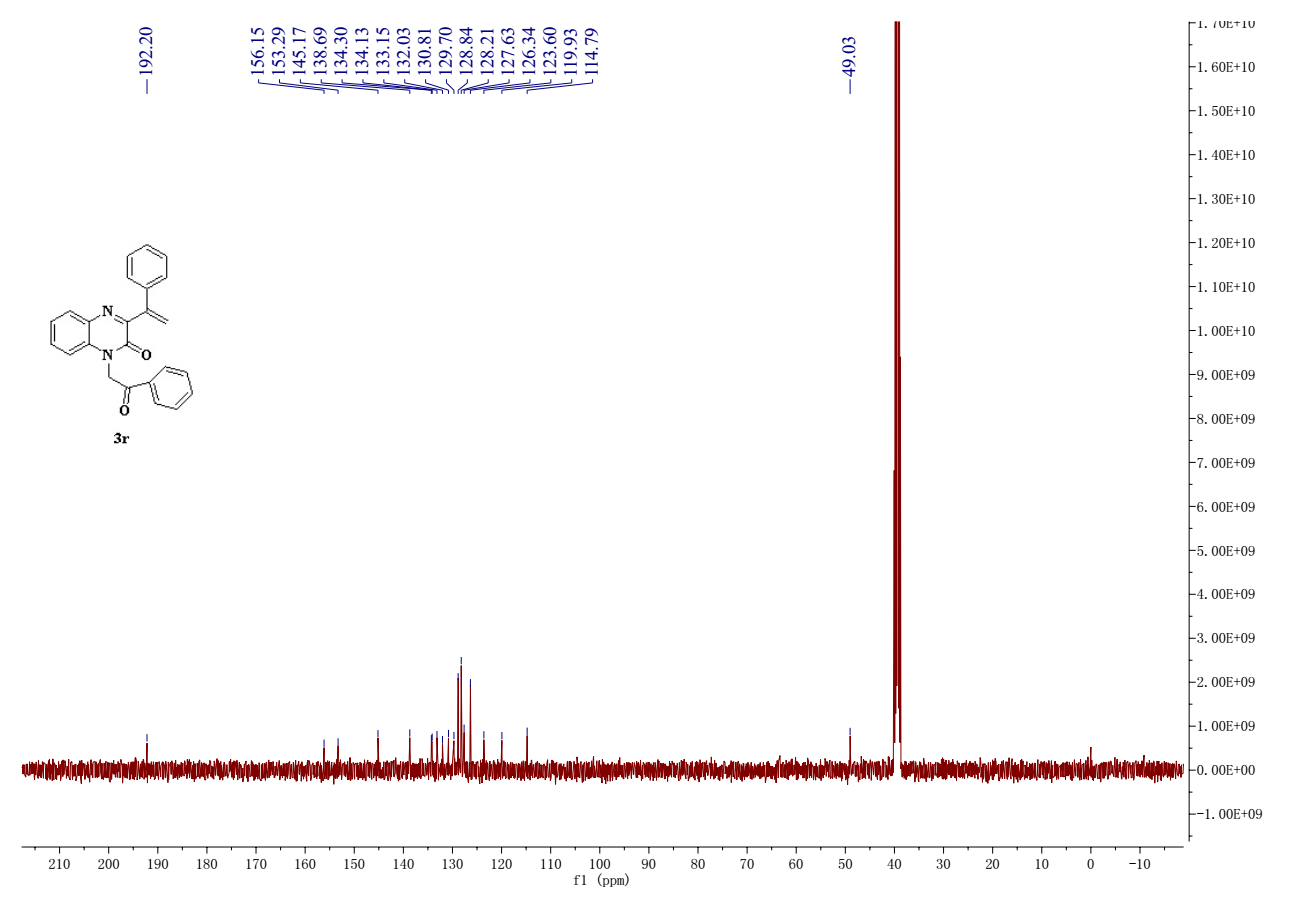


^13^C NMR spectrum of compound **3r** (100 MHz, DMSO-*d*_6_)


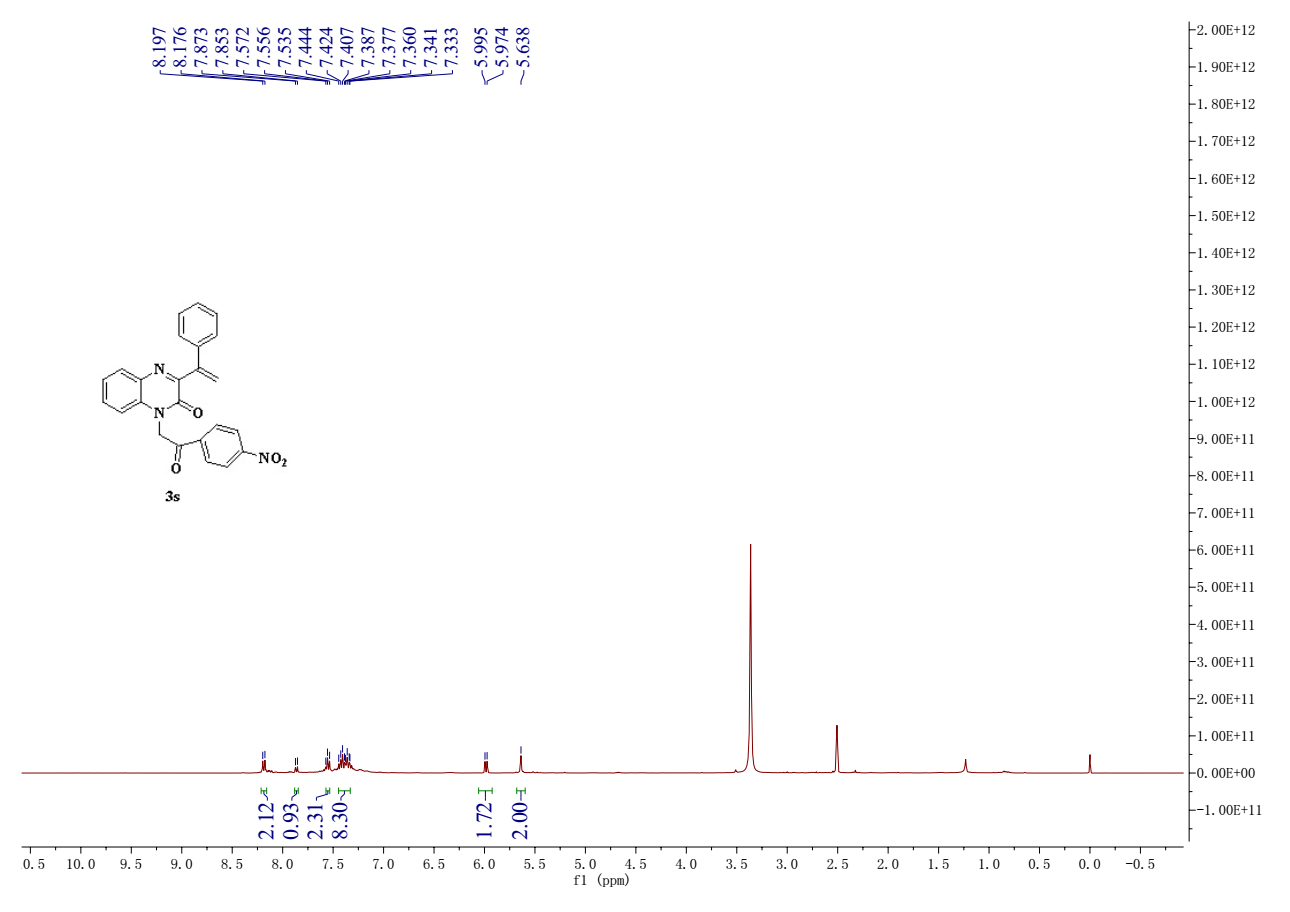


^1^H NMR spectrum of compound **3s** (400 MHz, DMSO-*d*_6_)


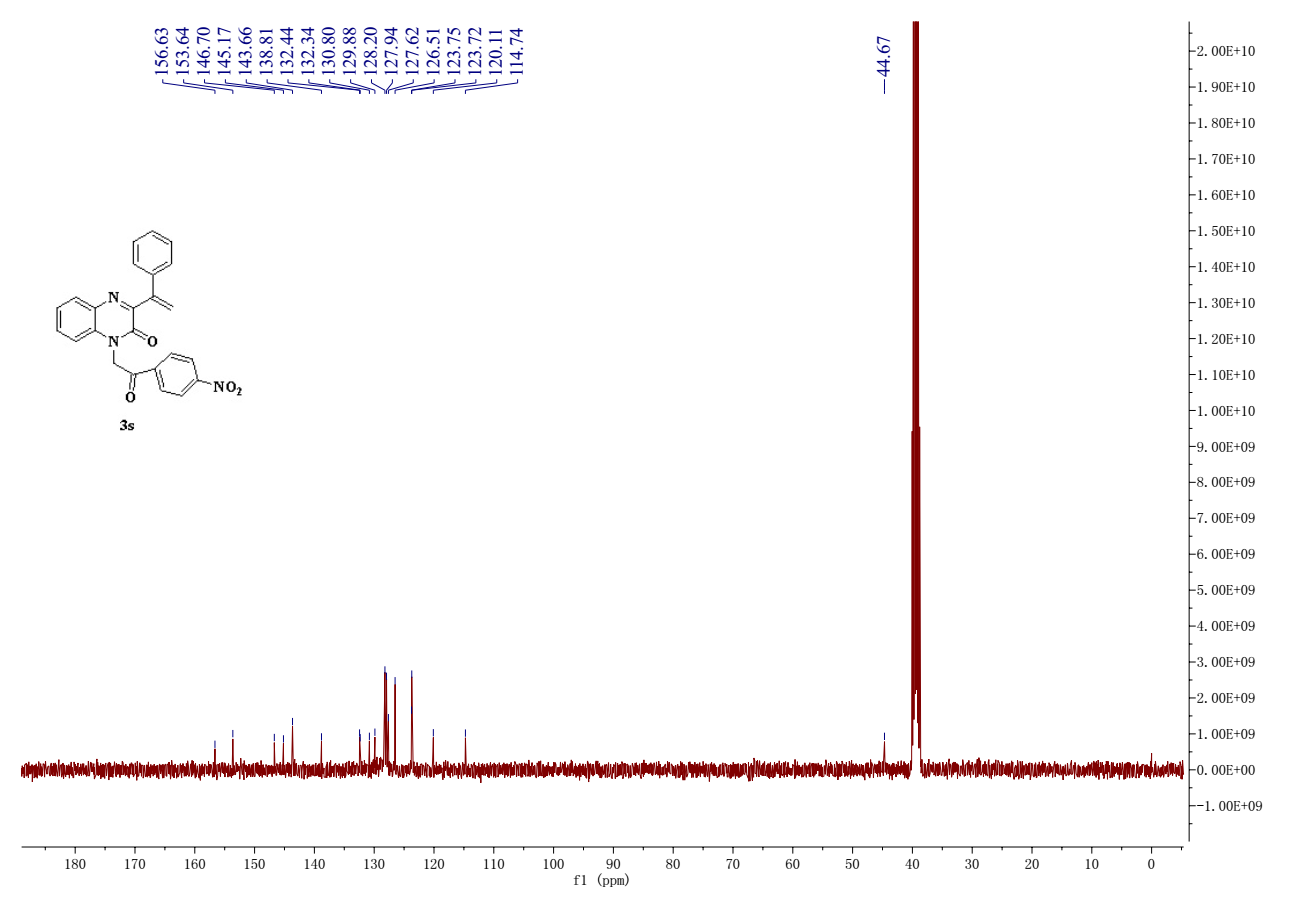


^13^C NMR spectrum of compound **3s** (100 MHz, DMSO-*d*_6_)


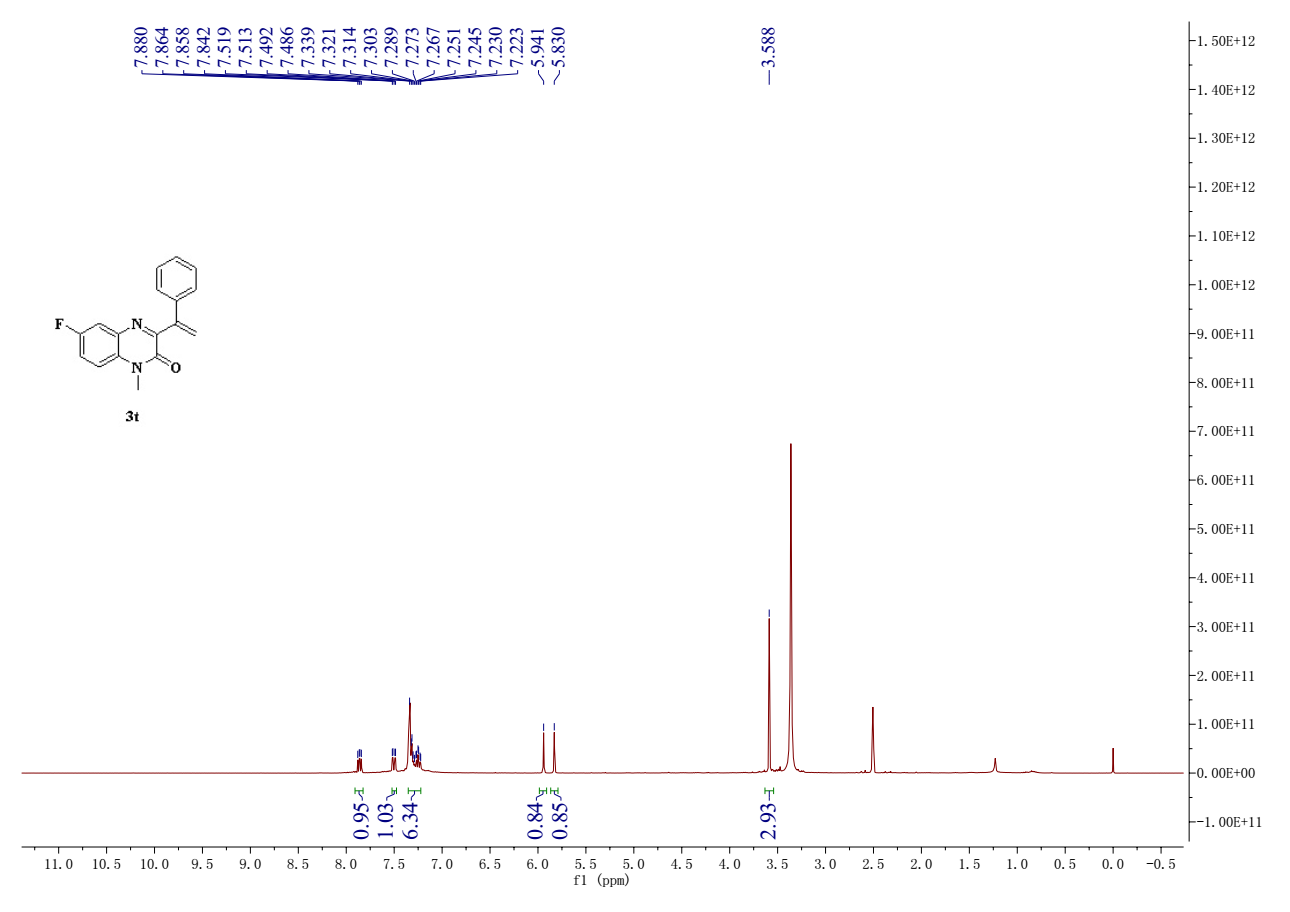


^1^H NMR spectrum of compound **3t** (400 MHz, DMSO-*d*_6_)


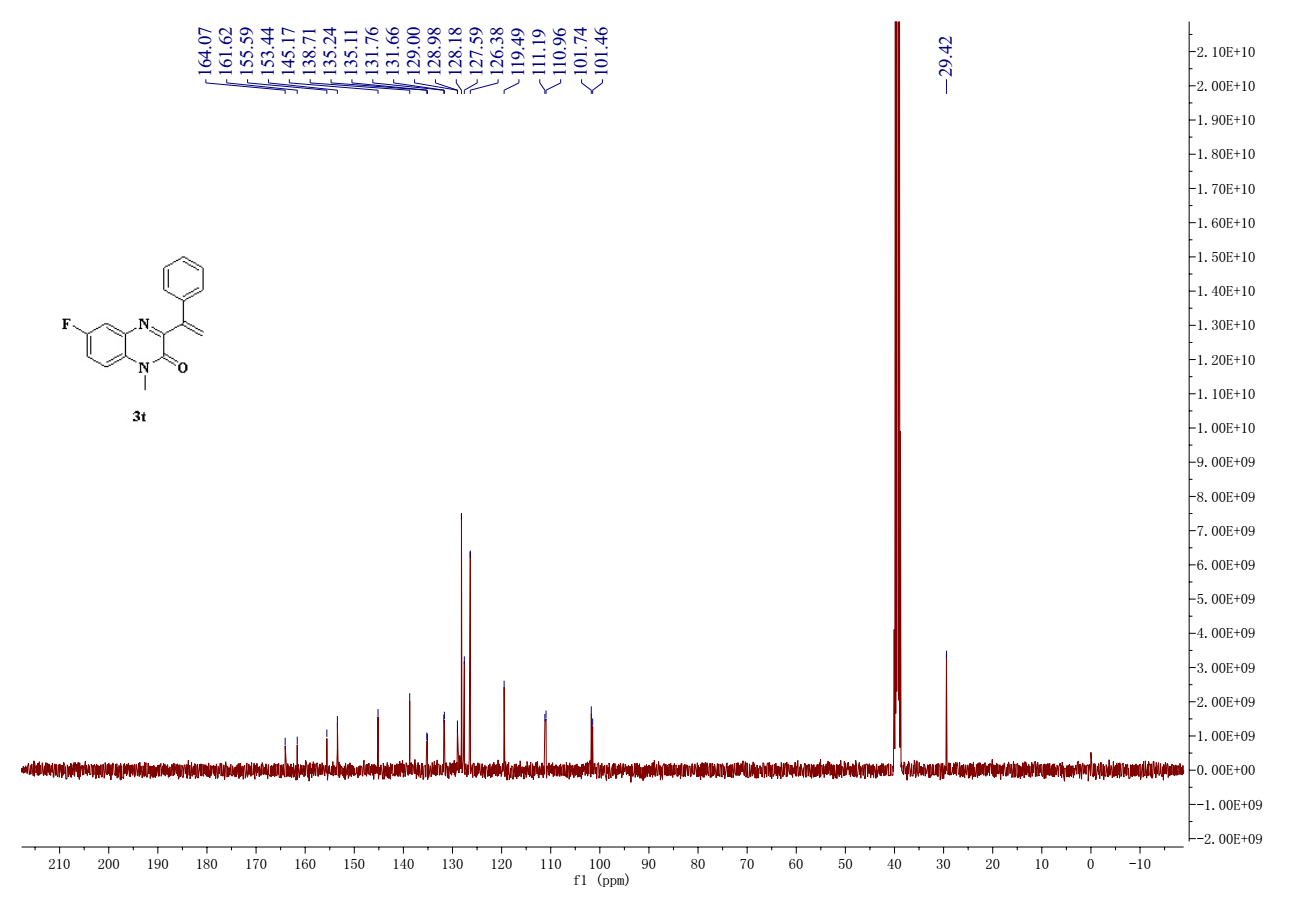


^13^C NMR spectrum of compound **3t** (100 MHz, DMSO-*d*_6_)


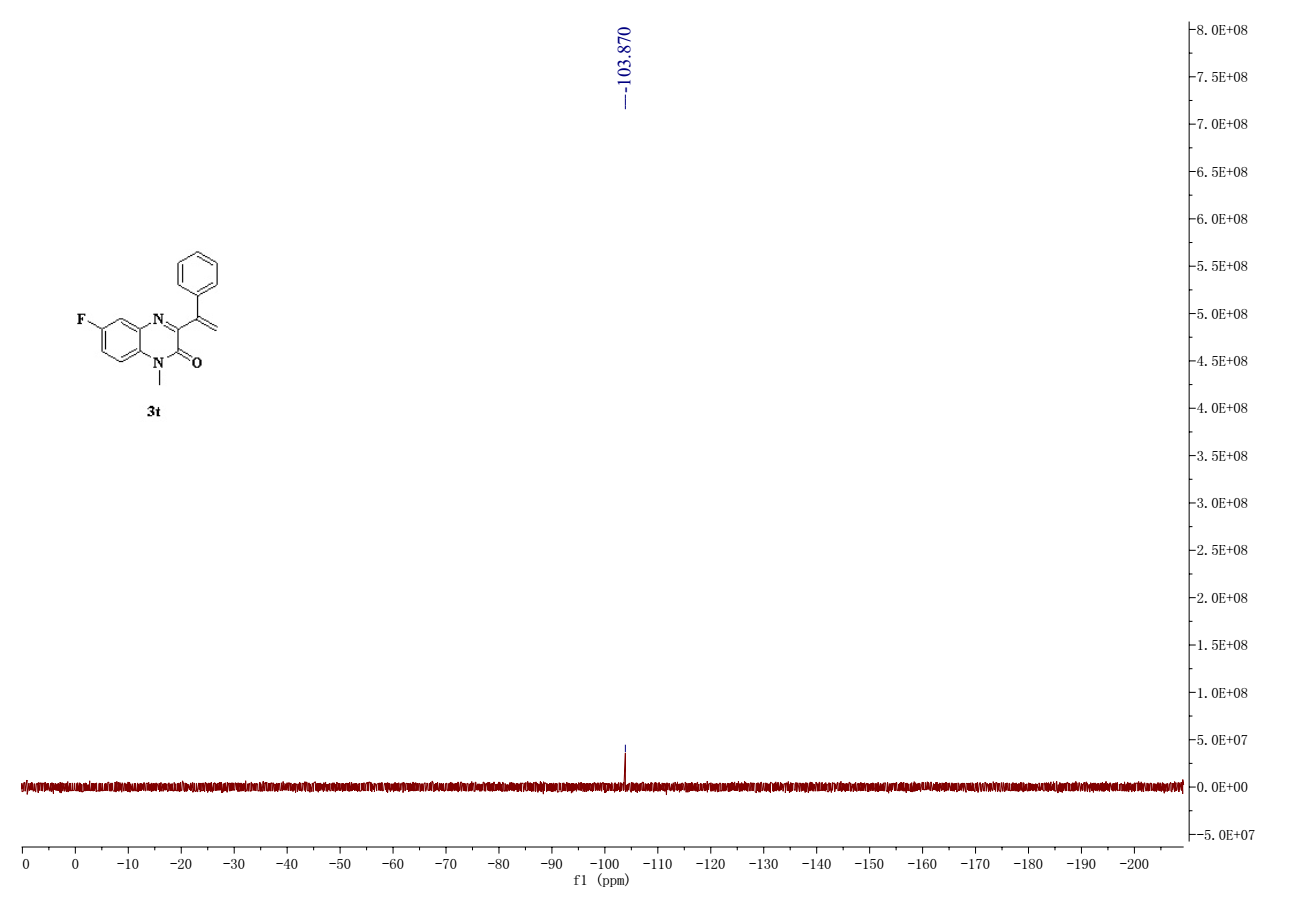


^19^F NMR spectrum of compound **3t** (377 MHz, DMSO-*d*_6_)


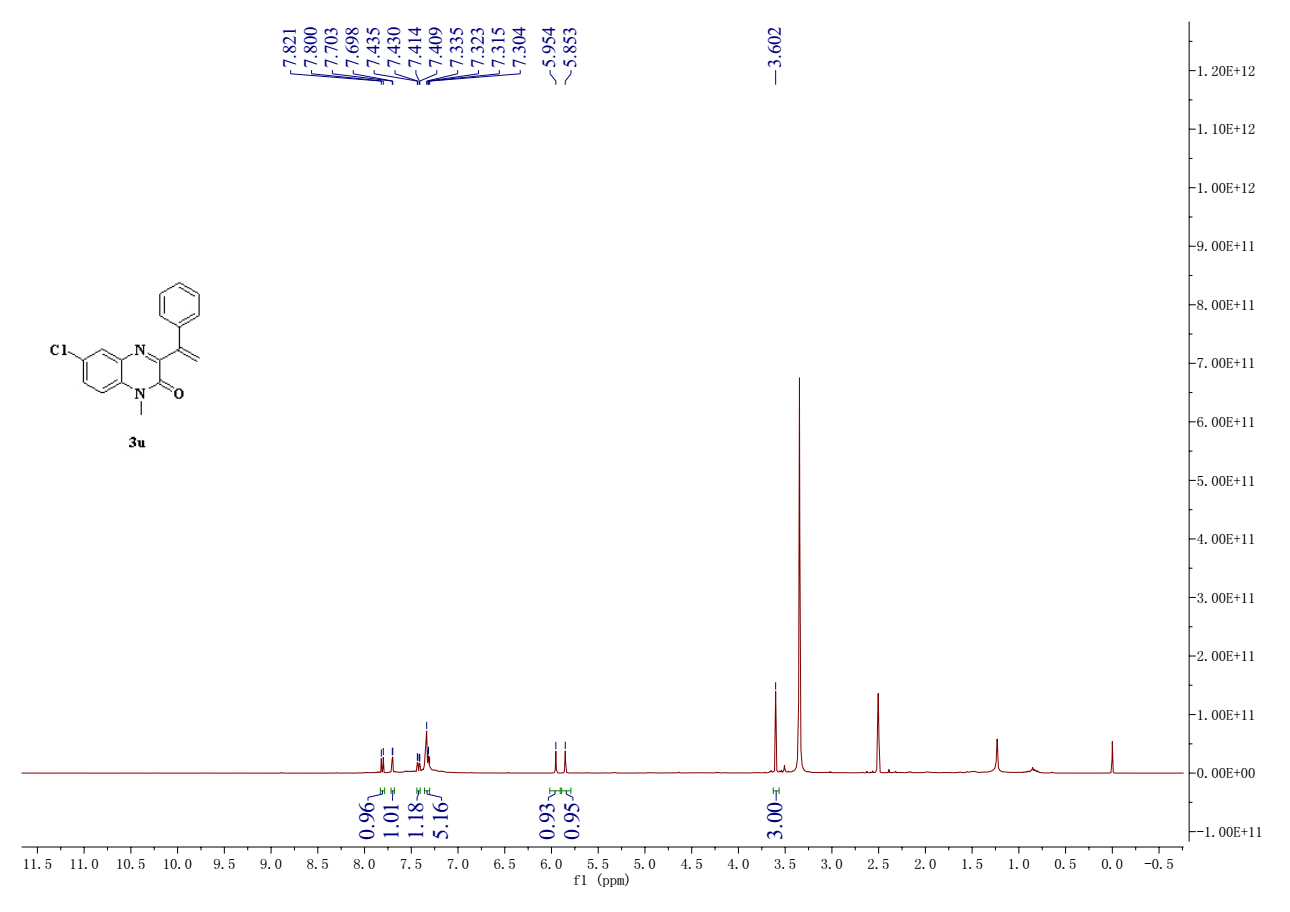


^1^H NMR spectrum of compound **3u** (400 MHz, DMSO-*d*_6_)


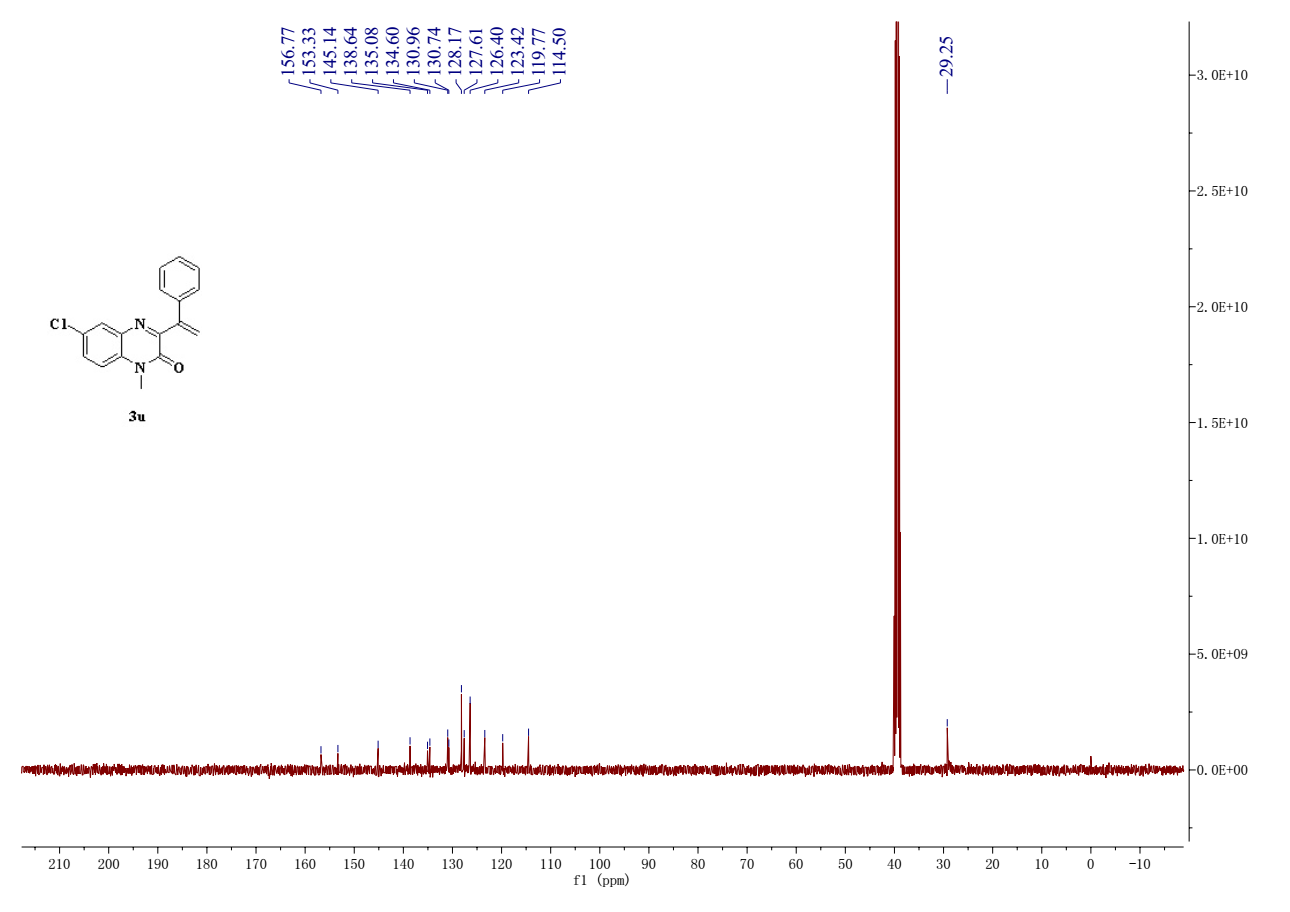


^13^C NMR spectrum of compound **3u** (100 MHz, DMSO-*d*_6_)


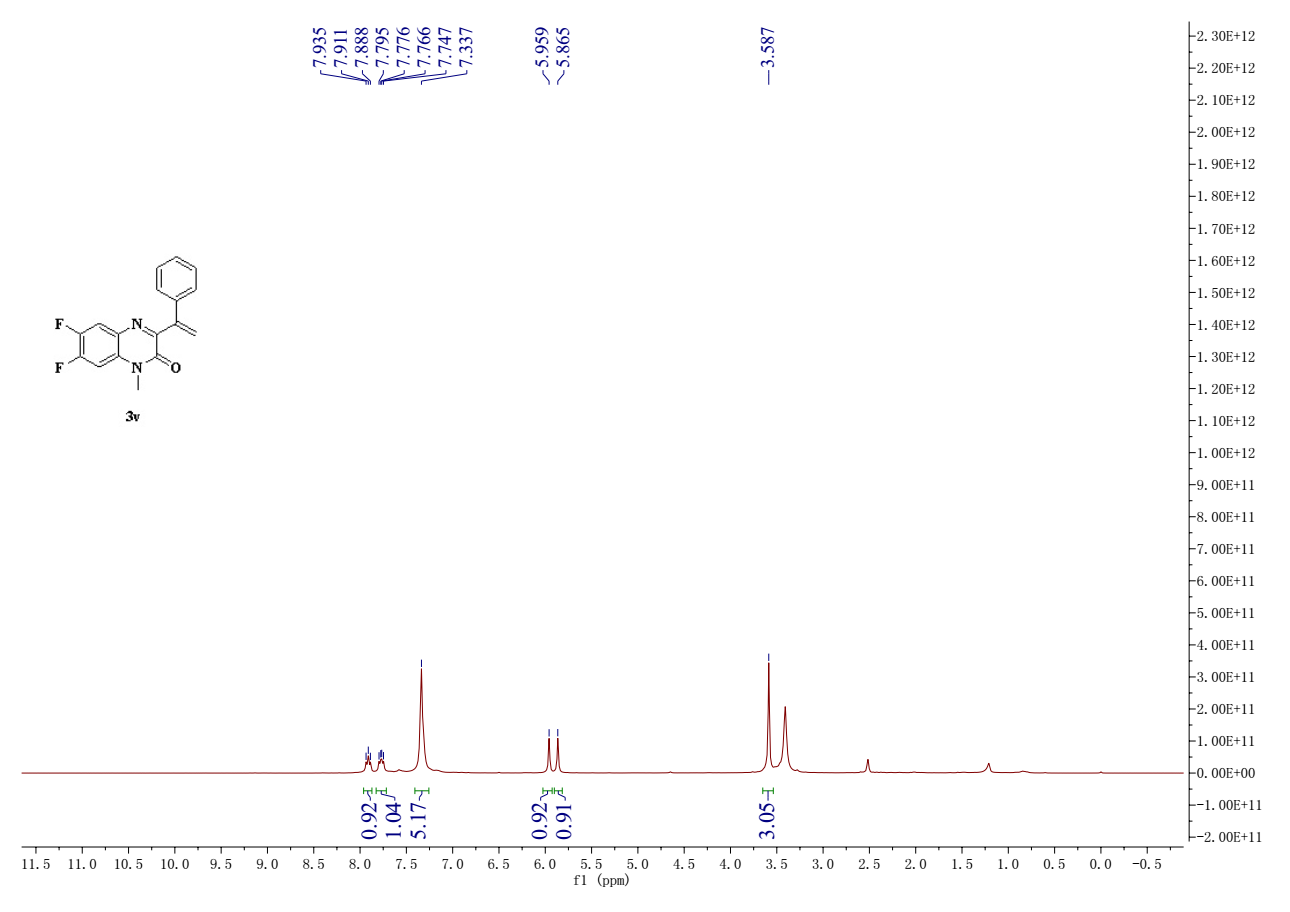


^1^H NMR spectrum of compound **3v** (400 MHz, DMSO-*d*_6_)


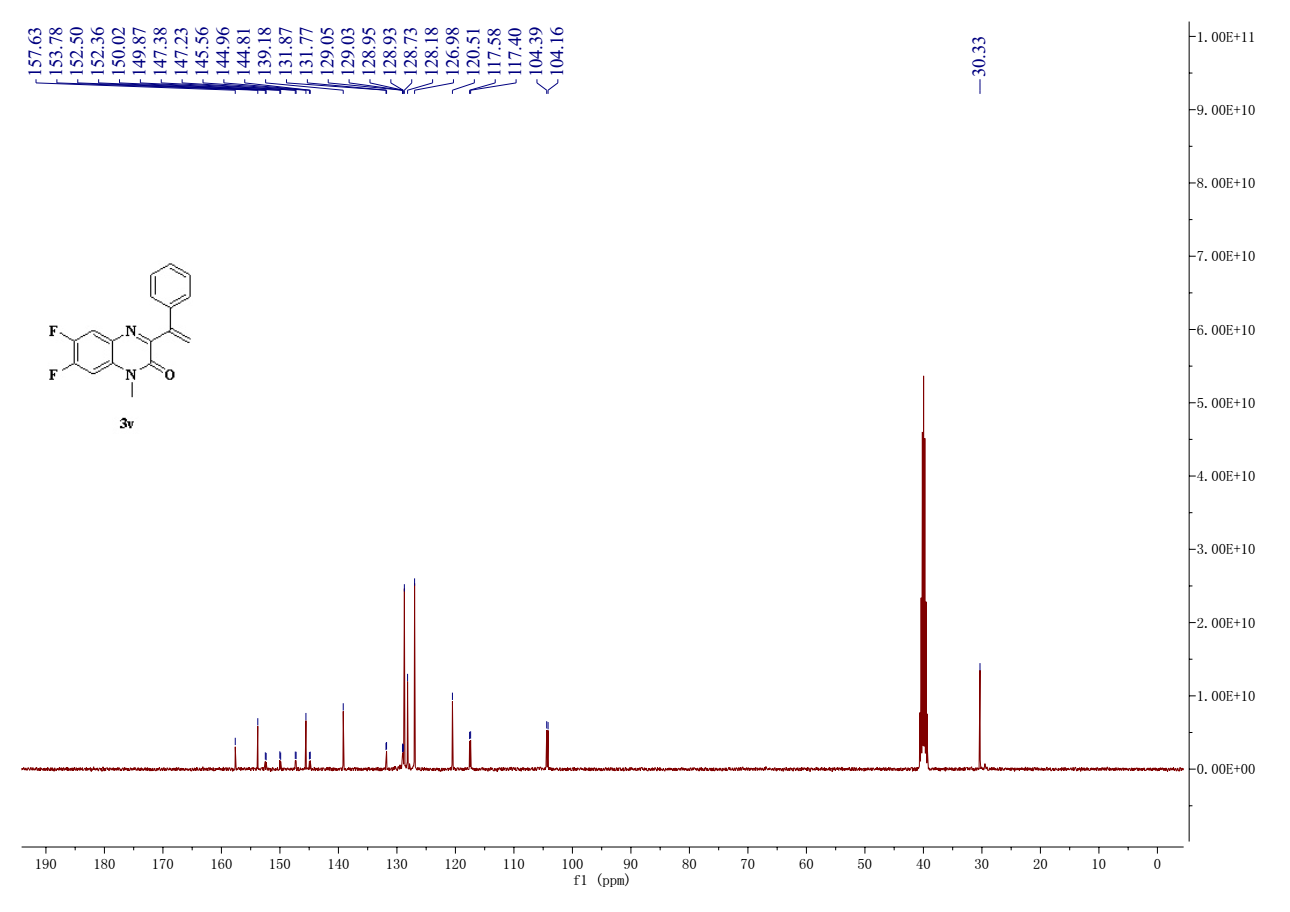


^13^C NMR spectrum of compound **3v** (100 MHz, DMSO-*d*_6_)


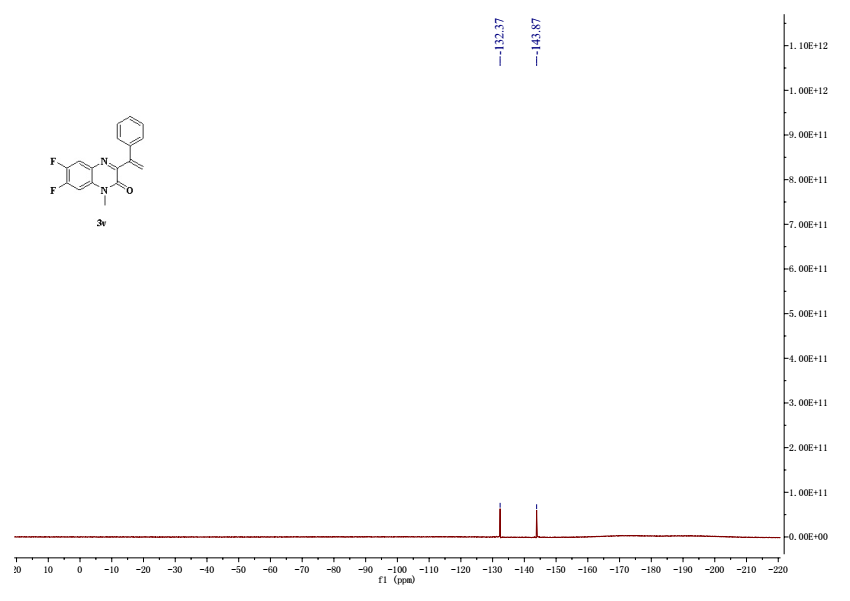


^19^F NMR spectrum of compound **3v** (377 MHz, DMSO-*d*_6_)


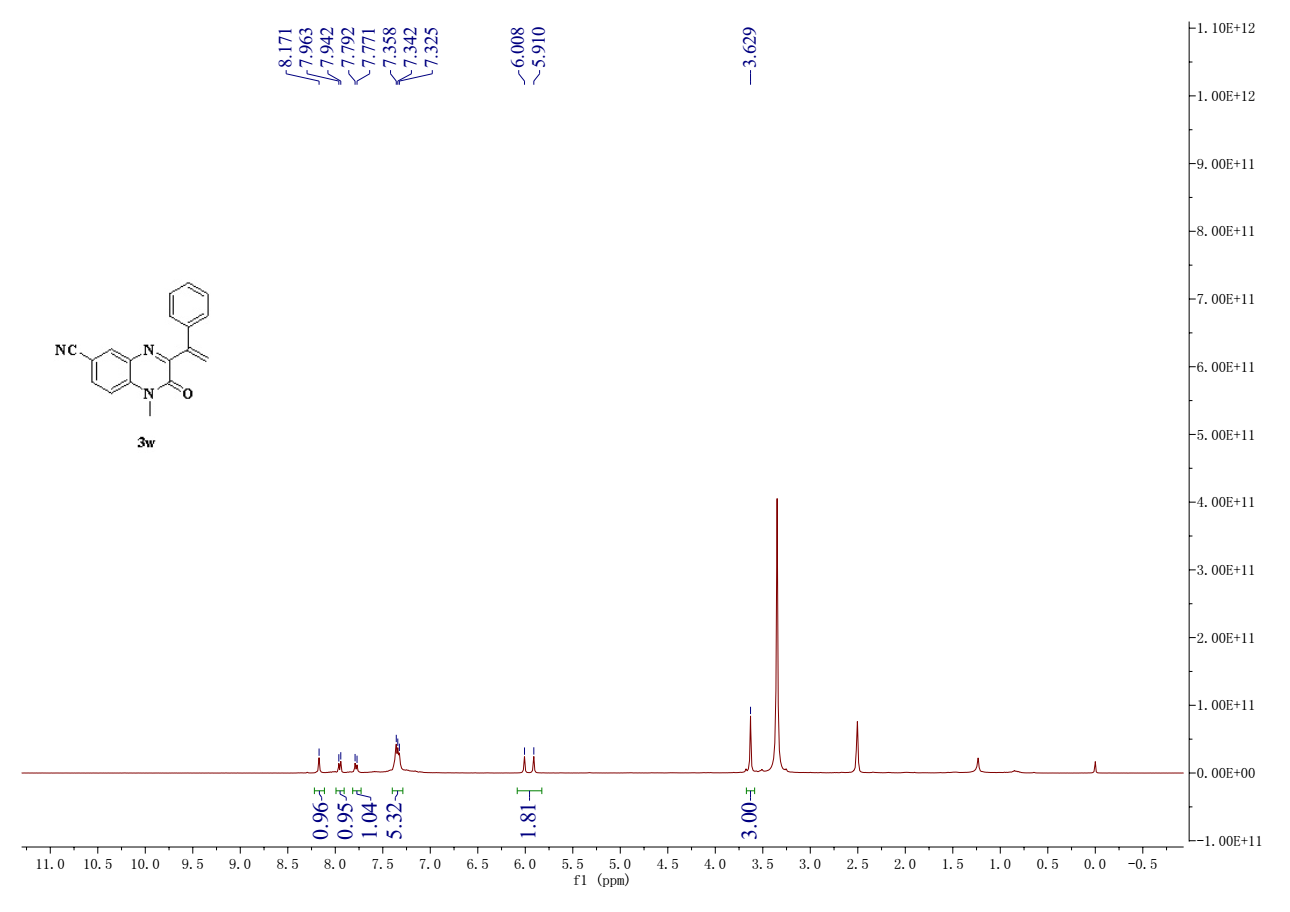


^1^H NMR spectrum of compound **3w** (400 MHz, DMSO-*d*_6_)


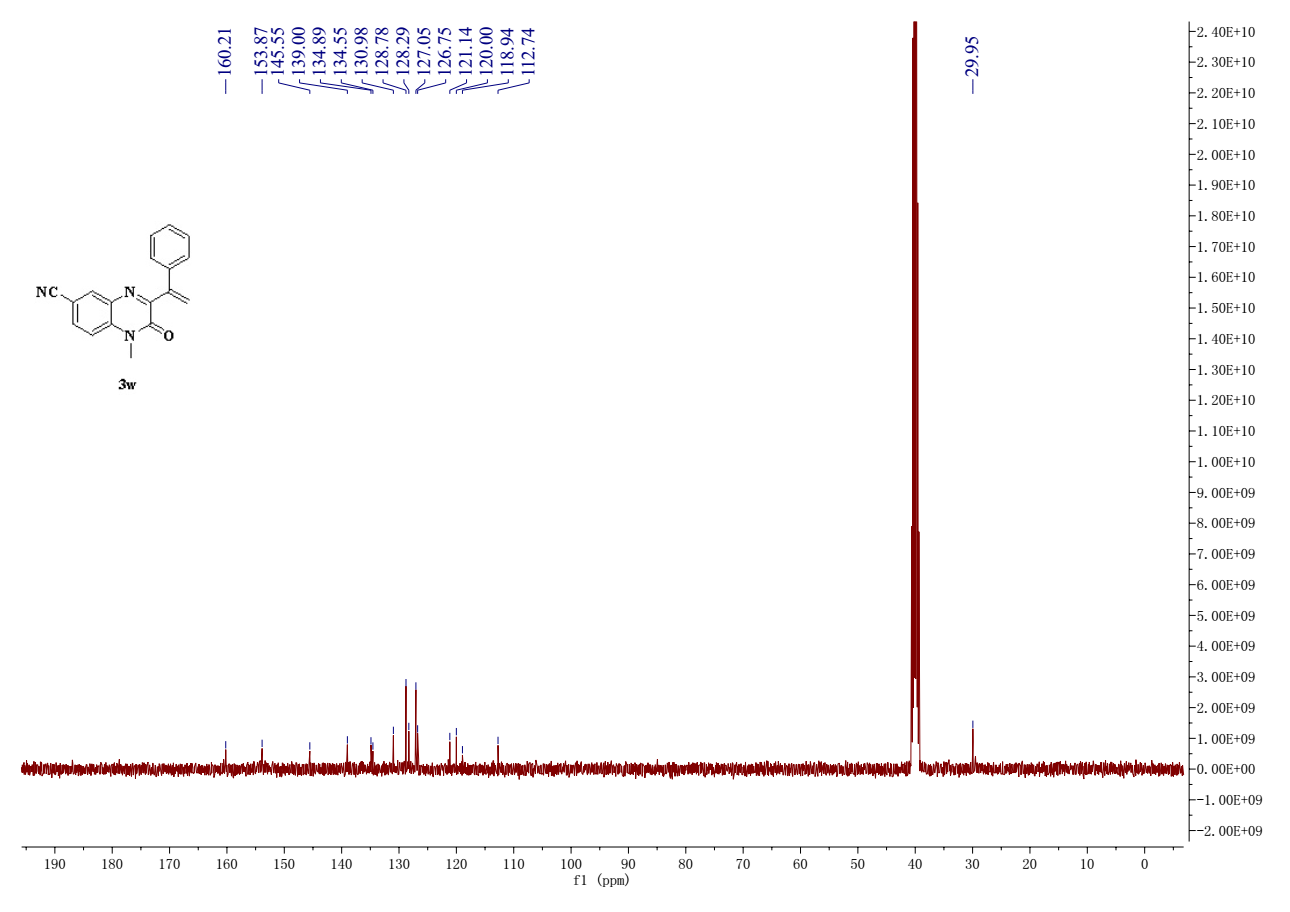


^13^C NMR spectrum of compound **3w** (100 MHz, DMSO-*d*_6_)


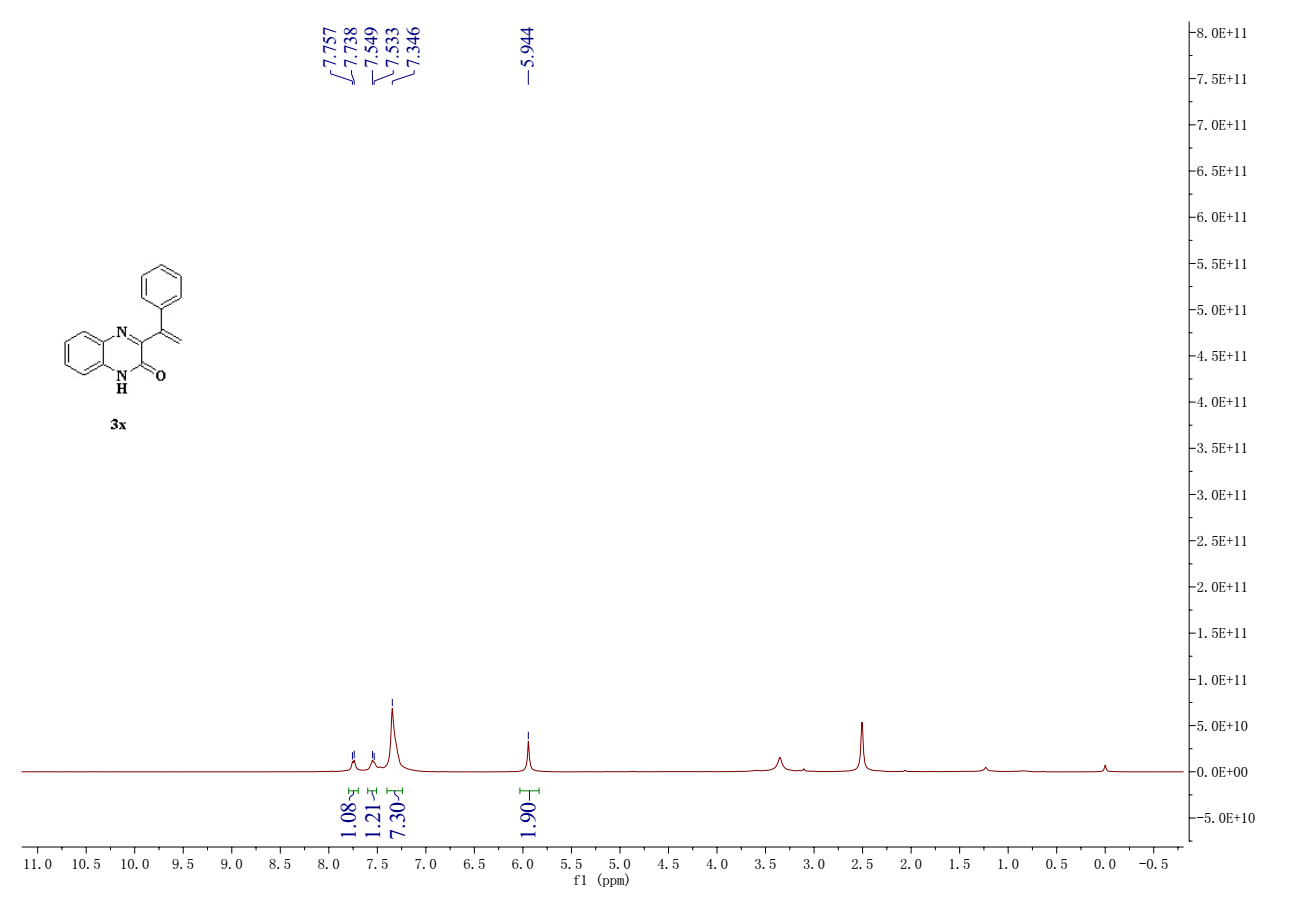


^1^H NMR spectrum of compound **3x** (400 MHz, DMSO-*d*_6_)


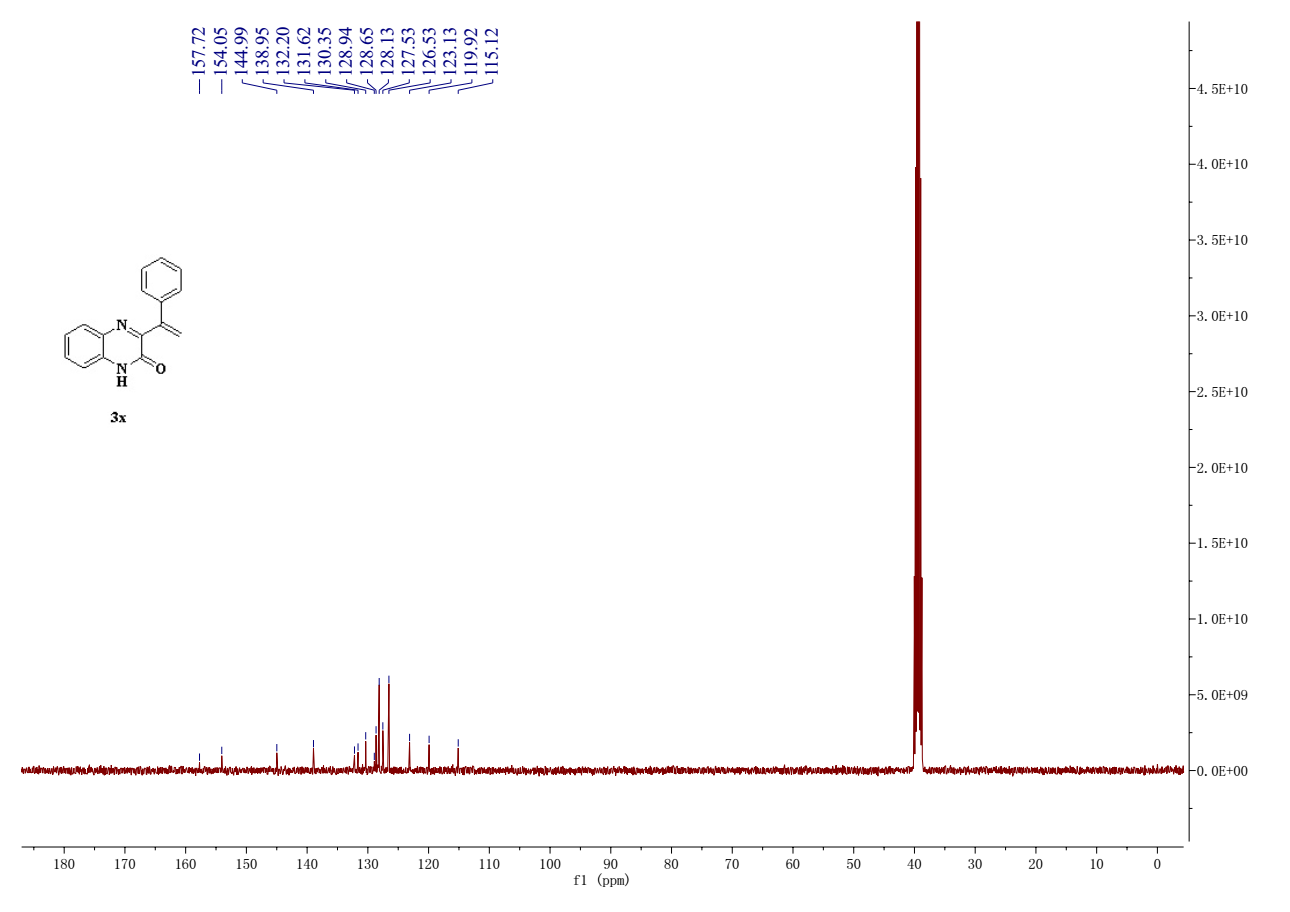


^13^C NMR spectrum of compound **3x** (100 MHz, DMSO-*d*_6_)


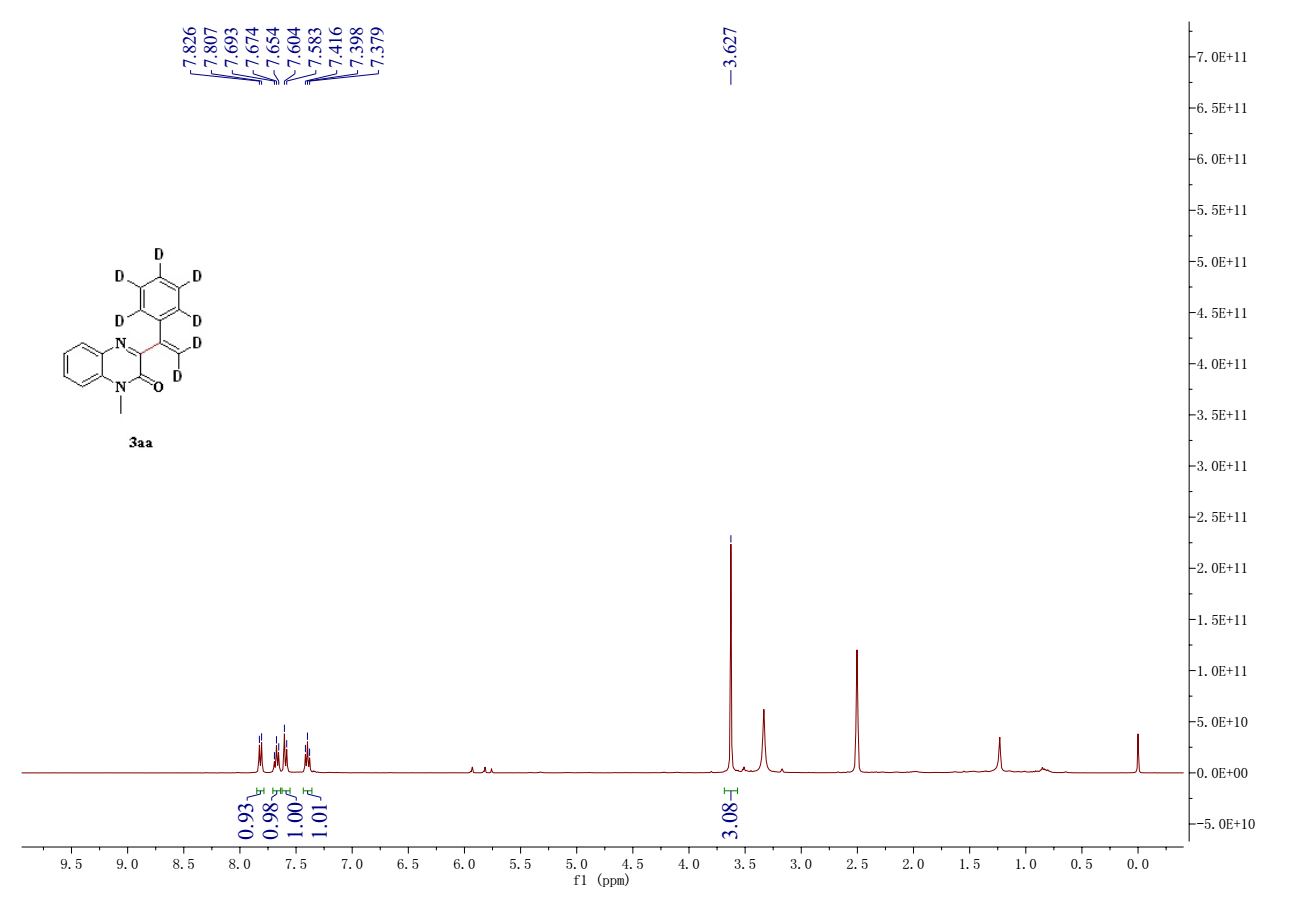


^1^H NMR spectrum of compound **3aa** (400 MHz, DMSO-*d*_6_)


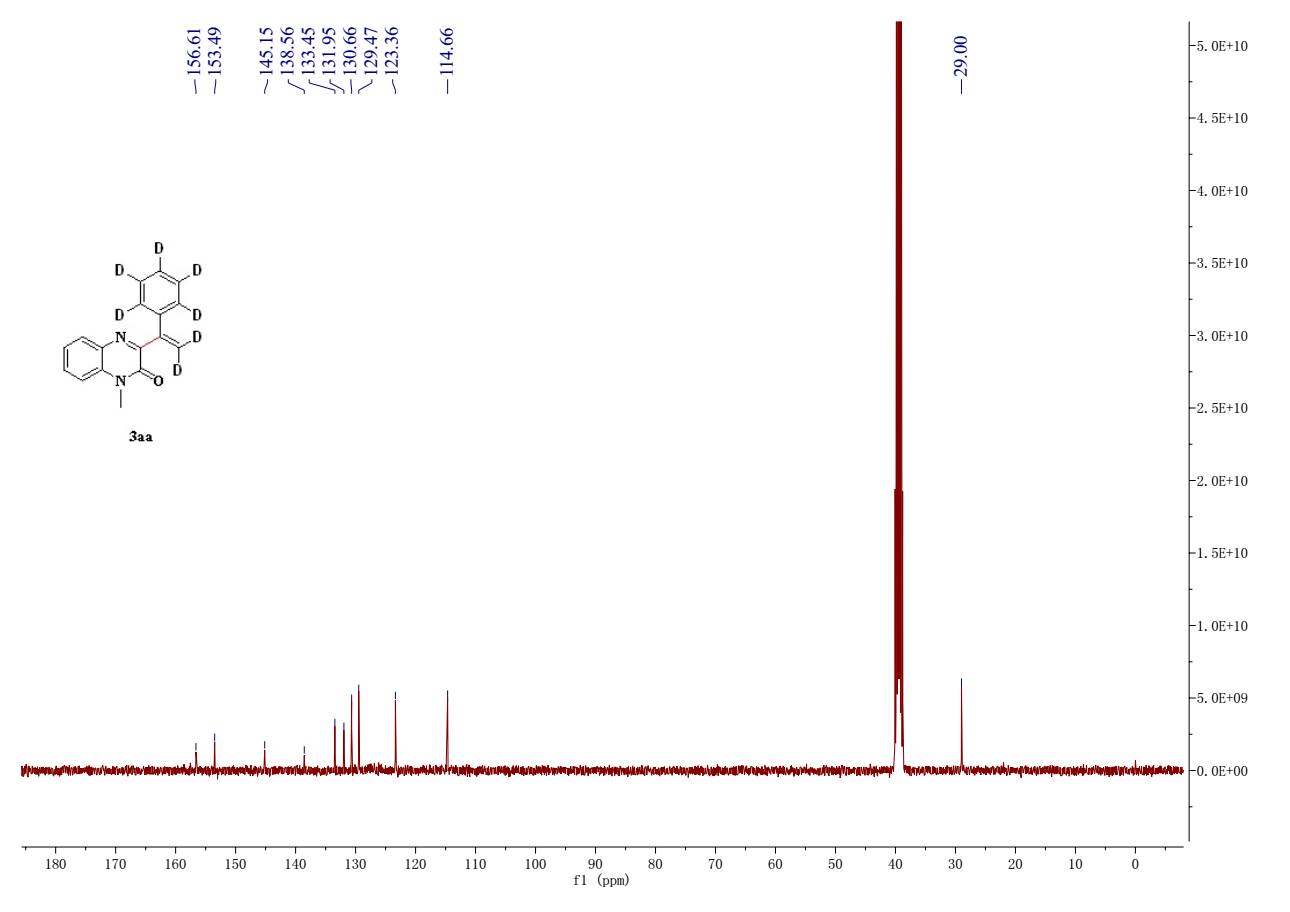


^13^C NMR spectrum of compound **3aa** (100 MHz, DMSO-*d*_6_)


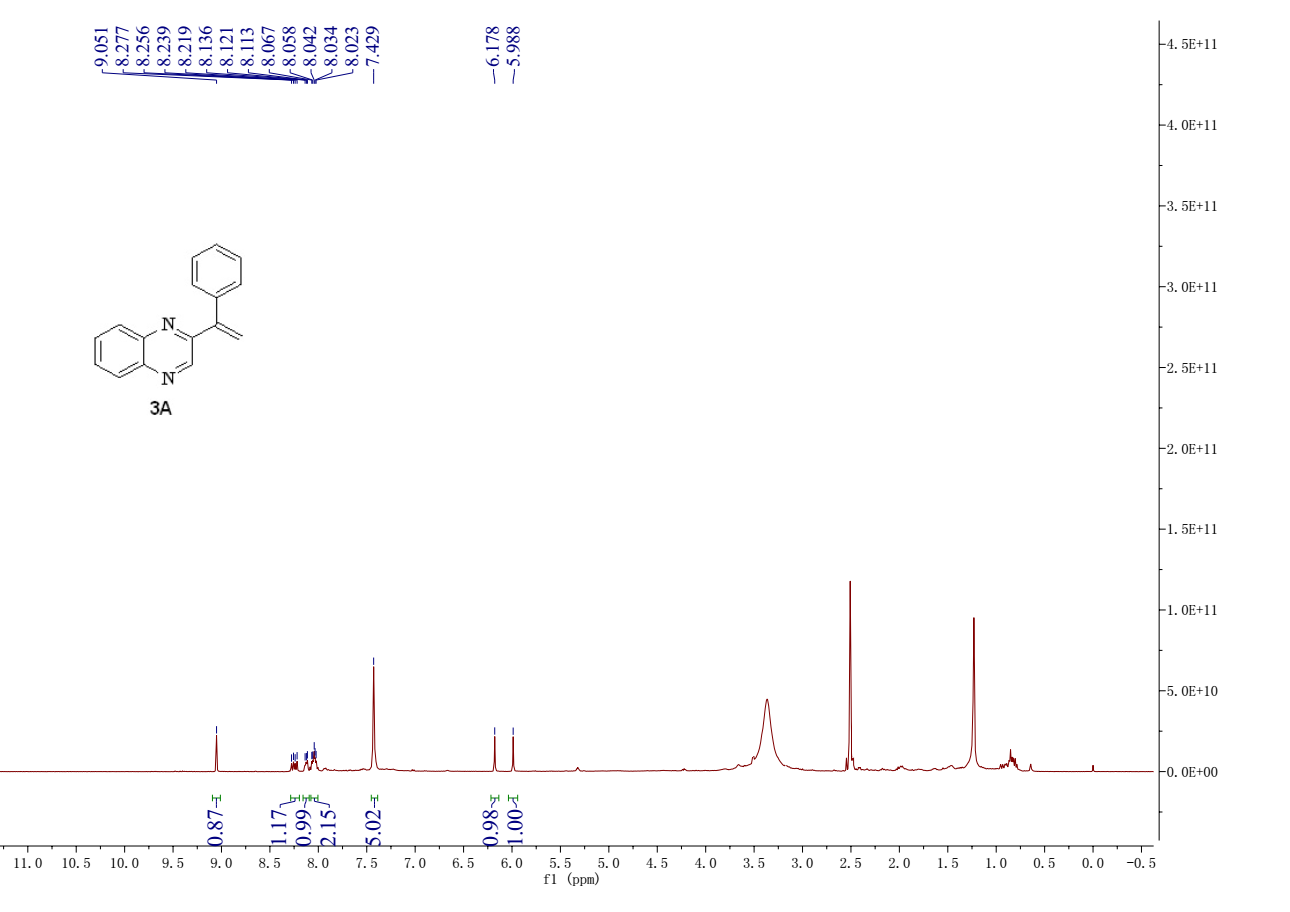


^1^H NMR spectrum of compound **3A** (400 MHz, DMSO-*d*_6_)


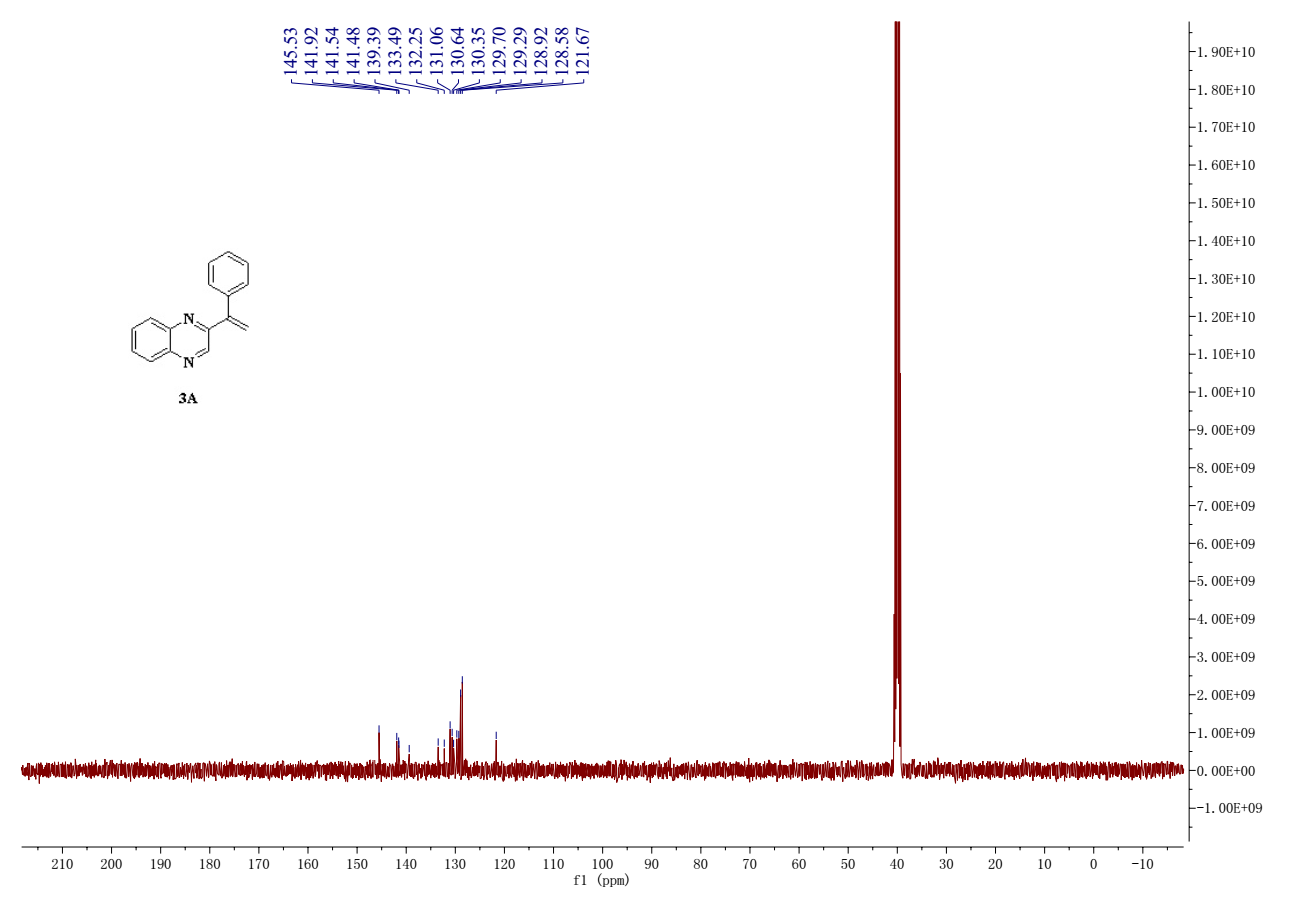


^13^C NMR spectrum of compound **3A**(100 MHz, DMSO-*d*_6_)

**Ⅳ. Reference**:

[1] Rawat, D., Kumar, R., Subbarayappa, A. (2020)Visible-light induced phosphonation of quinoxalines and quinoxalin-2(1*H*)-ones under aerobic metal-free conditions. *Green Chemistry.* 22, 6170-6175. doi: 10.1039/D0GC02168G

[2] Niu, K., Song, L., Hao, Y., Liu, Y., Wang, Q. *(*2020)Electrochemical decarboxylative C3 alkylation of quinoxalin-2(1*H*)-ones with N-hydroxyphthalimide esters. *Chem Commun.* 56, 11673-11676. doi: 10.1039/D0CC05391K

[3] Ni, H., Shi, X., Li, Y., Zhang, X., Zhao, J., Zhao, F. (2020)Metal-free C3–H acylation of quinoxalin-2(1*H*)-ones with α-oxo-carboxylic acids*Org Biomol Chem.* 18,6558-6563. doi: 10.1039/D0OB01423K

[4] Carrer, A., Brion, J., Messaoudi, S., Alami, M.(2013) Palladium(II)-Catalyzed Oxidative Arylation of Quinoxalin-2(1*H*)-ones with Arylboronic Acids. *Org Lett*. 15, 5606-5609. doi: 10.1021/ol4028946
